# Comparing physician associates and foundation year two doctors-in-training undertaking emergency medicine consultations in England: a mixed-methods study of processes and outcomes

**DOI:** 10.1136/bmjopen-2020-037557

**Published:** 2020-09-01

**Authors:** Mary Halter, Vari Drennan, Chao Wang, Carly Wheeler, Heather Gage, Laura Nice, Simon de Lusignan, Jonathan Gabe, Sally Brearley, James Ennis, Phil Begg, Jim Parle

**Affiliations:** 1 Faculty of Health, Social Care and Education, Kingston University and St George's, University of London, London, UK; 2 Centre for Medication Safety and Service Quality, Imperial College Healthcare NHS Trust, London, UK; 3 School of Economics, University of Surrey, Guildford, UK; 4 Institute of Clinical Sciences, University of Birmingham, Birmingham, UK; 5 Nuffield Department of Primary Care Health Sciences, University of Oxford, Oxford, UK; 6 School of Law and Social Science, Royal Holloway University of London, Egham, UK; 7 Chester Medical School, University of Chester, Chester, UK; 8 Royal Orthopaedic Hospital, Birmingham, UK

**Keywords:** accident & emergency medicine, organisation of health services, quality in health care

## Abstract

**Objectives:**

To compare the contribution of physician associates to the processes and outcomes of emergency medicine consultations with that of foundation year two doctors-in-training.

**Design:**

Mixed-methods study: retrospective chart review using 4 months’ anonymised clinical record data of all patients seen by physician associates or foundation year two doctors-in-training in 2016; review of a subsample of 40 records for clinical adequacy; semi-structured interviews with staff and patients; observations of physician associates.

**Setting:**

Three emergency departments in England.

**Participants:**

The records of 8816 patients attended by 6 physician associates and 40 foundation year two doctors-in-training; of these n=3197 had the primary outcome recorded (n=1129 physician associates, n=2068 doctor); 14 clinicians and managers and 6 patients or relatives for interview; 5 physician associates for observation.

**Primary and secondary outcome measures:**

The primary outcome was unplanned re-attendance at the same emergency department within 7 days. Secondary outcomes: consultation processes, clinical adequacy of care, and staff and patient experience.

**Results:**

Re-attendances within 7 days (n=194 (6.1%)) showed no difference between physician associates and foundation year two doctors-in-training (OR 0.87, 95% CI 0.61 to 1.24, p=0.437). If seen by a physician associate, patients were more likely receive an X-ray investigation (OR 2.10, 95% CI 1.72 to 4.24), p<0.001), after adjustment for patient characteristics, triage severity of condition and statistically significant clinician intraclass correlation. Clinical reviewers found almost all patients’ charts clinically adequate. Physician associates were evaluated as assessing patients in a similar way to foundation year two doctors-in-training and providing continuity in the team. Patients were positive about the care they had received from a physician associate, but had poor understanding of the role.

**Conclusions:**

Physician associates in emergency departments in England treated patients with a range of conditions safely, and at a similar level to foundation year two doctors-in-training, providing clinical operational efficiencies.

**Strengths and limitations of this study**

- This study provides a well-powered quantitative comparative analysis of the documented processes and outcomes of patient care by physician associates and foundation year two doctors-in-training in three emergency departments in different parts of England.
- We believe this to be the first empirical study of the outcomes of care provided by UK-trained physician associates in the emergency department, and the first internationally to include interview and observation data.
- Patients’ views have not been previously reported for physician associates in this setting.
- The low sensitivity of the emergency department triage system to identify conditions other than the most serious was a problem and impaired the study’s ability to describe case mix fully.

## Introduction

Healthcare systems internationally are challenged to ensure good patient outcomes, within financial constraints, as well as to attend to the work life of the workforce.^1^ Health workforce shortages, particularly of doctors, are resulting in the development of advanced clinical practitioners or non-physician clinicians (NPCs), such as nurse practitioners (NPs) and physician assistants/associates (PAs) in many countries.^2^ Numerous countries are experiencing rising patient demand for emergency services and concomitant shortages of doctors in emergency medicine.^3–7^ This situation has led to the development of NPC roles in emergency departments (EDs) in many countries such as the USA,^8^ Australia,^9^ Canada^10^ and the UK.^11^ In the USA, 25% (n=14 360) of all emergency medicine clinicians are NPCs, and 68% of these are PAs.^8^

PAs are trained in the medical model to take histories, diagnose illness, develop management plans and prescribe medications as agreed with their supervising physician. PAs have a 50-year history in the USA and are a developing part of the workforce in some other countries such as Canada, the Netherlands and Germany.^12^ The PA workforce is growing in the UK (where they are known as physician associates). In 2018, there was an estimated 600 qualified PAs with approximately 1000 graduating each year since then.^11^ Their employment specialties include EDs,^11^ where they are deployed in both the minor and the major illness or injury sections.^8^

Descriptive observations have been published concerning the positive contributions by US-trained PAs employed in EDs in the UK,^13^ Australia and New Zealand,^14^ and by UK-trained PAs in England.^15^ Unlike in the USA, PAs in these other countries cannot prescribe medicines or order ionising radiation. PAs in North American EDs are reported to be well accepted by other staff and patients, and reliable in assessing certain medical complaints and performing procedures.^16^ No difference is reported between patients attended by a PA and those attended by a doctor for wound infection rates, or rate of revisit within 72 hours to a paediatric ED; but studies find less consistency in practice when analysing prescribing patterns, length of stay and wait times of physicians, PAs and NPs in the ED.^17^ There is relatively little research evidence on their clinical effectiveness,^17^ little quantitative evidence on outcomes from outside of the USA and no qualitative evidence of how PAs deliver care in the ED. In this context, our goal was to investigate the contribution of PAs to the processes and outcomes of emergency medicine consultations compared with that of foundation year two (FY2) doctors-in-training in EDs in English hospitals.

## Methods

### Study design

We conducted a pragmatic, mixed-methods convergence study in which we compare and contrast and simultaneously interpret quantitative and qualitative data^18^ in three EDs in England, with three components. We undertook a quantitative observational retrospective chart review of patient consultations by PAs compared with FY2 doctors-in-training; and qualitatively we directly observed PAs’ practice; and we conducted semi-structured interviews with members of the staff team. Our planned prospective study of patient records with a linked patient satisfaction and outcomes survey had to be revised to a pragmatic retrospective chart review due to practicalities within the participating National Health Service (NHS) organisations in the period of the study.

### Population and sampling

Three consultant-led, 24 hours EDs with full resuscitation facilities (‘type one’) participated. Two EDs had annual attendances in the range of 1 00 000–1 20 000 adult and paediatric patients and the third in the range of 1 70 000–1 90 000. One was an university hospital; two were district general hospitals. The hospitals had been recruited as part of a larger study investigating the work and contribution of PAs between 2016 and 2017.^19^ We selected FY2 doctors-in-training as the comparator for PAs, as PAs are offered as part of a solution to junior medical workforce shortages^7^ and the most junior doctors working in the UK ED at the time were FY2s.

### Selection of participants, measurements and outcomes

Our primary outcome was unplanned re-attendance at the same ED within 7 days—one of the NHS clinical quality indicators for EDs in England.^20^ Our secondary outcomes were: consultation processes (length of time in the ED, use of X-ray, prescriptions and referrals); clinical adequacy of care, referrals and planned follow-up;and patient experience.

#### Chart review

For a 16-week period (the standard duration of ED placement for FY2 doctors-in-training in the UK), we obtained anonymised, routinely collected electronic records of all patients attended by a PA or FY2 doctor-in-training, provided in Microsoft Excel by the hospital information teams in each trust, using queries based on staff job role, dates and requested data items. Hospital staff extracted additional data items (online supplementary material 1)—age, sex, acuity (as categorised by the Manchester triage score^21^), X-ray orders, diagnosis, prescription issued, admission, area treated, overall time in the ED (from check in to discharge, in minutes) and re-consultation within 7 days (the primary outcome). No data linkage was required. The researchers did not have access to the original dataset and so could not identify if any patients appeared more than once in the dataset, and further data cleaning could not be performed.

10.1136/bmjopen-2020-037557.supp1 Supplementary data

We calculated a sample size for the primary outcome based on rate of 18.3% (the highest of two rates for nurse practitioners substituting for physicians (at 28 days)).^22 23^ Aiming to find a relative difference of 50%, in a non-inferiority hypothesis, we required 284 patients in each group (calculation from Stata V.11.1 software) to compare 18.3% to 27.4% unplanned re-consultations, with conventional 80% power at 5% significance. We included an extra 20 to allow for adjustment for case mix, requiring a minimum of 304 patients in total in each group to achieve the said power. As 28-day data could not be collected, we went on to use the 7-day re-attendance rate, with its national average of 7.4% (range 2.4%–21.7%) for unplanned re-attendance at the same ED in England for all patients.

Two of the participating EDs also agreed to take part in the analysis of clinical adequacy of documented care in every 10th case from the chart review sample (n=40), with equal numbers of cases seen by PA and FY2 doctors-in-training, and using the full anonymised clinical record. We recruited two specialty registrars (doctors in their 6th year of emergency medicine training), one PA lecturer (with 20 years ED experience) and one emergency medicine consultant (with 17 years experience at consultant level) from outside the three study hospitals to review these records. All four clinicians independently recorded their judgement as to the clinical adequacy of care for each record using the categories of medical history, examination, request for radiography, treatment plan and decision, advice given and follow-up. Their assessments were blinded to the type of professional attending the patient and to each other’s assessment, using a proforma (online supplementary material 2) based on published studies.^22 23^ As the senior clinician, we accepted the decision of the consultant in cases of disagreement.

#### Observation

This element drew on the ethnographic tradition used in many health service research studies.^24^ We invited all PAs working in the ED in our three study hospitals to participate (n=6). Five PAs volunteered and gave written informed consent to be observed. One of three researchers (CWh, LN, MH) observed each PA for two or three pre-arranged sessions, of varying lengths, on weekdays in periods between 08:00 and 22:00 hours, following a broad guide (online supplementary material 3). Researchers made notes on context, relationships and activities following this guide. We judged data saturation to have been reached with individual PAs when the processes of care observed did not differ significantly from previous observations. During the observation period, PAs asked for patient assent to the researcher’s presence. Researchers reflected on the observations, discussing them in pairs.

#### Interview

Semi-structured interviews^25^ were undertaken with a purposive sample of managerial, medical and nursing ED staff who volunteered after receiving information about the study from the researcher during observation periods and/or via their site manager. We also opportunistically interviewed patients and/or their relative who were being seen by a PA in the ED, identified and invited to participate during observation periods, once they had been assessed and treated by the PA but before discharge from the ED. We used tailored topic guides (online supplementary material 4) to explore interviewees’ perceptions of the PA role and its impact on service organisation, role boundaries, patient experience, patient outcomes and activities and attitudes of other staff. We digitally recorded interviews or took notes if the participant preferred. Recordings were transcribed verbatim and anonymised.

### Analysis

#### Chart review

The characteristics of patients treated by PAs and FY2 doctors-in-training were compared using χ^2^ tests. We carried out a logistic regression to examine whether the primary and binary secondary outcomes differed between PAs and FY2 doctors-in-training, while adjusting for confounding factors—patient age, sex and triage score. Since patients seen by the same clinician are likely to be correlated, we calculated intraclass correlation coefficients (ICC) for each outcome and report results using a random-effects model if the ICC is statistically significant. We report ORs, their CIs and two-tailed p values. For length of stay, a linear regression was used for data transformed to logarithm scale to reduce heteroscedasticity and reflect the fact that the value of length of stay is positive. To account for unobserved heterogeneity, the unobserved component is modelled as a latent variable in a latent class linear model. The assessment of clinical adequacy is reported using descriptive statistics, sensitivity and specificity of the judgement of whether the record was that of a PA or FY2 doctor-in-training and Fleiss kappa for inter-rater agreement, calculated for each of the four components of the assessment and per response.

#### Qualitative

Our methods for the analysis of observation data drew on methods to identify ethnographic vignettes.^26^ We employed thematic analysis^27^ of all-specialty interview data for the wider study. Both are described in full elsewhere.^19^ For the subsequent specialty-specific analysis, we re-read all ED observation data and interview transcripts to identify all data related to the primary and secondary outcomes, and which both confirmed or disconfirmed findings.

#### Mixed methods

Following the separate quantitative and qualitative analyses, we (MH and VD, in consultation with all authors) merged^18^ the quantitative and qualitative datasets by presenting the quantitative results by study outcomes and following these with qualitative data findings (themes and/or excerpts or quotes) that confirmed or disconfirmed the quantitative results.

### Patient and public involvement

The patientand public voice was important to this study and informed the design, conduct, analysis, interpretation and final reporting. We brought the views of our public and patient representative forum for a previous study on physician associates into the research questions and design of the study. These were views such as how do patients understand this new role. Sally Brearley, as a public voice representative, was a co-applicant and member of the research team. The study advisory group had two public voice members who were reimbursed for their time, following NIHR INVOLVE guidance. Two patient and public voice groups were formed: one in London and the other in the West Midlands and members reimbursed as per NIHR Involve guidelines. The patient and public voice groups informed the design of the research tools such as topic guides and participant information sheets, developed coding frameworks and analysed interview transcripts, and participated in the overall synthesis of findings. Sally Brearley continues to be involved in the dissemination of the study.

## Results

### Characteristics of chart review subjects

In the 16-week period studied, 8816 patients seen by 6 PAs (n=2890) or 40 FY2 doctors-in-training (n=5926) were identified; some secondary outcomes were available for all cases. For 3197 of these patient episodes (n=1129 by the 6 PAs and n=2068 by 22 FY2 doctors-in-training), the primary outcome was collected at site for the research team. Characteristics of the patients are shown in table 1. PAs saw a lower proportion of patients categorised on triage into the urgent category than FY2 doctors-in-training.

**Table 1:** Characteristics of chart review sample

Characteristic	PA (n=2381)	FY2 doctor (n=6435)	Total (n=8816)	P value
	N (%)	N (%)	N (%)	
Age band (years)				
0–20	300 (13.0%)	656 (10.3%)	956 (11.0%)	0.002
21–40	543 (23.5%)	1493 (23.5%)	2036 (23.5%)	
41–60	530 (22.9%)	1406 (22.1%)	1936 (22.3%)	
61–80	551 (23.8%)	1596 (25.1%)	2147 (24.7%)	
81 and over	390 (16.9%)	1212 (19.0%)	1602 (18.5%)	
Sex				
Male	1132 (47.5%)	2933 (45.6%)	4065 (46.1%)	0.102
Female	1249 (52.5%)	3501 (54.4%)	4750 (53.9%)	
Manchester triage score				
1 Immediate	10 (0.6%)	3 (0.1%)	13 (0.2%)	<0.001
2 Very urgent	163 (9.3%)	565 (11.1%)	728 (10.6%)	
3 Urgent	770 (43.8%)	2841 (55.7%)	3611 (52.6%)	
4 Standard	811 (46.1%)	1681 (32.9%)	2492 (36.3%)	
5 Non-urgent	5 (0.3%)	12 (0.2%)	17 (0.2%)	
ED area treated in				
Minor	369 (20.1%)	275 (6.8%)	644 (10.9%)	<0.001
Major	1266 (68.8%)	3601 (88.4%)	4867 (82.3%)	
Resuscitation	2 (0.1%)	4 (0.1%)	6 (0.1%)	
Paediatrics	181 (9.8%)	174 (4.3%)	355 (6.0%)	
Clinical decision unit or primary care	21 (1.1%)	20 (0.5%)	41 (0.7%)	

FY2, foundation year two; PA, physician associates.

In interview, the type of patient seen, patient throughput and role of PAs and FY2 doctors-in-training were described as similar:

They’re [the PAs] pretty much equal to ……a senior FY2 doctor in training level. As a consultant we feel comfort because we know [PA name 1] can work in majors, she can clear [majors] pretty much…… And [PA name 2],… can clear paeds minors… Participant 150 emergency medicine consultant

However, more than one participant tentatively suggested that PAs saw the less acutely unwell patients:

So my understanding is like they’re [the PAs] equivalent to, I would put it like a certain level of like a junior physician……I wouldn’t say they would be at registrar level……I’d put them somewhere in between. You know a…lot better than like a newly qualified physician because they’ve got the skills and stuff, so in that gap of what I would say equivalent to maybe like a second to four years post qualified doctor. Participant 144 registrar

### Characteristics of interview and observation participants

The staff interviewed included four PAs, two managers, five nurses and three senior doctors; six patients and/or their relatives were also interviewed, spread across the three sites. We observed four PAs, at three sites; we do not report further demographic details due to concerns about anonymity in a small population.

### The primary outcome: rate of return to the ED within 7 days

Re-attendance within 7 days was found following 6.1% (n=194) of the 3197 index visits for which these data were available. The high rate of unknown data is accounted by one site where these data were not captured in the electronic dataset and were only retrieved manually for a random sample (n=205) for the purposes of this study. After adjustment for confounding, no statistically significant difference was found for cases seen by PAs or FY2 doctors-in-training (table 2).

**Table 2:** Re-attendance at the same ED within 7 days

Re-attendance at the same ED within 7 days	PA (n=1129)	FY2 doctor (n=2068)	Total (n=3197)	Unadjusted OR (95% CI) and p value (PA relative to FY2 doctor-in-training) in rate of re-attendance	Adjusted OR (95% CI) and p value (PA relative to FY2 doctor-in-training) in rate of re-attendancee*
No	1066 (94.4%)	1937 (93.7%)	3003 (93.9%)	0.87 (0.64 to 1.19) p=0.388	0.87 (0.61 to 1.24) p=0.437
Yes	63 (5.6%)	131 (6.3%)	194 (6.1%)		
Unknown	1251	4368	5619	–	–

*Adjustment made for triage score (as a measure of acuity), age band, sex, admission, X-ray and site; no adjustment was made for clustering as the ICC by individual staff member on outcome was small (0.008) and statistically insignificant (p=0.236).

ED, emergency department; FY2, foundation year two; ICC, intraclass correlation coefficient; PA, physician associates.

### Secondary outcome: consultation processes

No differences were found between patients attended by PAs or by FY2 doctors-in-training in: whether prescriptions were given, admission to hospital from the ED or if a discharge summary was completed. However, patients seen by a PA were more likely to have an X-ray performed in the ED (table 3), less likely to be admitted to hospital and to have a shorter length of stay in the ED (by 35 min), after adjustment for age, sex, acuity, whether admitted, X-ray taken and site, as well as for clustering by individual clinician, although no account was able to be taken of the staffing level.

**Table 3:** Clinical process measures

Clinical process measure	PA (n=2381)	FY2 doctor (n=6435)	Total (n=8816)	Unadjusted OR (95% CI) and p value (PA relative to FY2 doctor-in-training) in rate of re-attendance	Adjusted OR (95% CI) and p value (PA relative to FY2 doctor-in-training) in rate of re-attendancee*
X-ray investigations performed					
No	559 (49.4%)	1701 (82.3%)	2260 (70.7%)	4.76 (4.04 to 5.59) p<0.001	2.70 (1.72 to 4.24) p<0.001
Yes	572 (50.6%)	366 (17.7%)	938 (29.3%)		
Unknown	1250	4368	5618	–	–
Prescriptions given in the ED					
No	174 (58.0%)	157 (51.8%)	331 (54.9%)	0.79 (0.56 to 1.07) p=0.127	1.35 (0.08 to 23.5) p=0.838
Yes	126 (42.0%)	146 (48.2%)	272 (45.1%)		
Unknown	2081	6132	8213		
Admitted as an inpatient from the ED					
No	883 (78.2%)	1436 (70.1%)	2319 (73.0%)	0.65 (0.55 to 0.77) p<0.001	0.78 (0.55 to 1.1) p=0.158
Yes	246 (21.8%)	613 (29.9%)	859 (27.0%)		
Unknown	1762	3876	5638		
Discharge summary completed					
No	86 (42.4%)	71 (34.6%)	157 (38.5%)	0.72 (0.48 to 1.08) p=0.109	1.57 (0.93 to 2.66) p=0.09
Yes	117 (57.6%)	134 (65.4%)	251 (61.5%)		
Unknown	2178	6230	8408		

*Adjustment made for MTS (as a measure of acuity), age band, sex and site, and for clustering where the ICC (and p value) is significant: X-ray 0.04 (p<0.001), prescriptions 0.73 (p<0.001), admitted 0.02 (p=0.001), discharge summary <0.001 (p=0.498).

FY2, foundation year two; ICC, intraclass correlation coefficient; PA, physician associates.

We observed PAs being the first member of the medical team to carry out assessment of patients following triage to either the major, minor or paediatric areas of the ED. We noted that PAs saw patients independently, following a medical history taking and examination model, before reporting in person to the senior ED physician in the same way as nurse practitioners and FY2 doctors-in-training did.

PAs were differentiated from FY2 doctors-in-training by many of our interviewees for not being able to prescribe medications or order tests using ionising radiation. Some participants considered this to have a detrimental impact on PAs and patients:

[prescribing] would make a massive difference for them as well and [for] patients because at the end of the day they’re having to wait for the PAs to go talk through [with] the physicians what’s going on and then probably see somebody else. Participant 118 nurse practitioner

However, PAs were observed taking on several roles in relation to prescriptions and X-ray orders, for example, suggesting medications to or charting the medication for a senior doctor to sign off:

So when one of my PAs comes to me and says ‘This patient has a temperature of 38, they’re coughing up horrible green sputum and they’re tachycardic and I listened to their chest and they’ve got crackles at the left base, can we order a chest x-ray and prescribe sepsis drugs for, you know, pneumonia?’ I say ‘Yes’ and I sign it. With probably more confidence at this stage having had [number] PAs here for a year than I would with a junior physician in training on day two. And the irony of that is of course, the junior physician in training doesn’t need to come and ask me, technically, they can prescribe themselves. Participant 21 emergency medicine consultant

PAs were also observed making referrals to medical and surgical teams outside of the ED, completing discharge summary information and carrying out procedures, most commonly cannulation, phlebotomy and suturing.

### Secondary outcome: clinical adequacy

Our reviewers found the chart documentation to have been ‘appropriate’ or ‘with no errors or omissions that resulted in significant probability that the patient might be harmed’ in 36/40 cases for all of the key consultation components (table 4). In the three records (two of FY2 doctors-in-training and one of a PA) judged as having errors or omissions at the level of a breach in normal guidelines and procedures that would have altered the patient’s treatment, all reviewers agreed that a senior doctor review had occurred in one case; this was unclear in the other cases. Our observation data suggest that such a senior review was undertaken for all assessment and clinical decision making in the ‘majors’ sections of the ED, but that ‘minors’ care was often completed independently.

**Table 4:** Chart reviewers’ assessments of clinical adequacy

PA or FY2 consultation record		Judgement of appropriateness																							
		Medical history				Examination				Request for radiography*				Treatment plan and decision				Advice given				Follow-up			
		Appropriate	Error or omission			Appropriate	Error or omission			Appropriate	Error or omission			Appropriate	Error or omission			Appropriate	Error or omission			Appropriate	Error or omission		
			Harm unlikely	Altered treatment	Harm		Harm unlikely	Altered treatment	Harm		Harm unlikely	Altered treatment	Harm		Harm unlikely	Altered treatment	Harm		Harm unlikely	Altered treatment	Harm		Harm unlikely	Altered treatment	Harm
FY2	n	14	6	0	0	15	5	0	0	9	0	1	0	14	5	1	0	4	1	1	0	16	3	0	0
	%	70	37	0	0	75	25	0	0	45	0	5	0	70	25	5	0	20	5	5	0	80	15	0	0
PA	n	13	5	1	0	11	7	1	0	9	3	0	0	13	5	1	0	3	1	1	0	13	4	1	0
	%	65	25	5	0	55	35	5	0	45	15	0	0	65	25	5	0	15	5	5	0	65	20	5	0
Not rated*	n	1				1				18				1				29				3			
	%	2.5				2.5				45				2.5				73				7.5			
Total	n	27	11	1	0	26	12	1	0	18	3	1	0	27	10	2	0	7	2	2	0	29	7	1	0
	%	68	28	3	0	65	30	3	0	45	8	3	0	66	25	5	0	18	5	5	0	73	18	3	0
Agreement (Fleiss kappa, combined)		0.01				0.15				0.26				0.15				−0.03				0.30			
Agreement (Fleiss kappa, per response)		0.04	−0.02	−0.04	n/a	0.17	0.12	0.15	n/a	0.29	0.11	0.33	n/a	0.24	0.01	0.14	n/a	−0.00	0.11	0.08	n/a	0.36	0.20	0.28	n/a

*Missing rating or rated as ‘not applicable’ if no request for radiography was made or no advice given.

FY2, foundation year two; PA, physician associates.

Our reviewers were 40% sensitive, 46% specific on judging the clinician type: 68% (13/19) of the PA records were thought to be of a FY2 doctor-in-training and 60% (9/15) vice versa (kappa score for inter-rater agreement 0.15).

Interviewees also presented other aspects related to clinical adequacy, particularly the PAs’ stability in the team. The clinicians’ familiarity with the longer standing team member PA/s—in contrast to FY2 doctors-in-training on rotation—was raised repeatedly:

If there was a junior physician over here, and he said oh, what do you think of this wound, which they do ask us. And I say yeah, it needs suturing. I then have to say, but can you suture or do you want me to suture it?……Because I don’t know, and some will say oh no, I can’t……I’ve never sutured before, and some will say oh yeah, that’s fine, I’ll suture it……Whereas I know with PAs they’ll suture their own. Because I know that they’ve got that skill set. Participant 177 advanced nurse practitioner

### Secondary outcome: patient experience

Patients were positive about the care they had received from the PA, but had not understood what the PA role meant, with two participants believing they had been seen by a doctor and another unsure in the context of multiple ED staff:

I presumed he was a fully-qualified physician, yes his approach and everything was absolutely 100%. Participant 120 patient

Most of our patient participants were receptive to the role on the grounds that it might speed up care, although they were not without concern for the difference in training from a doctor and the diminishment of a senior medical workforce:

It’s good to have another person, another opinion…but would it not perhaps be better to have another doctor? Participant 083 patient’s relative

## Discussion

### Summary of findings

The study presents evidence from three English EDs and has demonstrated no difference in safety or appropriateness between PAs and FY2 doctors-in-training. We report no difference in re-attendance rates. Those patients seen by a PA (within PA working hours 08:00–22:00) had a shorter average length of stay in the ED than those seen by doctors-in-training (24 hours working period). Our review of clinical adequacy found few errors and no difference between PAs and FY2 doctors-in-training. Patients appeared relatively unconcerned with the title of the clinician treating them and thought they had been treated by a doctor; however, they were keen to know that the employment of PAs would not represent a widespread substitution for doctors in the ED.

### How this study is similar or different from prior studies

We believe this to be the first empirical study of the outcomes of care provided by UK-trained PAs in the ED, and the first internationally to include interview and observation data. Additionally, patients’ views do not appear to have been previously gathered at the time of the visit (and qualitatively), although there have been previous questionnaire studies in the USA of patient satisfaction, administered after the visit.^28–30^

We reported few differences in the the practice and processes of care—other than prescribing (which PAs currently cannot do independently in the UK)—between PAs and doctors in their second foundation year of training. Our finding of no difference in the primary outcome (ED re-attendance rate within 7 days) for patients of PAs and FY2 doctors-in-training is consistent with the comparisons of nurse-qualified NPCs and FY2 doctors-in-training on which we based our study design^22 23^ and other PA literature from the USA.^16^ It should be noted that for patients in the majors section of ED, all assessment and treatment plans by FY2 doctors-in-training and NPCs were reviewed and agreed by a senior clinician. Our participants commented frequently on the transient nature of FY2 doctors-in-training, whose rotation in the ED only last 4 months. In contrast, PAs remained long-term and provided continuity in the team. Their accumulated knowledge of the policies and practices (clinical and otherwise) of the department, the consultants and the hospital was reported to enable operational efficiencies. Similar observations about PAs providing continuity within the medical/surgical team have been made in North America and the Netherlands^31–33^ and also for other NPCs.^34^

This study’s strengths lie in its mixed-methods approach to the study of PAs in the ED, allowing consideration of different types of data on their contribution, compared with that of FY2 doctors-in-training, to be considered. We were able to carry out a well-powered quantitative comparative analysis of the documented processes and outcomes of patient care by PAs and FY2 doctors-in-training in three EDs in different parts of the country, and to gather qualitative data on PAs ‘in practice’. The qualitative component of our mixed-methods approach enabled contextual explanations of the quantitative analysis.

Our study however has several limitations. Our comparison of PAs and doctors working in all areas of the ED introduced the potential for PAs and FY2 doctors-in-training to be attending to patients of different acuity and complexity. We sought to mitigate this by using three different EDs, taking a sample across a 16-week period at all times of day and night (although the FY2 doctors-in-training worked over the 24 hours period when staff:patient ratios may have fluctuated). We also made statistical adjustments that included triage category. The low sensitivity of most ED triage systems to identification of conditions other than the most serious, however, is a drawback.^35^ The prevention of collection of 28-day outcomes by NHS organisations was also a barrier, particularly as we had based our sample size calculation on that, as opposed to the lower 7-day return rate.

The level of missing data for some variables in the routinely collected data, and not having data from which to take into account whether PA reduced the staff:patient ratio (or fully replaced FY2 doctors-in-training) is a further limitation and needs to be borne in mind in the comparisons we present. Likewise, our observation data illustrated care is predominantly delivered by teams which creates difficulties in attributing outcomes or processes to individual staff, and compromised our ability to undertake an economic evaluation.

Our interview invitations yielded relatively small numbers of participants, particularly among patients/relatives. While we attribute this in part to the fast patient throughput of the ED and limited availability of the researcher, this limits our analysis.

### Implications for policy and practice

PAs in the ED are acceptable to patients and can help to relieve staffing pressures and improve efficiency in the delivery of care. They are able to treat patients safely with a range of conditions and FY2 doctors-in-training deliver similar care to that provided by doctors in their second year of training. Deployment of PAs within ED teams is a potential solution to the situation of growing patient demand and predicted shortage of junior doctors in the British NHS,^7^ of which FY2 doctors on rotation in specialties such as the ED are one part; it is not our intention to raise or limit PAs to one particular junior doctor comparator level, but we have used this here as the closest pragmatic comparator. An alternative, which is to hire locum doctors, comes at a higher costs and loss of team continuity, and has potential implications for patient safety. Moves to regulate the PA profession under the General Medical Council were started in 2019.^36^

The findings of this study support employment of appropriately trained, supervised PAs with professional registration in ED teams. Further research is needed to investigate fully the impacts we have observed, particularly the cost-effectiveness.

## Supplementary Material

Reviewer comments

Author's manuscript

## References

[1] Bodenheimer T, Sinsky C From triple to quadruple aim: care of the patient requires care of the provider. Ann Fam Med 2014;12:573–6. 10.1370/afm.1713 25384822 PMC4226781

[2] World Health Organization Task shifting: rational redistribution of tasks among health workforce teams – global recommendations and guidelines. Geneva: World Health Organization, 2008 www.who.int/healthsystems/task_shifting/en

[3] Reiter M, Wen LS, Allen BW The emergency medicine workforce: profile and projections. J Emerg Med 2016;50:690–3. 10.1016/j.jemermed.2015.09.022 26823136

[4] Takagi K, Tagami T Work-style reform of emergency physicians: the Japanese experience. Eur J Emerg Med 2019;26:398–9. 10.1097/MEJ.0000000000000640 31688220

[5] Suriyawongpaisal P, Atiksawedparit P, Srithamrongsawad S, et al Assessment of nationwide emergency systems in Thailand, a middle-income country setting with UHC. Int J Health Plann Manage 2019;34:e1346–55. 10.1002/hpm.2783 30945365

[6] Collaborative Working Group on the Future of Emergency Medicine in Canada (CWG-EM):, Sinclair D, Abu-Laban RB, et al Emergency Medicine Training and Practice in Canada: Celebrating the Past & Evolving for the Future. CJEM 2017;19:S1–8. 10.1017/cem.2017.372 28756801

[7] The Royal College of Emergency Medicine and the NHS Securing the future workforce for emergency departments in England, 2017 Available: https://improvement.nhs.uk/documents/1826/Emergency_department_workforce_plan_-_111017_Final.3.pdf [Accessed 31 Jul 2019].

[8] Hall MK, Burns K, Carius M, et al State of the National emergency department workforce: who provides care where? Ann Emerg Med 2018;72:302–7. 10.1016/j.annemergmed.2018.03.032 29753519

[9] Gardner G, Gardner A, Middleton S, et al Mapping workforce configuration and operational models in Australian emergency departments: a national survey. Aust Health Rev 2018;42:340–7. 10.1071/AH16231 28514641

[10] Thrasher C, Purc-Stephenson R Patient satisfaction with nurse practitioner care in emergency departments in Canada. J Am Acad Nurse Pract 2008;20:231–7. 10.1111/j.1745-7599.2008.00312.x 18460162

[11] Ritsema T The faculty of physician associates census results 2018. Faculty of physician associates at the Royal College of physicians. Available: http://www.fparcp.co.uk/about-fpa/fpa-census [Accessed 10 Sep 2019].

[12] Cawley JF, Hooker RS Determinants of the physician assistant/associate concept in global health systems. Int J Healthc 2018;4:50–60. 10.5430/ijh.v4n1p50

[13] Farmer J, Currie M, Hyman J, et al Evaluation of physician assistants in national health service Scotland. Scott Med J 2011;56:130–4. 10.1258/smj.2011.011109 21873716

[14] Close B, Zolcinski R Physician assistants in Australasian emergency departments. Emerg Med Australas 2016;28:475–7. 10.1111/1742-6723.12607 27198197

[15] Howie N A new kid on the block: the role of physician associates. Clin Med 2015;14, 6:309.2–10. ·. 10.7861/clinmedicine.15-3-309a PMC4953125 26031991

[16] Doan Q, Sabhaney V, Kissoon N, et al A systematic review: the role and impact of the physician assistant in the emergency department. Emerg Med Australas 2011;23:7–15. 10.1111/j.1742-6723.2010.01368.x 21284809

[17] Halter M, Wheeler C, Pelone F, et al Contribution of physician assistants/associates to secondary care: a systematic review. BMJ Open 2018;8:e019573. 10.1136/bmjopen-2017-019573 PMC6020983 29921680

[18] Creswell JW, Clark VL Designing and conducting mixed methods research. Thousand Oaks, CA: Sage Publications Ltd, 2007.

[19] Drennan VM, Halter M, Wheeler C, et al What is the contribution of physician associates in hospital care in England? a mixed methods, multiple case study. BMJ Open 2019;9:e027012. 10.1136/bmjopen-2018-027012 PMC6359738 30700491

[20] NHS Digital Provisional Accident and Emergency Quality Indicators - England, 2018 Available: https://digital.nhs.uk/data-and-information/publications/statistical/accident-and-emergency-quality-indicators/april-2018-by-provider [Accessed 2 Aug 2018].

[21] Mackway Jones K, Marsden J, Windle J Emergency triage: Manchester triage group. 3rd edn London: John Wiley & Sons, 2013.

[22] Sakr M, Angus J, Perrin J, et al Care of minor injuries by emergency nurse practitioners or junior doctors: a randomised controlled trial. Lancet 1999;354:1321–6. 10.1016/S0140-6736(99)02447-2 10533859

[23] Cooper MA, Lindsay GM, Kinn S, et al Evaluating emergency nurse practitioner services: a randomized controlled trial. J Adv Nurs 2002;40:721–30. 10.1046/j.1365-2648.2002.02431.x 12473052

[24] Pope C, Mays N Observational Methods : Pope C, Mays N, Qualitative research in health care. 4th edn Chichester: John Wiley & Sons, 2008: 32–42.

[25] Kvale S Doing interviews. London: Sage Publications, 2007 http://methods.sagepub.com/book/doing-interviews

[26] McCann L, Granter E, Hyde P, et al Still blue-collar after all these years? An ethnography of the professionalization of emergency ambulance work. J Manage Stud 2013;50:750–76. 10.1111/joms.12009

[27] Boyatzis RE Transforming qualitative information: thematic analysis and code development. Thousand Oaks, Calif: Sage, 1998.

[28] Maxfield RG Use of physician's assistants in a general surgical practice. Am J Surg 1976;131:504–8. 10.1016/0002-9610(76)90165-3 4985

[29] Rodi SW, Grau MV, Orsini CM Evaluation of a fast track unit: alignment of resources and demand results in improved satisfaction and decreased length of stay for emergency department patients. Qual Manag Health Care 2006;15:163–70. 10.1097/00019514-200607000-00006 16849988

[30] Berg GM, Crowe RE, Nyberg S, et al Trauma patient satisfaction with physician assistants: testing a structural equation model. J Am Acad Physician Assist 2012;25:42–3. 10.1097/01720610-201205000-00008 22712148

[31] Fréchette D, Shrichand A Insights into the physician assistant profession in Canada. J Am Acad Physician Assist 2016;29:35–9. 10.1097/01.JAA.0000484302.35696.cd 27351645

[32] Kartha A, Restuccia JD, Burgess JF, et al Nurse practitioner and physician assistant scope of practice in 118 acute care hospitals. J Hosp Med 2014;9:615–20. 10.1002/jhm.2231 25224593

[33] Timmermans MJC, van Vught AJAH, Maassen ITHM, et al Determinants of the sustained employment of physician assistants in hospitals: a qualitative study. BMJ Open 2016;6:e011949. 10.1136/bmjopen-2016-011949 PMC5128943 27864243

[34] Williams K Advanced practitioners in emergency care: a literature review. Emerg Nurse 2017;25:36–41. 10.7748/en.2017.e1685 28703051

[35] Hinson JS, Martinez DA, Cabral S, et al Triage performance in emergency medicine: a systematic review. Ann Emerg Med 2019;74:140–52. 10.1016/j.annemergmed.2018.09.022 30470513

[36] General Medical Council GMC to regulate two new associates roles, 2019 Available: https://www.gmc-uk.org/news/news-archive/gmc-to-regulate-two-new-associates-roles-pas-and-aas [Accessed Jul 2019].

