# Supplementary material for: Comparing physician associates and foundation year two doctors-in-training undertaking emergency medicine consultations in England: a mixed-methods study of processes and outcomes
Source: BMJ Open. 2020 Sep 1;10(9):e037557. doi: 10.1136/bmjopen-2020-037557 (PMC7467515; doi:10.1136/bmjopen-2020-037557)
Supplement: Supplementary data [file bmjopen-2020-037557supp001.pdf]

Supplementary file 1: Extract of the chart review dataset (first 50 cases, according to date of attendance)

| study_id | sample_case | site_numl | age_formatted | sex_formatted | arrival_date | arrival_time | seen_time | presenting_complaint | mts | ews | ed_strean | professior | xrays_forr | pxn_form | diagnosis | discharge_time | destination_grc | discharge_ | discharge_ | discharge_ | reattend_ | reattend_date |
|---|---|---|---|---|---|---|---|---|---|---|---|---|---|---|---|---|---|---|---|---|---|---|
| CS30115 | 0 | 3 | 5 | 0 | 05-Aug-16 | 9:10:00 PM | 10:56:00 PM | Flank Pain(renal colic 3yrs ago) | #NULL! | #NULL! | 1 | 1 | #NULL! | #NULL! | Renal colic | 2:07:00 AM | #NULL! | #NULL! | #NULL! | | #NULL! | #NULL! |
| CS30197 | 0 | 3 | 6 | 1 | 05-Aug-16 | 7:54:00 PM | 10:01:00 PM | Abdo/Back Pain 5/7 | 3 | #NULL! | #NULL! | 1 | #NULL! | #NULL! | Generally unwell | 1:40:00 AM | #NULL! | #NULL! | #NULL! | | #NULL! | #NULL! |
| CS30415 | 0 | 3 | 4 | 1 | 05-Aug-16 | 4:51:00 PM | 7:05:00 PM | 21/40 ?DVT on clexane | 3 | #NULL! | #NULL! | 1 | #NULL! | #NULL! | Acute coronary syndrome | 8:50:00 PM | #NULL! | #NULL! | #NULL! | | #NULL! | #NULL! |
| CS30514 | 0 | 3 | 5 | 1 | 05-Aug-16 | 1:08:00 PM | 2:15:00 PM | swelling to arm/hand ?clot | 3 | #NULL! | #NULL! | 1 | #NULL! | #NULL! | Deep venous thrombosis | 5:07:00 PM | #NULL! | #NULL! | #NULL! | | #NULL! | #NULL! |
| CS30726 | 0 | 3 | 3 | 0 | 05-Aug-16 | 11:45:00 PM | 1:12:00 AM | HEADACHE | 2 | #NULL! | 1 | 1 | #NULL! | #NULL! | Unknown problem | 1:58:00 AM | #NULL! | #NULL! | #NULL! | | #NULL! | #NULL! |
| CS30962 | 0 | 3 | 4 | 0 | 05-Aug-16 | 4:26:00 PM | 6:59:00 PM | Back Pain(mr maurice bulging disc) | 3 | #NULL! | #NULL! | 0 | #NULL! | #NULL! | Chronic back pain | 8:20:00 PM | #NULL! | #NULL! | #NULL! | | #NULL! | #NULL! |
| CS30993 | 0 | 3 | 4 | 1 | 05-Aug-16 | 6:57:00 AM | 8:04:00 AM | Epigastric Pain 13months on/off | 4 | #NULL! | #NULL! | 1 | #NULL! | #NULL! | Acute gastritis | 10:54:00 AM | #NULL! | #NULL! | #NULL! | | #NULL! | #NULL! |
| CS31043 | 0 | 3 | 9 | 1 | 05-Aug-16 | 10:49:00 PM | 12:00:00 AM | AP ?UTI | 4 | #NULL! | #NULL! | 1 | #NULL! | #NULL! | UTI - Urinary tract infection | 2:13:00 AM | #NULL! | #NULL! | #NULL! | | #NULL! | #NULL! |
| CS31165 | 0 | 3 | 3 | 1 | 05-Aug-16 | 9:56:00 PM | 11:31:00 PM | PV Bleed+cramps(bloods with gp nad) | 4 | #NULL! | #NULL! | 1 | #NULL! | #NULL! | Menorrhagia | 1:56:00 AM | #NULL! | #NULL! | #NULL! | | #NULL! | #NULL! |
| CS31275 | 0 | 3 | 8 | 1 | 05-Aug-16 | 8:55:00 PM | 10:50:00 PM | epigastric pain | 3 | #NULL! | 1 | 0 | #NULL! | #NULL! | | 12:38:00 AM | #NULL! | #NULL! | #NULL! | | #NULL! | #NULL! |
| CS31564 | 0 | 3 | 1 | 0 | 05-Aug-16 | 4:26:00 PM | 5:48:00 PM | not passing urine/feeding unsettled | 3 | #NULL! | #NULL! | 0 | #NULL! | #NULL! | Well child | 6:52:00 PM | #NULL! | #NULL! | #NULL! | | #NULL! | #NULL! |
| CS31572 | 0 | 3 | 3 | 0 | 05-Aug-16 | 11:20:00 PM | 12:43:00 AM | Sickle cell attack | 3 | #NULL! | #NULL! | 1 | #NULL! | #NULL! | Sickle cell anemia crisis | 3:18:00 AM | #NULL! | #NULL! | #NULL! | | #NULL! | #NULL! |
| CS31639 | 0 | 3 | 8 | 1 | 05-Aug-16 | 8:53:00 AM | 9:38:00 AM | HI, unwitnessed, stumbling on street | 3 | #NULL! | #NULL! | 1 | #NULL! | #NULL! | Head injury | 12:50:00 PM | #NULL! | #NULL! | #NULL! | | #NULL! | #NULL! |
| CS31640 | 0 | 3 | 8 | 1 | 05-Aug-16 | 10:24:00 AM | 10:33:00 AM | FALL | 3 | #NULL! | #NULL! | 1 | #NULL! | #NULL! | Fall | 2:23:00 PM | #NULL! | #NULL! | #NULL! | | #NULL! | #NULL! |
| CS31641 | 0 | 3 | 3 | 1 | 05-Aug-16 | 10:46:00 AM | 11:39:00 AM | FEELING FAINT | 4 | #NULL! | #NULL! | 1 | #NULL! | #NULL! | Fainting | 2:03:00 PM | #NULL! | #NULL! | #NULL! | | #NULL! | #NULL! |
| CS31642 | 0 | 3 | 1 | 0 | 05-Aug-16 | 3:04:00 PM | 3:55:00 PM | unwell vomiting yellow | 3 | #NULL! | #NULL! | 1 | #NULL! | #NULL! | Vomiting - bile stained | 6:01:00 PM | #NULL! | #NULL! | #NULL! | | #NULL! | #NULL! |
| CS31644 | 0 | 3 | 8 | 1 | 05-Aug-16 | 8:17:00 PM | 10:06:00 PM | abdo pain | #NULL! | #NULL! | 1 | 1 | #NULL! | #NULL! | Gastroenteritis | 12:17:00 AM | #NULL! | #NULL! | #NULL! | | #NULL! | #NULL! |
| CS32315 | 0 | 3 | 4 | 1 | 05-Aug-16 | 9:35:00 PM | 11:24:00 PM | headaches | 3 | #NULL! | #NULL! | 1 | #NULL! | #NULL! | Headache | 2:49:00 PM | #NULL! | #NULL! | #NULL! | | #NULL! | #NULL! |
| CS32354 | 0 | 3 | 2 | 1 | 05-Aug-16 | 8:43:00 PM | 10:24:00 PM | early pregnant, abdominal pain | 3 | #NULL! | #NULL! | 1 | #NULL! | #NULL! | Abdominal pain | 12:18:00 AM | #NULL! | #NULL! | #NULL! | | #NULL! | #NULL! |
| CS32524 | 0 | 3 | 9 | 1 | 05-Aug-16 | 2:48:00 PM | 3:46:00 PM | FALL unwitnessed, unsteady (dementia) | 3 | #NULL! | #NULL! | 1 | #NULL! | #NULL! | Fall - accidental | 6:44:00 PM | #NULL! | #NULL! | #NULL! | | #NULL! | #NULL! |
| CS32671 | 0 | 3 | 6 | 0 | 05-Aug-16 | 3:10:00 PM | 4:02:00 PM | ?SEIZURE, hand was twitching (ca brain) | 3 | #NULL! | #NULL! | 0 | #NULL! | #NULL! | Partial seizure | 7:10:00 PM | #NULL! | #NULL! | #NULL! | | #NULL! | #NULL! |
| CS32693 | 0 | 3 | 8 | 1 | 05-Aug-16 | 10:20:00 PM | 11:23:00 PM | RUQ Pain 3/7 | 4 | #NULL! | 1 | 1 | #NULL! | #NULL! | Abdominal pain | 2:19:00 AM | #NULL! | #NULL! | #NULL! | | #NULL! | #NULL! |
| CS32713 | 0 | 3 | 9 | 0 | 05-Aug-16 | 4:09:00 PM | 4:37:00 PM | UNWELL(bradycardia atropine given) | 3 | #NULL! | #NULL! | 0 | #NULL! | #NULL! | Acute confusion | 8:09:00 PM | #NULL! | #NULL! | #NULL! | | #NULL! | #NULL! |
| CS32840 | 0 | 3 | 7 | 0 | 05-Aug-16 | 11:06:00 AM | 12:02:00 PM | SOB/Right sided CP (PE, pneumonia) | 3 | #NULL! | #NULL! | 1 | #NULL! | #NULL! | Pulmonary embolism | 3:07:00 PM | #NULL! | #NULL! | #NULL! | | #NULL! | #NULL! |
| CS32973 | 0 | 3 | 6 | 0 | 05-Aug-16 | 6:32:00 PM | 9:01:00 PM | Paraphimosis | 4 | #NULL! | #NULL! | 1 | #NULL! | #NULL! | Paraphimosis | 10:02:00 PM | #NULL! | #NULL! | #NULL! | | #NULL! | #NULL! |
| CS33011 | 0 | 3 | 8 | 1 | 05-Aug-16 | 6:52:00 PM | 8:55:00 PM | Suicidal/Intoxicated (etoh) | #NULL! | #NULL! | 1 | 1 | #NULL! | #NULL! | Alcohol intoxication (disorder) | 10:51:00 PM | #NULL! | #NULL! | #NULL! | | #NULL! | #NULL! |
| CS33793 | 0 | 3 | 7 | 1 | 05-Aug-16 | 2:25:00 PM | 3:14:00 PM | COLLAPSED/AP ?seizure | 3 | #NULL! | #NULL! | 1 | #NULL! | #NULL! | Small bowel obstruction | 6:25:00 PM | #NULL! | #NULL! | #NULL! | | #NULL! | #NULL! |
| CS33926 | 0 | 3 | 7 | 1 | 05-Aug-16 | 11:44:00 AM | 12:45:00 PM | painfull red eye] | 2 | #NULL! | #NULL! | 1 | #NULL! | #NULL! | Red eye | 3:05:00 PM | #NULL! | #NULL! | #NULL! | | #NULL! | #NULL! |
| CS34018 | 0 | 3 | 8 | 0 | 05-Aug-16 | 11:05:00 PM | 12:34:00 AM | ?UTI | 3 | #NULL! | #NULL! | 1 | #NULL! | #NULL! | Urinary tract infection | 2:57:00 AM | #NULL! | #NULL! | #NULL! | | #NULL! | #NULL! |
| CS34076 | 0 | 3 | 3 | 1 | 05-Aug-16 | 6:35:00 PM | 8:54:00 PM | ? chronns flare up/black stools | 4 | #NULL! | #NULL! | 1 | #NULL! | #NULL! | Crohn's disease (disorder) | 10:34:00 PM | #NULL! | #NULL! | #NULL! | | #NULL! | #NULL! |
| CS31018 | 1 | 3 | 9 | 1 | 05-Aug-16 | 6:26:00 PM | 8:19:00 PM | SOB(inpatient langley green) | 4 | #NULL! | #NULL! | 0 | 1 | #NULL! | Pneumonia | 4:57:00 AM | 1 | 1.00 | ######## | SPR | 0 | #NULL! |
| CS31643 | 1 | 3 | 5 | 0 | 05-Aug-16 | 8:03:00 PM | 10:05:00 PM | Flank Pain(renal colic) | 3 | 0 | 1 | 0 | 0 | #NULL! | Left flank pain | 11:13:00 PM | 0 | 1.00 | ######## | Consutlan | 0 | #NULL! |
| CS32456 | 1 | 3 | 7 | 0 | 05-Aug-16 | 7:27:00 PM | 9:39:00 PM | sudden onset pain L testis | 4 | 0 | #NULL! | 0 | 0 | #NULL! | O/E - testicular swelling | 11:23:00 PM | 0 | 1.00 | ######## | Consutlan | 0 | #NULL! |
| CS30038 | 0 | 3 | 7 | 0 | 06-Aug-16 | 7:45:00 PM | 9:01:00 PM | 2/7 hx CCP | 3 | #NULL! | #NULL! | 1 | #NULL! | #NULL! | Community acquired pneumonia | 9:46:00 PM | #NULL! | #NULL! | #NULL! | | #NULL! | #NULL! |
| CS30156 | 0 | 3 | 3 | 1 | 06-Aug-16 | 1:19:00 AM | 3:08:00 AM | Headache took 24xParacetamol 500mg+4 kalms | 2 | #NULL! | 1 | 1 | #NULL! | #NULL! | Paracetamol overdose | 7:39:00 AM | #NULL! | #NULL! | #NULL! | | #NULL! | #NULL! |
| CS30320 | 0 | 3 | 5 | 0 | 06-Aug-16 | 1:55:00 AM | 4:00:00 AM | Assault HI/Lft shoulder pain | 4 | #NULL! | 1 | 1 | #NULL! | #NULL! | Assault | 5:55:00 AM | #NULL! | #NULL! | #NULL! | | #NULL! | #NULL! |
| CS30351 | 0 | 3 | 3 | 1 | 06-Aug-16 | 2:02:00 AM | 4:16:00 AM | Ear Infection(flucloxacillin) | 4 | #NULL! | #NULL! | 1 | #NULL! | #NULL! | Otitis media | 5:25:00 AM | #NULL! | #NULL! | #NULL! | | #NULL! | #NULL! |
| CS30392 | 0 | 3 | 7 | 0 | 06-Aug-16 | 7:08:00 PM | 8:58:00 PM | R flank pain | 4 | #NULL! | #NULL! | 1 | #NULL! | #NULL! | Renal colic | 10:24:00 PM | #NULL! | #NULL! | #NULL! | | #NULL! | #NULL! |
| CS30398 | 0 | 3 | 3 | 0 | 06-Aug-16 | 7:00:00 PM | 8:58:00 PM | ?collapse - been drinking | 3 | #NULL! | #NULL! | 1 | #NULL! | #NULL! | Drunk | 10:43:00 PM | #NULL! | #NULL! | #NULL! | | #NULL! | #NULL! |
| CS30458 | 0 | 3 | 9 | 0 | 06-Aug-16 | 11:49:00 PM | 1:47:00 AM | chest pain | 3 | #NULL! | #NULL! | 1 | #NULL! | #NULL! | Stable angina (disorder) | 3:49:00 AM | #NULL! | #NULL! | #NULL! | | #NULL! | #NULL! |
| CS30695 | 0 | 3 | 6 | 0 | 06-Aug-16 | 9:49:00 PM | 12:02:00 AM | RTC 14hrs ago lower back pain,headache | 4 | #NULL! | #NULL! | 1 | #NULL! | #NULL! | Back pain | 12:44:00 AM | #NULL! | #NULL! | #NULL! | | #NULL! | #NULL! |
| CS30834 | 0 | 3 | 8 | 0 | 06-Aug-16 | 11:19:00 PM | 12:55:00 AM | non epileptic seizure | 3 | #NULL! | #NULL! | 1 | #NULL! | #NULL! | Chronic confusion | 10:13:00 AM | #NULL! | #NULL! | #NULL! | | #NULL! | #NULL! |
| CS31121 | 0 | 3 | 3 | 1 | 06-Aug-16 | 9:08:00 PM | 11:14:00 PM | OD/Mental health | #NULL! | #NULL! | #NULL! | 1 | #NULL! | #NULL! | Self-discharge | 12:26:00 AM | #NULL! | #NULL! | #NULL! | | #NULL! | #NULL! |
| CS31205 | 0 | 3 | 3 | 1 | 06-Aug-16 | 2:22:00 AM | 3:53:00 AM | Passing small amounts urine | #NULL! | #NULL! | 1 | 1 | #NULL! | #NULL! | Renal colic | 5:37:00 AM | #NULL! | #NULL! | #NULL! | | #NULL! | #NULL! |
| CS31252 | 0 | 3 | 2 | 1 | 06-Aug-16 | 2:29:00 AM | 5:03:00 AM | smoked cigarette.feels funny | 4 | #NULL! | 1 | 1 | #NULL! | #NULL! | Cigarette consumption | 5:42:00 AM | #NULL! | #NULL! | #NULL! | | #NULL! | #NULL! |
| CS31358 | 0 | 3 | 4 | 1 | 06-Aug-16 | 8:00:00 PM | 9:39:00 PM | FLANK PAIN, KIDNEY STONES | 4 | #NULL! | #NULL! | 1 | #NULL! | #NULL! | Flank pain (finding) | 12:00:00 AM | #NULL! | #NULL! | #NULL! | | #NULL! | #NULL! |
| CS31534 | 0 | 3 | 3 | 1 | 06-Aug-16 | 12:45:00 AM | 2:08:00 AM | Epigastric Pain | 4 | #NULL! | 1 | 1 | #NULL! | #NULL! | Gastritis | 4:39:00 AM | #NULL! | #NULL! | #NULL! | | #NULL! | #NULL! |
| CS31607 | 0 | 3 | 3 | 1 | 06-Aug-16 | 1:41:00 AM | 3:08:00 AM | Abdo/back Pain | 4 | #NULL! | #NULL! | 1 | #NULL! | #NULL! | Palpitations - fluttering | 4:37:00 AM | #NULL! | #NULL! | #NULL! | | #NULL! | #NULL! |
| CS31624 | 0 | 3 | 4 | 0 | 06-Aug-16 | 11:32:00 PM | 12:55:00 AM | ?Cellulitis/Ulcers Bilateral | 2 | #NULL! | #NULL! | 1 | #NULL! | #NULL! | Cellulitis | 3:32:00 AM | #NULL! | #NULL! | #NULL! | | #NULL! | #NULL! |

Supplementary material 2, assessment of clinical adequacy questions

Excel spread sheet sent to each reviewer to complete for all records. One column for each record – reviewer to fill in id number

| | Questions * | Insert study ID number from top of record<br>*Drop down response options followed by a cell for free text if appropriate* |
|---|---|---|
| 1 | Record of the patient's medical history | Appropriate<br>Or<br>Error or omission – but unlikely to have resulted in harm or different treatment<br>Or<br>An error or omission seen that caused a breach in normal guidelines and procedures that would have altered the patient's treatment<br>Or<br>An error or omission seen that resulted in significant probability that the patient might be harmed |
| | Free text on rationale or comment on response to item 1 | |
| 2 | Examination of the patient | Appropriate<br>Or<br>Error or omission – but unlikely to have resulted in harm or different treatment<br>Or<br>An error or omission seen that caused a breach in normal guidelines and procedures that would have altered the patient's treatment<br>Or<br>An error or omission seen that resulted in significant probability that the patient might be harmed |
| | Free text on rationale or comment on response to item 2 | |
| 3 | Request for radiography | Appropriate<br>Or<br>Error or omission – but unlikely to have resulted in harm or different treatment<br>Or<br>An error or omission seen that caused a breach in normal guidelines and procedures that would have altered the patient's treatment<br>Or<br>An error or omission seen that resulted in significant probability that the patient might be harmed |
| | Free text on rationale or comment on response to item 3 | |
| 4 | Treatment plan and decision | Appropriate<br>Or<br>Error or omission – but unlikely to have resulted in harm or different treatment<br>Or<br>An error or omission seen that caused a breach in normal guidelines and procedures that would have altered the patient's treatment<br>Or<br>An error or omission seen that resulted in significant probability that the patient might be harmed |



Halter M, *et al. BMJ Open* 2020; 10:e037557. doi: 10.1136/bmjopen-2020-037557

| | Questions * | Insert study ID number from top of record<br>*Drop down response options followed by a cell for free text if appropriate* |
|---|---|---|
| | Free text on rationale or comment on response to item 4 | |
| 5 | Treatment plan and decision reviewed by senior doctor | YES<br>Or<br>NO |
| 6 | Advice given | Appropriate<br>Or<br>Error or omission – but unlikely to have resulted in harm or different treatment<br>Or<br>An error or omission seen that caused a breach in normal guidelines and procedures that would have altered the patient's treatment<br>Or<br>An error or omission seen that resulted in significant probability that the patient might be harmed |
| | Free text on rationale or comment on response to item 6 | |
| 7 | Follow-up | Appropriate<br>Or<br>Error or omission – but unlikely to have resulted in harm or different treatment<br>Or<br>An error or omission seen that caused a breach in normal guidelines and procedures that would have altered the patient's treatment<br>Or<br>An error or omission seen that resulted in significant probability that the patient might be harmed |
| | Free text on rationale or comment on response to item 7 | |
| 8 | In your view what type of clinician attended this patient? | Doctor<br>Or<br>Physician associate<br>Or<br>Unable to decide |
| | Free text on rationale or comment on response to item 8 | |

* Review questions taken from Sakr et al study [17] comparing patients attended by advanced nurse practitioners with doctors in the ED.



Halter M, *et al. BMJ Open* 2020; 10:e037557. doi: 10.1136/bmjopen-2020-037557

Supplementary file 3: Observation guide

## OBSERVATION: AIDE MEMOIRE for researchers

Our observation aims to support answering the four study research questions but specifically to provide data on impact on organisation of services, other team members working practices and team relationships.

We wish to be able to consider this in terms of:

- Acceptability -how do they appear to be viewed or treated by others?
- Appropriateness – how are they observed in terms of safety e.g. how do they check, how are they checked upon, how are they supervised?
- Equity - who receives the PA service; do any patient groups appear to be over represented?
- Efficiency - how do they appear to contribute to this? How are issues, such as prescribing, worked around?
- Effectiveness – are the outcomes of PA care or contribution to the team observed?

We are observing context, relationships and activities.

**Conduct of the observation**

- Put up approved notices of our observation activity in the study setting places advised by the clinical team
- Provide the PA with the approved script to inform patients/ patients' representatives to gain permission for the researcher's presence. Each patient is to be asked for permission.
- Researcher to maintain an unobtrusive presence
- Record observations in the ethnographic tradition - take detailed unstructured notes, bearing in mind the importance of capturing context, relationships and activities
- Record as much as possible at the time and add as soon as possible afterwards
- Length of observation to be pre-planned but also to allow for flexibility according to the PA's wishes, the demands of the clinical setting and researcher's length of focus
- Allow the PA to see the notes at any point

**After the observation**

- Add to the notes as soon as possible where detail was not able to be captured at the time
- Maintain a reflective diary associated with the observation conduct and analytical processes.
- Discuss the observation with local research team members to promote group understanding and consistency across researchers
- Transfer data into NVivo software.



Halter M, *et al. BMJ Open* 2020; 10:e037557. doi: 10.1136/bmjopen-2020-037557

Supplementary data file 4: Tailored topic guides for the interview

## Topic guide for senior managers and clinicians

Topic areas

- Confirm the person's job role
- Ask them to describe their involvement with physician associate employment in the hospital to date
- Ask questions on the factors supporting the adoption of the employment of physician associates
- Ask questions on the factors inhibiting the employment of physician associates
- Questions on their views of physician associates' impact on (ask for examples):
    - Organisation of services
    - Patient experience and outcomes
    - Other staff
    - Costs
- Anything else they would like to say?

**Interviewer to probe** on all answers to ensure the meaning is clear (e.g. that is an interesting point , can you explain a bit more about it ) and check for understanding (e.g. so can I check I have understood you correctly )…….

**Interviewer to check** for any routine management reports or data or evaluations that the hospital team would be willing to share with the researchers.

Thank them and ask if they would like to receive updates on the study and a final summary of the findings. If so could they please give contact details which will be kept separate from the interview data.

## Topic guide for physician associate interviews

Topic areas

- Ask them to describe how long they have been a physician associate, how many posts, type and length as a physician associate
- Ask them to describe the work they undertake, with what type of medical/surgical team
- Ask about their supervising doctor and arrangements when they are not there
- Ask questions on their views of the factors supporting the adoption of the employment of physician associate in their experience



Halter M, *et al. BMJ Open* 2020; 10:e037557. doi: 10.1136/bmjopen-2020-037557

- Ask questions on their views of the factors inhibiting the employment of physician associate in their experience
- Ask how they have been received in the hospital as a new type of health professional?
- Ask how they explain to patients, family and staff – who they are and what a physician associate is

- Questions on their views of their, or other physician associates, impact on (ask for examples):
  - Organisation of services
  - Patient experience and outcomes
  - Other staff
  - Costs

- Anything else they would like to say?

**Interviewer to probe** on all answers to ensure the meaning is clear (e.g that is an interesting point , can you explain a bit more about it) and check for understanding (e.g. so can I check I have understood you correctly )…….

**Interviewer to check** for any routine management reports or data or evaluations that the hospital team would be willing to share with the researchers .

Thank them and ask if they would like to receive updates on the study and a final summary of the findings. If so could they please give contact details which will be kept separate from the interview data.

## Topic guide for all other types of professionals/managers interviews

- Confirm the person's job role
- Ask them to describe their involvement with physician associate employment in the hospital to date
- Ask questions on their views of any factors supporting the adoption of the employment of physician associates in their experience
- Ask questions on their views of any factors inhibiting the employment of physician associates in their experience
- Ask their views as to how the PAs have been received in that service/team, and probe for any explanations

- Questions on their views of physician associates' impact on (ask for examples):
  - Organisation of services
  - Boundaries between the job roles of different types of professionals e.g. with nurses
  - Patient experience and outcomes
  - Other staff
  - Costs

- Anything else they would like to say?

**Interviewer to probe** on all answers to ensure the meaning is clear (e.g that is an interesting point , can you explain a bit more about it ) and check for understanding (e.g. so can I check I have understood you correctly )…….

**Interviewer to check** for any routine management reports or data or evaluations that the hospital team would be willing to share with the researchers.

Thank them and ask if they would like to receive updates on the study and a final summary of the findings. If so could they please give contact details which will be kept separate from the interview data.

---

## Topic guide for patient interviews

<u>Topic areas</u>

- Confirm the person is/has been a patient
- Ask them to outline the type of care they have been in receipt of without giving personal medical details e.g. in patient for x days
- Confirm the patient has met the physician associate
- Explore what sort of involvement the physician associate has had with them
- Ask them how they understand the role of the physician associate in the medical/surgical team
- Ask them how they found receiving care from a physician associate
- If they were to need similar medical or surgical care, would they be content to receive similar care from a physician associate in the future as they had this time (and can



Halter M, *et al. BMJ Open* 2020; 10:e037557. doi: 10.1136/bmjopen-2020-037557

they explain why) or would they prefer someone different? And if yes, can they explain why?

- Anything else they would like to say?

**Interviewer to probe** on all answers to ensure the meaning is clear (e.g. that is an interesting point , can you explain a bit more about it) and check for understanding (e.g. so can I check I have understood you correctly).

Thank them and ask if they would like to receive updates on the study and a final summary of the findings. If so could they please give contact details which will be kept separate from the interview data.



Halter M*, et al. BMJ Open* 2020; 10:e037557. doi: 10.1136/bmjopen-2020-037557
