# Supplementary material for: Comparing physician associates and foundation year two doctors-in-training undertaking emergency medicine consultations in England: a mixed-methods study of processes and outcomes
Source: BMJ Open. 2020 Sep 1;10(9):e037557. doi: 10.1136/bmjopen-2020-037557 (PMC7467515; doi:10.1136/bmjopen-2020-037557)
Supplement: Author's manuscript [file bmjopen-2020-037557.draft_revisions.pdf]

BMJ Open is committed to open peer review. As part of this commitment we make the peer review history of every article we publish publicly available.

When an article is published we post the peer reviewers' comments and the authors' responses online. We also post the versions of the paper that were used during peer review. These are the versions that the peer review comments apply to.

The versions of the paper that follow are the versions that were submitted during the peer review process. They are not the versions of record or the final published versions. They should not be cited or distributed as the published version of this manuscript.

BMJ Open is an open access journal and the full, final, typeset and author-corrected version of record of the manuscript is available on our site with no access controls, subscription charges or pay-per-view fees (http://bmjopen.bmj.com).

If you have any questions on BMJ Open's open peer review process please email info.bmjopen@bmj.com

BMJ Open

Comparing physician associates and foundation year two doctors in training undertaking emergency medicine consultations in England: a mixed methods study of processes and outcomes

Journal:	*BMJ Open*
Manuscript ID	bmjopen-2020-037557
Article Type:	Original research
Date Submitted by the Author:	12-Feb-2020
Complete List of Authors:	Halter, Mary; St Georges University Hospital, Faculty of Health and Social Care Sciences<br>Drennan, Vari; Kingston University Faculty of Health Social Care and Education, Centre for Health & Social Care Research<br>Wang, Chao; Kingston University Faculty of Health Social Care and Education,<br>Wheeler, Carly; Imperial College Healthcare NHS Trust, Centre for Medication Safety and Service Quality<br>Gage, Heather; University of Surrey, School of Economics<br>Nice, Laura; University of Birmingham,<br>de Lusignan, Simon; University of Oxford, Nuffield Department of Primary Care Health Sciences; University of Surrey, Department of Clinical and Experimental Medicine<br>Gabe, Jonathan; Royal Holloway University of London, School of Law and Social Science<br>Brearley, Sally; Kingston University and St George's, university of London, Faculty of Health, Social Care and Education<br>Ennis, James; University of Birmingham, Institute of Clinical Sciences<br>Begg, Phil; Royal Orthopaedic Hospital, Executive<br>Parle, Jim; University of Birmingham, Institute of Clinical Sciences
Keywords:	ACCIDENT & EMERGENCY MEDICINE, Organisation of health services < HEALTH SERVICES ADMINISTRATION & MANAGEMENT, Quality in health care < HEALTH SERVICES ADMINISTRATION & MANAGEMENT

SCHOLARONE™
Manuscripts

I, the Submitting Author has the right to grant and does grant on behalf of all authors of the Work (as defined in the below author licence), an exclusive licence and/or a non-exclusive licence for contributions from authors who are: i) UK Crown employees; ii) where BMJ has agreed a CC-BY licence shall apply, and/or iii) in accordance with the terms applicable for US Federal Government officers or employees acting as part of their official duties; on a worldwide, perpetual, irrevocable, royalty-free basis to BMJ Publishing Group Ltd ("BMJ") its licensees and where the relevant Journal is co-owned by BMJ to the co-owners of the Journal, to publish the Work in this journal and any other BMJ products and to exploit all rights, as set out in our licence.

The Submitting Author accepts and understands that any supply made under these terms is made by BMJ to the Submitting Author unless you are acting as an employee on behalf of your employer or a postgraduate student of an affiliated institution which is paying any applicable article publishing charge ("APC") for Open Access articles. Where the Submitting Author wishes to make the Work available on an Open Access basis (and intends to pay the relevant APC), the terms of reuse of such Open Access shall be governed by a Creative Commons licence – details of these licences and which Creative Commons licence will apply to this Work are set out in our licence referred to above.

Other than as permitted in any relevant BMJ Author's Self Archiving Policies, I confirm this Work has not been accepted for publication elsewhere, is not being considered for publication elsewhere and does not duplicate material already published. I confirm all authors consent to publication of this Work and authorise the granting of this licence.

For peer review only

Comparing physician associates and foundation year two doctors in training undertaking emergency medicine consultations in England: a mixed methods study of processes and outcomes

Corresponding author

Mary Halter

Faculty of Health, Social Care and Education, Kingston University St George's, University of London, Cranmer Terrace, London SW17 0RE, UK. maryhalter@sgul.kingston.ac.uk 020 8725 0337

Co-authors

Vari M Drennan

Faculty of Health, Social Care and Education, Kingston University St George's, University of London, London, UK

V.Drennan@sgul.kingston.ac.uk

Chao Wang

Faculty of Health, Social Care and Education, Kingston University St George's, University of London, Cranmer Terrace, London, UK

C.Wang@sgul.kingston.ac.uk

Carly Wheeler

Centre for Medication Safety and Service Quality, Imperial College Healthcare NHS Trust, London, UK

carly.wheeler@gmail.com

Heather Gage

Surrey Health Economics Centre, Department of Clinical and Experimental Medicine, University of Surrey, Guildford, UK

h.gage@surrey.ac.uk

Laura Nice

Institute of Clinical Sciences, University of Birmingham, UK

L.Nice@bham.ac.uk

For peer review only

Simon de Lusignan
Nuffield Department of Primary Care Health Sciences, Oxford, UK
simon.delusignan@phc.ox.ac.uk

Jonathan Gabe PhD
School of Law and Social Science, Royal Holloway, University of London, Egham, UK
J.Gabe@rhul.ac.uk

Sally Brearley
Faculty of Health, Social Care and Education, Kingston University St George's, University of London, Cranmer Terrace, London, UK
S.Brearley@sgul.kingston.ac.uk

James Ennis
Institute of Clinical Sciences, University of Birmingham, UK
j.ennis@chester.ac.uk

Philip Begg
The Royal Orthopaedic Hospital Birmingham, UK
philip.begg@nhs.net

Jim Parle
Institute of Clinical Sciences, University of Birmingham, UK
j.v.parle@bham.ac.uk

Keywords

Physician Assistants; physician associates; Emergency Service, Hospital; emergency department; medical education

Word count:
Abstract 300
Main text 3630

For peer review only - http://bmjopen.bmj.com/site/about/guidelines.xhtml

Comparing physician associates and foundation year two doctors in training undertaking emergency medicine consultations in England: a mixed methods study of processes and outcomes

ABSTRACT

Objectives

To compare the contribution of physician associates to the processes and outcomes of emergency medicine consultations to that of foundation year two doctors-in-training.

Design

Mixed methods study: retrospective chart review using four months' anonymised clinical record data of all patients seen by physician associates or foundation year two doctors-in-training in 2016; review of a sub sample of 40 records for clinical adequacy; semi-structured interviews with staff and patients; observations of physician associates.

Setting

Three emergency departments in England

Participants

The records of 3197 patients attended by six physician associates (n=1129) and 22 foundation year two doctors-in-training (n=2068); 14 clinicians and managers and six patients or relatives for interview; five phyisican associates for observation.

Primary and secondary outcome measures

The primary outcome was unplanned re-attendance at the same emergency department within seven days. Secondary outcomes: consultation processes, clinical adequacy of care, and staff and patient experience.

Results

Re-attendances within seven days (n=194 [2.2%]) showed no difference between physician associates and foundation year two doctors-in-training (OR 0.87 [95% CI 0.61, 1.24], p=0.437). If seen by a physician associate, patients were more likely to undergo an x-ray investigation (OR 4.33 95% CI [3.64, 5.15)] , p<0.001) and less likely to be admitted to hospital (OR 0.75 95% CI [0.61, 0.92], p=0.006), after adjustment for patient characteristics and triage severity of condition. Clinical reviewers found almost all patients' charts clinically adequate. Physician associates were evaluated as assessing patients in a similar way to foundation year two doctors-in-training and providing

For peer review only - http://bmjopen.bmj.com/site/about/guidelines.xhtml

continuity in the team. Patients were positive about the care they had received from a physician associate, but had poor understanding of the role.

Conclusions

Physician associates in emergency departments in England treated patients with a range of conditions safely, and at a similar level to foundation year two doctors-in-training, providing clinical operational efficiencies.

For peer review only

Article summary

Strengths and limitations of this study

- This study provides a well-powered quantitative comparative analysis of the documented processes and outcomes of patient care by physician associates and foundaion year two doctors-in-training in three emergency departments in different parts of England.
- We believe this to be the first empirical study of the outcomes of care provided by UK-trained physician associates in the emergency department, and the first internationally to include interview and observation data.
- Patients' views have not been previously reported for physician associates in this setting.
- The low sensitivity of the emergency department triage system to identify conditions other than the most serious was a problem and impaired the study's ability to describe case mix fully.

The original protocol for the study

The protocol for the study is available at the funding body's website https://www.journalslibrary.nihr.ac.uk/programmes/hsdr/141926/#/

Funding

This project was funded by the National Institute for Health Research Health Services and Delivery Research Programme (project number 14/19/26). This paper presents independent research commissioned by the National Institute for Health Research (NIHR). The views and opinions expressed by authors in this publication are those of the authors and do not necessarily reflect those of the NHS, the NIHR, the Health Service and Delivery Research Programme or the Department of Health.

Competing Interests

SdeL was head of the Department of Clinical and Experimental Medicine until June 2019 at the University of Surrey, which launched a physician associate course in 2016. JP is the immediate past chair of the UK and Ireland Board for Physician Associate Education and immediate past director of the physician associate programme at the University of Birmingham. PB is honorary faculty at the University of Birmingham and has taught on the physician associate programme since 2008. JE taught part time on the University of Birmingham physician associate programme until 2020. VMD was a HS&DR Board Member in 2015.

For peer review only

What this paper adds

What is already known on this subject
Growing patient demand on emergency departments and shortages or maldistribution of doctors in many countries have led clinicians and managers to seek other workforce solutions, including the employment of non-physician clinicians such as nurse practititoners and physician assistants/associates. Observational studies from the USA and descriptive accounts from the UK found physician associates in the emergency department to be well accepted and reliable, though the USA evidence presents varying clinical outcomes. There is little research on Physician Associates from outside of the USA; limited evidence of their clinical effectiveness and no qualitative evidence of how PAs deliver care in the emergency department.
**What this study adds**
Our study suggests that physician associates and foundation year two doctors-in-training practice equally safely and appropriately in the emergency department, with no difference in re-attendance rates. Physician associates provide continuity in staff teams, although there are currently some limitations to their practice. Our study supports a role for physician associates in emergency medicine with supervision and more broadly for regulation of the profession.

For peer review only

Main text

Introduction

Health care systems internationally are challenged to ensure good patient outcomes, within financial constraints, as well as to attend to the work life of the workforce.[1] Health workforce shortages, particularly of doctors, are resulting in the development of advanced clinical practitioners or non-physician clinicians (NPCs), such as nurse practitioners (NPs) and physician assistants/associates (PAs) in many countries.[2] Numerous countries are experiencing rising patient demand for emergency services and concommittant shortages of doctors in emergency medicine.[3-7] This situation has led to the development of NPC roles in emergency departments (EDs) in many countries such as the United States (US),[8] Australia,[9] Canada,[10] and the United Kingdom (UK).[11] In the US, 25% (n=14,360) of all emergency medicine clinicians are NPCs, and 68% of these are PAs.[8]

PAs are trained in the medical model to take histories, diagnose illness, develop management plans and prescribe medications as agreed with their supervising physician. PAs have a fifty year history in the US and are a developing part of the workforce in some other countries such as Canada, the Netherlands and Germany.[12] The PA workforce is growing in the UK (where they are known as physician associates). In 2018 there was an estimated 600 qualified PAs with approximately 1000 graduating each year since then.[11] Their employment specialties include EDs,[11] where they are deployed in both the minor and the major illness or injury sections.[8]

Descriptive observations have been published concerning the positive contributions by US-trained PAs employed in EDs in the UK,[13] Australia and New Zealand,[14] and by UK-trained PAs in England.[15] Unlike in the US, PAs in these other countries cannot prescribe medicines or order ionising radiation. PAs in North American EDs are reported to be well accepted by other staff and patients, and reliable in assessing certain medical complaints and performing procedures.[16] No difference is reported between patients attended by a PA and those attended by a doctor for wound infection rates, or rate of revisit within 72 hours to a pediatric ED; but studies find less consistency in practice when analysing prescribing patterns, length of stay and wait times of physicians, PAs and NPs in the ED.[17] There is relatively little research evidence on their clinical effectiveness,[17] little quantitative evidence on outcomes from outside of the US and no qualitative evidence of how PAs deliver care in the ED. In this context our goal was to investigate the contribution of PAs to the processes and outcomes of emergency medicine consultations compared to that of foundation year two (FY2) doctors-in-training in EDs in English hospitals.

For peer review only - http://bmjopen.bmj.com/site/about/guidelines.xhtml

Methods

Study design

We conducted a pragmatic, mixed methods convergence study in which we compare and contrast and simultaneously interpret quantitative and qualitative data[18] in three EDs in England, with three components. We undertook a quantitative observational retrospective chart review of patient consultations by PAs compared with FY2 doctors-in-training; and qualitatively we directly observed PAs' practice; and we conducted semi-structured interviews with members of the staff team. Our planned prospective study of patient records with a linked patient satisfaction and outcomes survey had to be revised to a pragmatic retrospective chart review due to practicalities within the participating NHS organisations in the period of the study.

Population and sampling

Three consultant-led, 24 hour EDs with full resuscitation facilities ('type one') participated. Two EDs had annual attendances in the range of 100,000 – 120,000 adult and pediatric patients and the third in the range of 170,000 -190,000. One was an university hospital; two were district general hospitals. The hospitals had been recruited as part of a larger study investigating the work and contribution of PAs between 2016 and 2017.[19] We selected foundation year two doctors as the comparator for PAs, as PAs are offered as part of a solution to junior medical workforce shortages.[7]

Selection of participants, measurements and outcomes

Our primary outcome was unplanned re-attendance at the same ED within seven days - one of the NHS clinical quality indicators for EDs in England.[20] Our secondary outcomes were: consultation processes (length of time in the ED, use of x-ray, prescriptions and referrals); clinical adequacy of care, referrals and planned follow up; and patient experience.

Chart review

For a 16-week period (the standard duration of ED placement for FY2 doctors-in-training in the UK), we obtained pseudo-anonymised, routinely-collected electronic records of all patients attended by a PA or FY2 doctor-in-training, provided in Microsoft Excel by the hospital information teams in each trust, using queries based on staff job role , dates and requested data items. Hospital staff extracted additional data items (supplementary material 1) – age, sex, acuity (as categorised by the Manchester triage score [21]), x-ray orders, diagnosis, prescription issued, admission, area treated, overall time in

For peer review only - http://bmjopen.bmj.com/site/about/guidelines.xhtml

the ED (from check in to discharge, in minutes), and re-consultation within seven days (the primary outcome). No data linkage was required. The researchers did not have access to the original data set and further data cleaning could not be performed.

We calculated a sample size for the primary outcome using the national average of 7.4% (range 2.4% to 21.7%) for unplanned re-attendance at the same ED in England within seven days for all patients; and 18.3% (the highest of two rates for nurse practitioners substituting for physicians).[22,23] Aiming to find a relative difference of 50%, in a non-inferiority hypothesis, we required 284 patients in each group (calculation from Stata v11.1 software) to compare 18.3% to 27.4% unplanned re-consultations within seven days, with conventional 80% power at 5% significance. We included an extra 20 to allow for adjustment for case mix, requiring a minimum of 304 patients in total in each group to achieve the said power.

Two of the participating EDs also agreed to take part in the analysis of clinical adequacy of documented care in every tenth case from the chart review sample (n=40), with equal numbers of cases seen by PA and FY2 doctors-in-training, and using the full anonymised clinical record. We recruited two specialty registrars (doctors in their sixth year of emergency medicine training), one PA lecturer with 20 years ED experience, and one emergency medicine consultant (with 17 years experience at consultant level) from outside the three study hospitals to review these records. All four clinicians independently recorded their judgement as to the clinical adequacy of care for each record using the categories of past medical history, examination, request for radiography, treatment plan and decision, advice given and follow up. Their assessments were blinded to the type of professional attending the patient and to each other's assessment, using a proforma (supplementary material 2) based on published studies.[22,23] As the senior clinician, we accepted the decision of the consultant in cases of disagreement.

Observation

This element drew on the ethnographic tradition used in many health service research studies.[24] We invited all PAs working in the ED in our three study hospitals to participate (n=6). Five PAs volunteered and gave written informed consent to be observed. One of three researchers (CWh, LN, MH) observed each PA for two or three pre-arranged sessions, of varying lengths, on weekdays in periods between 08.00 and 22.00, following a broad guide (supplementary material 3). Researchers made notes on context, relationships and activities following this guide.We judged data saturation to have been reached with individual PAs when the processes of care observed did not differ significantly from previous observations. During the observation period, PAs asked for patient assent to the researcher's presence. Researchers reflected on the observations, discussing them in pairs.

For peer review only - http://bmjopen.bmj.com/site/about/guidelines.xhtml

Interview

Semi structured interviews [25] were undertaken with a purposive sample of managerial, medical and nursing ED staff as well as patients and/or their relative who were being seen by a PA in the ED. We used tailored topic guides (supplementary material 4) to explore interviewees' perceptions of the PA role and its impact on service organization, role boundaries, patient experience, patient outcomes, and activities and attitudes of other staff. We digitally recorded interviews or took notes if the participant preferred. Recordings were transcribed verbatim and anonymised.

Analysis

Chart review: The characteristics of patients treated by PAs and FY2 doctors-in-training were compared using chi-squared tests. We carried out a logistic regression to examine whether the primary and binary secondary outcomes differed between PAs and FY2 doctors-in-training, while adjusting for confounding factors - patient age, sex and triage score. We report odds ratios, their confidence intervals (CI), and two-tailed p values. For length of stay, a linear regression was used for data transformed to logarithm scale to improve normality and reflect the fact that the value of length of stay is positive. Robust standard errors were used in the presence of heteroscedasticity. To account for unobserved heterogeneity, the unobserved component is modelled as a latent variable in a latent class linear model. The assessment of clinical adequacy is reported using descriptive statistics, and sensitivity and specificity of the judgment of whether the record was that of a PA or FY2 doctor-in-training.

Qualitative: Our methods for the analysis of observation data drew on methods to identify ethnographic vignettes.[26] We employed thematic analysis[27] of all-specialty interview data for the wider study. Both are described in full elsewhere.[19] For the subsequent specialty-specific analysis we re-read all ED observation data and interview transcripts to identify all data related to the primary and secondary outcomes, and which both confirmed or disconfirmed findings.

Mixed methods: Following the separate quantitative and qualitative analyses, we (MH and VMD, in consultation with all authors) merged [18] the quantitative and qualitative datasets by presenting the quantitative results by study outcomes and following these with qualitative data findings (themes and/or excerpts or quotes) that confirmed or disconfirmed the quantitative results.

Ethical approval

For peer review only - http://bmjopen.bmj.com/site/about/guidelines.xhtml

We gained approval from the NHS Health Research Authority London-Central Research Ethics Committee (15/LO/1339).

Results

Characteristics of chart review subjects

In the 16 week period studied, 8,816 patients seen by PAs or FY2 doctors-in-training were identified, of which 3197 records including the primary outcome were provided to the research team; 1129 had been seen by six PAs and 2068 by 22 FY2 doctors-in-training. Characteristics of the patients are shown in Table 1. PAs saw a lower proportion of patients categorised on triage into the urgent category than FY2 doctors-in-training.

In interview, the type of patient seen, patient throughput and role of PAs and FY2 doctors-in-training were described as similar:

They're pretty much equal to .....a senior FY2 doctor in training level. As a consultant we feel comfort because we know [PA name 1] can work in majors, she can clear [majors] pretty much...... And [PA name 2],... can clear paeds minors... Participant 150 Emergency Medicine consultant

However more than one participant tentatively suggested that PAs saw the less acutely unwell patients.:

So my understanding is like they're equivalent to, I would put it like a certain level of like a junior physician.....I wouldn't say they would be at registrar level.....I'd put them somewhere in between. You know a...lot better than like a newly qualified physician because they've got the skills and stuff, so in that gap of what I would say equivalent to maybe like a second to four years post qualified doctor.. Participant 144, Registrar

Characteristics of interview and observation participants

Staff interviewed included four PAs, two managers, five nurses and three senior doctors; six patients and/or their relatives were also interviewed, spread across the three sites. We observed four PAs, at three sites; we do not report further demographic details due to concerns about anonymity in a small population.

For peer review only - http://bmjopen.bmj.com/site/about/guidelines.xhtml

Table 1 Characteristics of chart review sample

Characteristic	PA (N = 2890) N (%)	FY2 doctor (N = 5926) N (%)	Total (N = 8816) N (%)	p-value
**Age band**				
0-20	375 (13.0%)	581 (9.8%)	956 (10.8%)	<0.001
21-40	611 (21.1%)	1425 (24.0%)	2036 (23.1%)	
41-60	637 (22.0%)	1299 (21.9%)	1936 (22.0%)	
61-80	699 (24.2%)	1448 (24.4%)	2147 (24.4%)	
81 and over	501 (17.3%)	1101 (18.6%)	1602 (18.2%)	
Unknown	67 (2.3%)	72 (1.2%)	139 (1.6%)	
**Sex**				
Male	1342 (46.4%)	2723 (46.0%)	4065 (46.1%)	0.672
Female	1548 (53.6%)	3202 (54.0%)	4750 (53.9%)	
Unknown	0 (0.0%)	1 (0.0%)	1 (0.0%)	
**Manchester triage score**				
1 Immediate	10 (0.3%)	3 (0.1%)	13 (0.1%)	<0.001
2 Very urgent	163 (5.6%)	565 (9.5%)	728 (8.3%)	
3 Urgent	769 (26.6%)	2842 (48.0%)	3611 (41.0%)	
4 Standard	811 (28.1%)	1681 (28.4%)	2492 (28.3%)	
5 Non Urgent	5 (0.2%)	12 (0.2%)	17 (0.2%)	
Unknown	1132 (39.2%)	823 (13.9%)	1955 (22.2%)	
**ED area treated in**				
Minor	386 (13.4%)	258 (4.4%)	644 (7.3%)	<0.001
Major	1757 (60.8%)	3110 (52.5%)	4867 (55.2%)	
Resuscitation	2 (0.1%)	4 (0.1%)	6 (0.1%)	
Paediatrics	181 (6.3%)	174 (2.9%)	355 (4.0%)	
Clinical decision unit or primary care	21 (0.7%)	20 (0.3%)	41 (0.5%)	
Unknown	543 (18.8%)	2360 (39.8%)	2903 (32.9%)	

For peer review only - http://bmjopen.bmj.com/site/about/guidelines.xhtml

The primary outcome: rate of return to the ED within seven days

Re-attendance within seven days was found following 2.2% (N = 194) of the 3197 index visits for which these data were available. The high rate of unknown data is accounted by one site where these data were not captured in the electronic dataset and were only retrieved manually for a random sample for the purposes of this study. After adjustment for confounding, no statistically significant difference was found for cases seen by PAs or FY2 doctors-in-training; see Table 2.

Table 2: Re-attendance at the same ED within seven days

**Re-attendance at the same ED within seven days**	PA (N = 2890)	FY2 doctor (N = 5926)	Total (N = 8816)	Unadjusted odds ratio (95% CI) and p-value (PA relative to FY2 doctor-in-training) in rate of re-attendance	Adjusted odds ratio (95% CI) and p-value (PA relative to FY2 doctor-in-training) in rate of re-attendancee†
No	1066 (36.9%)	1937 (32.7%)	3003 (34.1%)	0.87 (0.64, 1.19) p=0.393	0.87 (0.61, 1.24) p=0.437
Yes	63 (2.2%)	131 (2.2%)	194 (2.2%)		
Unknown	1761 (60.9%)	3858 (65.1%)	5619 (63.7%)		

†Adjustment made for triage score (as a measure of acuity), age band, sex, admission, x-ray and site

Secondary outcome: consultation processes

No differences were found between patients attended by PAs or by FY2 doctors-in-training in: whether prescriptions were given, or a discharge summary was completed. However, patients seen by a PA were more likely to have an x-ray performed in the ED (Table 3), less likely to be admitted to hospital, and to have a shorter length of stay in the ED (by 35 minutes), after adjustment for age, sex, acuity, whether admitted, x-ray taken, and site, although no account was able to be taken of the staffing level.

We observed PAs being the first member of the medical team to carry out assessment of patients following triage to either the major, minor or paediatric areas of the ED. We noted that PAs saw patients independently, following a medical history taking and examination model, before reporting in person to the senior ED physician in the same way as nurse practitioners and FY2 doctors-in-training do.

For peer review only - http://bmjopen.bmj.com/site/about/guidelines.xhtml

For peer review only

Table 3 Clinical process measures

Clinical process measure	PA (N = 2890)	FY2 doctor (N = 5926)	Total (N = 8816)	Unadjusted odds ratio (95% CI) and p-value (PA relative to FY2 doctor-in-training) in rate of re-attendance	Adjusted odds ratio (95% CI) and p-value (PA relative to FY2 doctor-in-training) in rate of re-attendancee†
**X-ray investigations performed**					
No	558 (19.3%)	1702 (28.7%)	2260 (25.6%)	4.77 (4.05, 5.61) p<0.001	4.33 (3.64, 5.15) p<0.001
Yes	572 (19.8%)	366 (6.2%)	938 (10.6%)		
Unknown	1760 (60.9%)	3858 (65.1%)	5618 (63.7%)		
**Prescriptions given in the ED**					
No	173 (6.0%)	158 (2.7%)	331 (3.8%)	0.79 (0.57, 1.09) p=0.147	0.75 (0.5, 1.13) p=0.173
Yes	126 (4.4%)	146 (2.5%)	272 (3.1%)		
Unknown	2591 (89.7%)	5622 (94.9%)	8213 (93.2%)		
**Admitted as an inpatient from the ED**					
No	883 (30.6%)	1436 (24.2%)	2319 (26.3%)	0.65 (0.55, 0.77) p<0.001	0.75 (0.61, 0.92) p=0.006
Yes	245 (8.5%)	614 (10.4%)	859 (9.7%)		
Unknown	1762 (61.0%)	3876 (65.4%)	5638 (64.0%)		
**Discharge summary completed**					
No	86 (3.0%)	71 (1.2%)	157 (1.8%)	0.72 (0.48, 1.08) p=0.109	1.57 (0.93, 2.66) p=0.09
Yes	117 (4.0%)	134 (2.3%)	251 (2.8%)		
Unknown	2687 (93.0%)	5721 (96.5%)	8408 (95.4%)		

†Adjustment made for MTS (as a measure of acuity), age band, sex, and site

For peer review only

PAs were differentiated from FY2 doctors-in-training by many of our interviewees for not being able to prescribe medications or order tests utilising ionising radiation. Some participants considered this to have a detrimental impact on PAs and patients:

[prescribing] would make a massive difference for them as well and [for] patients because at the end of the day they're having to wait for the PAs to go talk through [with] the physicians what's going on and then probably see somebody else. Participant 118 Nurse practitioner

For peer review only - http://bmjopen.bmj.com/site/about/guidelines.xhtml

However, PAs were observed taking on several roles in relation to prescriptions and x-ray orders, for example suggesting medications to or charting the medication for a senior doctor to sign off:

So when one of my PAs comes to me and says "This patient has a temperature of 38, they're coughing up horrible green sputum and they're tachycardic and I listened to their chest and they've got crackles at the left base, can we order a chest x-ray and prescribe sepsis drugs for, you know, pneumonia?" I say "Yes" and I sign it. With probably more confidence at this stage having had [number] PAs here for a year than I would with a junior physician in training on day two. And the irony of that is of course, the junior physician in training doesn't need to come and ask me, technically, they can prescribe themselves. Participant 21 Emergency Medicine consultant

PAs were also observed making referrals to medical and surgical teams outside of the ED, completing discharge summary information, and carrying out procedures, most commonly cannulation, phlebotomy and suturing.

Secondary outcome: clinical adequacy

Our reviewers found the chart documentation to have been appropriate in 37/40 cases for each of the key consultation components (Table 4), with no errors or omissions that resulted in significant probability that the patient might be harmed. In the three records (two of FY2 doctors-in-training and one of a PA) judged as having errors or omissions at the level of a breach in normal guidelines and procedures that would have altered the patient's treatment, all reviewers agreed that a senior doctor review had occurred in one case; this was unclear in the other cases. Our observation data suggest that such a senior review was undertaken for all assessment and clinical decision making in the 'majors' sections of the ED, but that 'minors' care was often completed independently.

Our reviewers were 40% sensitive, 46% specific on judging the clinician type: 68% (13/19) of the PA records were thought to be of a FY2 doctor-in-training and 60% (9/15) vice versa (kappa score for inter-rater agreement 0.15).

For peer review only

Table 4: Chart reviewers' assessments of clinical adequacy

**PA or FY2 consultation record**		**Judgment of appropriateness**																							
		**Past medical history**				**Examination**				**Request for radiography***				**Treatment plan and decision**				**Advice given**				**Follow up**			
		Appropriate	Error or omission			Appropriate	Error or omission			Appropriate	Error or omission			Appropriate	Error or omission			Appropriate	Error or omission			Appropriate	Error or omission		
			Harm unlikely	Altered treatment	Harm		Harm unlikely	Altered treatment	Harm		Harm unlikely	Altered treatment	Harm		Harm unlikely	Altered treatment	Harm		Harm unlikely	Altered treatment	Harm		Harm unlikely	Altered treatment	Harm
FY2	n	14	6	0	0	15	5	0	0	9	0	1	0	14	5	1	0	4	1	1	0	16	3	0	0
	%	70	37	0	0	75	25	0	0	45	0	5	0	70	25	5	0	20	5	5	0	80	15	0	0
PA	n	13	5	1	0	11	7	1	0	9	3	0	0	13	5	1	0	3	1	1	0	13	4	1	0
	%	65	25	5	0	55	35	5	0	45	15	0	0	65	25	5	0	15	5	5	0	65	20	5	0
Not rated*	n	1				1				18				1				29				3			
	%	2.5				2.5				45				2.5				73				7.5			
Total	n	27	11	1	0	26	12	1	0	18	3	1	0	27	10	2	0	0	7	2	2	29	7	1	0
	%	68	28	3	0	65	30	3	0	45	8	3	0	66	25	5	0	0	18	5	5	73	18	3	0
Agreement (Kappa)		0.01				0.15				0.26				0.15				-0.03				0.30			

*Missing rating or rated as 'not applicable' if no request for radiography was made or no advice given

For peer review only

Interviewees also presented other aspects related to clinical adequacy, particularly the PAs' stability in the team. The clinicians' familiarity with the longer standing team member PA/s - in contrast to FY2 doctors-in-training on rotation - was raised repeatedly:

> *If there was a junior physician over here, and he said oh, what do you think of this wound, which they do ask us. And I say yeah, it needs suturing. I then have to say, but can you suture or do you want me to suture it?.....Because I don't know, and some will say oh no, I can't.....I've never sutured before, and some will say oh yeah, that's fine, I'll suture it.....Whereas I know with PAs they'll suture their own. Because I know that they've got that skill set.* Participant 177 Advanced nurse practitioner

Secondary outcome: patient experience

Patients were positive about the care they had received from the PA, but had not understood what the PA role meant, with two participants believing they had been seen by a doctor and another unsure in the context of multiple ED staff:

> *I presumed he was a fully-qualified physician, yes his approach and everything was absolutely 100%.* Participant 120 Patient

Most of our patient participants were receptive to the role on the grounds that it might speed up care, although they were not without concern for the difference in training from a doctor and the diminishment of a senior medical workforce:

> *It's good to have another person, another opinion…but would it not perhaps be better to have another doctor?* Participant 083 Patient's relative

Discussion

Summary of findings

The study presents evidence from three English EDs and has demonstrated no difference in safety or appropriateness between PAs and FY2 doctors-in-training. We report no difference in re-attendance rates. Those patients seen by a PA (within PA working hours 08-22.00) had a shorter average length of stay in the ED than those seen by doctors-in-training (24 hour working period). Our review of clinical adequacy found few errors and no difference between PAs and FY2 doctors-in-training. Patients appeared relatively unconcerned with the title of the clinician treating them and thought they

For peer review only - http://bmjopen.bmj.com/site/about/guidelines.xhtml

had been treated by a doctor; however they were keen to know that the employment of PAs would not represent a widespread substitution for doctors in the ED.

How this study is similar or different from prior studies

We believe this to be the first empirical study of the outcomes of care provided by UK-trained PAs in the ED, and the first internationally to include interview and observation data. Additonally, patients' views do not appear to have been previously gathered at the time of the visit (and qualitaitvely), although there have been previous questionnaire studies in the USA of patient satisfaction, administered after the visit.[28,29,30]

We reported few differences in the the practice and processes of care – other than prescribing (which PAs currently cannot do independently in the UK) – between PAs and doctors in their second foundation year of training. Our finding of no difference in the primary outcome (ED reattendance rate within 28 days) for patients of PAs and FY2 doctors-in-training is consistent with the comparisons of nurse-qualified NPCs and FY2 doctors-in-training on which we based our study design [22,23] and other PA literature from the USA.[16] It should be noted that for patients in the majors section of ED, all assessment and treatment plans by FY2 doctors-in-training and NPCs were reviewed and agreed by a senior clinician. Our participants commented frequently on the transient nature of FY2 doctors-in-training, whose rotation in the ED only last four months. In contrast, PAs remained long-term and provided continuity in the team. Their accumulated knowledge of the policies and practices (clinical and otherwise) of the department, the consultants and the hospital was reported to enable operational efficiencies. Simlar observations about PAs providing continuity within the medical/surgical team have been made in North America and the Netherlands[31-33] and also for other NPCs.[34]

This study's strengths lie in its mixed-methods approach to the study of PAs in the ED, allowing a number of angles on their contribution, compared to that of FY2 doctors-in-training to be considered. We were able to carry out a well-powered quantitative comparative analysis of the documented processes and outcomes of patient care by PAs and FY2 doctors-in-training in three EDs in different parts of the country, and to gather qualitative data on PAs 'in practice'. The qualitative component of our mixed methods approach enabled contextual explanations of the quantitative analysis.

Our study however has several limitations. Our comparison of PAs and doctors working in all areas of the ED introduced the potential for PAs and FY2 doctors-in-training to be attending to patients of different acuity and complexity. We sought to mitigate this by using three different EDs, taking a sample across a 16 week period at all times of day and night (although the FY2 doctors-in-training

For peer review only - http://bmjopen.bmj.com/site/about/guidelines.xhtml

worked over the 24 hour period when staff:patient ratios may have fluctuated). We also made statistical adjustments that included triage category. The low sensitivity of most ED triage systems to identification of conditions other than the most serious, however, is a drawback.[35] The higher number of x-rays undertaken by PAs than by FY2 doctors-in-training may reflect a greater tendency for PAs to be allocated to minor injury cases

The level of missing data for some variables in the routinely collected data, and not having data from which to take into account whether PA reduced the staff: patient ratio (or fully replaced FY2 doctors-in-training) is a further limitation and needs to be borne in mind in the comparisons we present. Likewise, . our observation data illustrated care is predominently delivered by teams which creates difficulties in attributing outcomes or processes to individual staff, and compromised our ability to undertake an economic evaluation.

Implications for policy and practice

PAs in the ED are acceptable to patients and can help to relieve staffing pressures and improve efficiency in the delivery of care. They are able to treat patients safely with a range of conditions and FY2 doctors-in-training deliver similar care to that provided by doctors in their second year of training. Deployment of PAs within ED teams is a potential solution to the situation of growing patient demand and predicted shortage of junior doctors in the British NHS.[7] An alternative, which is to hire locum doctors, comes at a higher costs and loss of team continuity, and has potential implications for patient safety. Moves to regulate the PA profession were started in 2019 by the General Medical Council.[36]

The findings of this study support employment of appropriately trained, supervised PAs with professional registration in ED teams. Further research is needed to investigate fully the impacts we have observed, particularly the cost effectiveness.

For peer review only - http://bmjopen.bmj.com/site/about/guidelines.xhtml

References

1. Bodenheimer, T. and Sinsky, C., 2014. From triple to quadruple aim: care of the patient requires care of the provider. The Annals of Family Medicine, 12(6), pp.573-576.
2. World Health Organization. Task Shifting: Rational Redistribution Of Tasks Among Health Workforce Teams – Global Recommendations and Guidelines. Geneva: World Health Organization; 2008. URL: www.who.int/healthsystems/task_shifting/en (last accessed 8-11-2019)
3. Reiter M, Wen LS, Allen BW. The Emergency Medicine Workforce: Profile and Projections. J Emerg Med. 2016 Apr;50(4):690-3. doi: 10.1016/j.jemermed.2015.09.022. Epub 2016
4. Takagi, K. and Tagami, T., 2019. Work-style reform of emergency physicians: the Japanese experience. European Journal of Emergency Medicine, 26(6), pp.398-399.
5. Suriyawongpaisal P, Atiksawedparit P, Srithamrongsawad S, Thongtan T. Assessment of nationwide emergency systems in Thailand, a middle-income country setting with UHC. Int J Health Plann Manage. 2019 Apr;34(2):e1346-e1355. doi: 10.1002/hpm.2783. Epub 2019 Apr 4.
6. Sinclair D, Abu-Laban RB, Toth P, LeBlanc C, Eisener-Parsche P, Frank JR, Holroyd B.Emergency Medicine Training and Practice in Canada: Celebrating the Past & Evolving for the Future. Collaborative Working Group on the Future of Emergency Medicine in Canada CJEM. 2017 Jul;19(S2):S1-S8. doi: 10.1017/cem.2017.372.
7. The Royal College of Emergency Medicine and the NHS. Securing the future workforce for emergency departments in England. October 2017 https://improvement.nhs.uk/documents/1826/Emergency_department_workforce_plan_-_111017_Final.3.pdf Accessed July 31, 2019.
8. Hall MK, Burns K, Carius M et al. State of the National Emergency Department Workforce: Who Provides Care Where? *Ann Emerg Med*. 2018;72:302-307.
9. Gardner G, Gardner A, Middleton S et al. Mapping workforce configuration and operational models in Australian emergency departments: a national survey. *Austr Health Rev*. 2018;42:340-7.
10. Thrasher C, Purc-Stephenson R.J. Patient satisfaction with nurse practitioner care in emergency departments in Canada. *Am Acad Nurse Pract*. 2008;20:231-7.
11. Ritsema T. The Faculty of Physician Associates Census Results 2018. Faculty of Physician Associates at the Royal College of Physicians. http://www.fparcp.co.uk/about-fpa/fpa-census Updated date unknown. Accessed September 10, 2019.
12. Cawley JF, Hooker RS. Determinants of the physician assistant/associate concept in global health systems. *International Journal of Healthcare*. 2018;4:50-60.
13. Farmer J, Currie M, Hyman J et. al. Evaluation of physician assistants in National Health Service Scotland. *Scott Med J*. 2011;56:130-4.

For peer review only - http://bmjopen.bmj.com/site/about/guidelines.xhtml

14. Close B, Zolcinski R. Physician assistants in Australasian emergency departments. *Emerg Med Australas*. 2016;28:475-477.
15. Howie N. A new kid on the block: the role of physician associates. Clinical Medicine; 2015; 14, 6: 691–4 DOI: 10.7861/clinmedicine.15-3-309a ·
16. Doan Q, Sabhaney V, Kissoon N, et al. A systematic review: The role and impact of the physician assistant in the emergency department. *Emerg Med Australas*. 2011;23:7-15.
17. Halter M, Wheeler C, Pelone F, et al. Contribution of physician assistants/associates to secondary care: a systematic review. BMJ Open. 2018 8:e019573. doi: 10.1136/bmjopen-2017-019573.
18. Creswell JW, Clark VL. Designing and Conducting Mixed Methods Research. Thousand Oaks, CA: Sage Publications Ltd; 2007.
19. Drennan VM, Halter M, Wheeler C, et al. What is the contribution of physician associates in hospital care in England? A mixed methods, multiple case study *BMJ Open* 2019;9 e027012.
20. NHS Digital Provisional Accident and Emergency Quality Indicators - England, by provider for April 2018. Accessed 2-8-2018 https://digital.nhs.uk/data-and-information/publications/statistical/accident-and-emergency-quality-indicators/april-2018-by-provider
21. Mackway Jones K, Marsden J, Windle J. Emergency Triage: Manchester Triage Group, Third Edition . 2013. London: John Wiley & Sons.
22. Sakr M, Angus J, Perrin J, Nixon C, Nicholl J, Wardrope J. Care of minor injuries by emergency nurse practitioners or junior doctors: a randomised controlled trial. *Lancet* 1999;354:1321-6.
23. Cooper MA, Lindsay GM, Kinn S, Swann IJ. Evaluating Emergency Nurse Practitioner services: a randomized controlled trial. *J Adv Nurs*. 2002.40:721-30.
24. Pope C, Mays N. Observational Methods. In Pope C, Mays N, editors. Qualitative Research in Health Care. 4th edn. Chichester: John Wiley & Sons; 2008. pp. 32–42.
25. Kvale S. Doing Interviews. London: Sage Publications; 2007. https://doi.org/10.4135/9781849208963
26. McCann, L, Granter E, Hyde P, Hassard, J. Still Blue-Collar after all these Years? An Ethnography of the Professionalization of Emergency Ambulance Work. *Journal of Management Studies* 2013;50:750–776.
27. Boyatzis RE. *Transforming qualitative information: Thematic analysis and code development.* Thousand Oaks, Calif: Sage;1998.
28. Maxfield RG. Use of physician's assistants in a general surgical practice. The American Journal of Surgery. 1976;131:504–508. doi: 10.1016/0002-9610(76)90165-3.
29. Rodi SW, Grau MV, Orsini CM. Evaluation of a fast track unit: alignment of resources and demand results in improved satisfaction and decreased length of stay for emergency department patients. Quality Management in Healthcare. 2006;15:163–170. doi: 10.1097/00019514-200607000-00006.

For peer review only - http://bmjopen.bmj.com/site/about/guidelines.xhtml

30. Berg GM, Crowe RE, Nyberg S, Burdsal C. Trauma patient satisfaction with physician assistants: testing a structural equation model. Journal of the American Academy of PAs. 2012;25(5):42–43.
31. Fréchette D, Shrichand A. Insights into the physician assistant profession in Canada. *JAAPA* 2016;29:35–9.
32. Kartha A, Restuccia JD, Burgess JF, Benzer J, Glasgow J, Hockenberry J, et al. Nurse practitioner and physician assistant scope of practice in 118 acute care hospitals. *J Hosp Med* 2014;9:615–20.
33. Timmermans MJ, van Vught AJ, Maassen IT, Draaijer L, Hoofwijk AG, Spanier M, et al. Determinants of the sustained employment of physician assistants in hospitals: a qualitative study. *BMJ Open* 2016;6:e011949.
34. Williams K. Advanced practitioners in emergency care: a literature review. *Emergency Nurse* 2017;25:36-41.
35. Hinson JS, Martinez DA, Cabral S, et al. Triage Performance in Emergency Medicine: A Systematic Review. *Annals Emerg Med*; 2018;S0196-0644(18)31282-4.
36. General Medical Council. GMC to regulate two new associates roles. Press release 18-July-2019. https://www.gmc-uk.org/news/news-archive/gmc-to-regulate-two-new-associates-roles---pas-and-aas Accessed July 2019.

review only

For peer review only - http://bmjopen.bmj.com/site/about/guidelines.xhtml

Supplementary material

Supplementary material 1: Extract of the chart review dataset

Supplementary material 2: Proforma for the assessment of clinical adequacy

Supplementary material 3: Observation guide

Supplementary material 4: Tailored topic guides for the interview

Author Statement

VMD (PhD, health policy and service delivery research), MH (PhD, health services research), JP (MD, general practice and clinical education), HG (PhD, health economics), SdeL (MD[Res], general practice and information science), JG (PhD, medical sociology), and PB (PhD, audiology and strategic management) conceived and designed the study and obtained research funding. VMD, MH, JP supervised the conduct of the study and data collection. VMD, MH, JP undertook recruitment of participating centers and managed the data, including quality. CWa (PhD, statistics) undertook the statistical analysis; CW (PhD, health services research), LN (PhD,health services research), MH, JE (MSc, physician associate and education) and VMD undertook qualitative data collection and thematic analysis and HG considered the economic aspects. MH drafted the manuscript, and all authors contributed substantially to its revision. VMD takes responsibility for the paper as a whole. All authors attest to meeting the four ICMJE.org authorship criteria: (1) Substantial contributions to the conception or design of the work; or the acquisition, analysis, or interpretation of data for the work; AND (2) Drafting the work or revising it critically for important intellectual content; AND (3) Final approval of the version to be published; AND (4) Agreement to be accountable for all aspects of the work in ensuring that questions related to the accuracy or integrity of any part of the work are appropriately investigated and resolved.

The Patient and Public Involvement statement

The patient and public voice was important to this study and informed the design, conduct, analsyis, interpretation and final reporting. We brought the views of our public and patient representative forum for a previous study on physician associates into the research questions and design of the study. These were views such as how do patients understand this new role. Sally Brearley, as a public voice representative, was a co-applicant and member of the research team. The study advisory group had two public voice members who were reimbursed for their time, following NIHR INVOLVE guidance. Two patient and public voice groups were formed: one in London and the other in the West Midlands and members reimbursed as per NIHR Involve guidelines. The patient and public voice groups informed the design of the research tools such as topic guides and participant information sheets,

For peer review only - http://bmjopen.bmj.com/site/about/guidelines.xhtml

developed coding frameworks and analysed interview transcripts , and participated in the overall synthesis of findings. Sally Brearley continues to be involved in the dissemination of the study.

Acknowledgements

Our thanks to all those clinicians, administrative and information staff in the participating centers who assisted the study at a time of heightened workload within the emergency services in the National Health Service in England. Our thanks also to Robert Grant who provided statistical advice in the design of the study and obtained research funding but left the research team before the data were obtained for analysis.

Data statement

No additional data are available.

For peer review only

Supplementary file 1: Extract of the chart review dataset (first 50 cases, according to date of attendance)

study_id	sample_case	site_numl	age_formatted	sex_formatted	arrival_date	arrival_time	seen_time	presenting_complaint	mts	ews	ed_strean	professioi	xrays_forr	pxn_form	diagnosis	discharge_time	destination_grc	discharge_	discharge_	discharge_	reattend_	reattend_date
CS30115	0	3	5	0	05-Aug-16	9:10:00 PM	10:56:00 PM	Flank Pain(renal colic 3yrs ago)	#NULL!	#NULL!	1	1	#NULL!	#NULL!	Renal colic	2:07:00 AM	#NULL!	#NULL!	#NULL!		#NULL!	#NULL!
CS30197	0	3	6	1	05-Aug-16	7:54:00 PM	10:01:00 PM	Abdo/Back Pain 5/7	3	#NULL!	#NULL!	1	#NULL!	#NULL!	Generally unwell	1:40:00 AM	#NULL!	#NULL!	#NULL!		#NULL!	#NULL!
CS30415	0	3	4	1	05-Aug-16	4:51:00 PM	7:05:00 PM	21/40 ?DVT on clexane	3	#NULL!	#NULL!	1	#NULL!	#NULL!	Acute coronary syndrome	8:50:00 PM	#NULL!	#NULL!	#NULL!		#NULL!	#NULL!
CS30514	0	3	5	1	05-Aug-16	1:08:00 PM	2:15:00 PM	swelling to arm/hand ?clot	3	#NULL!	#NULL!	1	#NULL!	#NULL!	Deep venous thrombosis	5:07:00 PM	#NULL!	#NULL!	#NULL!		#NULL!	#NULL!
CS30726	0	3	3	0	05-Aug-16	11:45:00 PM	1:12:00 AM	HEADACHE	2	#NULL!	1	1	#NULL!	#NULL!	Unknown problem	1:58:00 AM	#NULL!	#NULL!	#NULL!		#NULL!	#NULL!
CS30962	0	3	4	0	05-Aug-16	4:26:00 PM	6:59:00 PM	Back Pain(mr maurice bulging disc)	3	#NULL!	#NULL!	0	#NULL!	#NULL!	Chronic back pain	8:20:00 PM	#NULL!	#NULL!	#NULL!		#NULL!	#NULL!
CS30993	0	3	4	1	05-Aug-16	6:57:00 AM	8:04:00 AM	Epigastric Pain 13months on/off	4	#NULL!	#NULL!	1	#NULL!	#NULL!	Acute gastritis	10:54:00 AM	#NULL!	#NULL!	#NULL!		#NULL!	#NULL!
CS31043	0	3	9	1	05-Aug-16	10:49:00 PM	12:00:00 AM	AP ?UTI	4	#NULL!	#NULL!	1	#NULL!	#NULL!	UTI - Urinary tract infection	2:13:00 AM	#NULL!	#NULL!	#NULL!		#NULL!	#NULL!
CS31165	0	3	3	1	05-Aug-16	9:56:00 PM	11:31:00 PM	PV Bleed+cramps(bloods with gp nad)	4	#NULL!	#NULL!	1	#NULL!	#NULL!	Menorrhagia	1:56:00 AM	#NULL!	#NULL!	#NULL!		#NULL!	#NULL!
CS31275	0	3	8	1	05-Aug-16	8:55:00 PM	10:50:00 PM	epigastric pain	3	#NULL!	1	0	#NULL!	#NULL!		12:38:00 AM	#NULL!	#NULL!	#NULL!		#NULL!	#NULL!
CS31564	0	3	1	0	05-Aug-16	4:26:00 PM	5:48:00 PM	not passing urine/feeding unsettled	3	#NULL!	#NULL!	0	#NULL!	#NULL!	Well child	6:52:00 PM	#NULL!	#NULL!	#NULL!		#NULL!	#NULL!
CS31572	0	3	3	0	05-Aug-16	11:20:00 PM	12:43:00 AM	Sickle cell attack	3	#NULL!	#NULL!	1	#NULL!	#NULL!	Sickle cell anemia crisis	3:18:00 AM	#NULL!	#NULL!	#NULL!		#NULL!	#NULL!
CS31639	0	3	8	1	05-Aug-16	8:53:00 AM	9:38:00 AM	HI, unwitnessed, stumbling on street	3	#NULL!	#NULL!	1	#NULL!	#NULL!	Head injury	12:50:00 PM	#NULL!	#NULL!	#NULL!		#NULL!	#NULL!
CS31640	0	3	8	1	05-Aug-16	10:24:00 AM	10:33:00 AM	FALL	3	#NULL!	#NULL!	1	#NULL!	#NULL!	Fall	2:23:00 PM	#NULL!	#NULL!	#NULL!		#NULL!	#NULL!
CS31641	0	3	3	1	05-Aug-16	10:46:00 AM	11:39:00 AM	FEELING FAINT	4	#NULL!	#NULL!	1	#NULL!	#NULL!	Fainting	2:03:00 PM	#NULL!	#NULL!	#NULL!		#NULL!	#NULL!
CS31642	0	3	1	0	05-Aug-16	3:04:00 PM	3:55:00 PM	unwell vomiting yellow	3	#NULL!	#NULL!	1	#NULL!	#NULL!	Vomiting - bile stained	6:01:00 PM	#NULL!	#NULL!	#NULL!		#NULL!	#NULL!
CS31644	0	3	8	1	05-Aug-16	8:17:00 PM	10:06:00 PM	abdo pain	#NULL!	#NULL!	1	1	#NULL!	#NULL!	Gastroenteritis	12:17:00 AM	#NULL!	#NULL!	#NULL!		#NULL!	#NULL!
CS32315	0	3	4	1	05-Aug-16	9:35:00 PM	11:24:00 PM	headaches	3	#NULL!	#NULL!	1	#NULL!	#NULL!	Headache	2:49:00 PM	#NULL!	#NULL!	#NULL!		#NULL!	#NULL!
CS32354	0	3	2	1	05-Aug-16	8:43:00 PM	10:24:00 PM	early pregnant, abdominal pain	3	#NULL!	#NULL!	1	#NULL!	#NULL!	Abdominal pain	12:18:00 AM	#NULL!	#NULL!	#NULL!		#NULL!	#NULL!
CS32524	0	3	9	1	05-Aug-16	2:48:00 PM	3:46:00 PM	FALL unwitnessed, unsteady (dementia)	3	#NULL!	#NULL!	1	#NULL!	#NULL!	Fall - accidental	6:44:00 PM	#NULL!	#NULL!	#NULL!		#NULL!	#NULL!
CS32671	0	3	6	0	05-Aug-16	3:10:00 PM	4:02:00 PM	?SEIZURE, hand was twitching (ca brain)	3	#NULL!	#NULL!	0	#NULL!	#NULL!	Partial seizure	7:10:00 PM	#NULL!	#NULL!	#NULL!		#NULL!	#NULL!
CS32693	0	3	8	1	05-Aug-16	10:20:00 PM	11:23:00 PM	RUQ Pain 3/7	4	#NULL!	1	1	#NULL!	#NULL!	Abdominal pain	2:19:00 AM	#NULL!	#NULL!	#NULL!		#NULL!	#NULL!
CS32713	0	3	9	0	05-Aug-16	4:09:00 PM	4:37:00 PM	UNWELL(bradycardia atropine given)	3	#NULL!	#NULL!	0	#NULL!	#NULL!	Acute confusion	8:09:00 PM	#NULL!	#NULL!	#NULL!		#NULL!	#NULL!
CS32840	0	3	7	0	05-Aug-16	11:06:00 AM	12:02:00 PM	SOB/Right sided CP (PE, pneumonia)	3	#NULL!	#NULL!	1	#NULL!	#NULL!	Pulmonary embolism	3:07:00 PM	#NULL!	#NULL!	#NULL!		#NULL!	#NULL!
CS32973	0	3	6	0	05-Aug-16	6:32:00 PM	9:01:00 PM	Paraphimosis	4	#NULL!	#NULL!	1	#NULL!	#NULL!	Paraphimosis	10:02:00 PM	#NULL!	#NULL!	#NULL!		#NULL!	#NULL!
CS33011	0	3	8	1	05-Aug-16	6:52:00 PM	8:55:00 PM	Suicidal/Intoxicated (etoh)	#NULL!	#NULL!	1	1	#NULL!	#NULL!	Alcohol intoxication (disorder)	10:51:00 PM	#NULL!	#NULL!	#NULL!		#NULL!	#NULL!
CS33793	0	3	7	1	05-Aug-16	2:25:00 PM	3:14:00 PM	COLLAPSED/AP ?seizure	3	#NULL!	#NULL!	1	#NULL!	#NULL!	Small bowel obstruction	6:25:00 PM	#NULL!	#NULL!	#NULL!		#NULL!	#NULL!
CS33926	0	3	7	1	05-Aug-16	11:44:00 AM	12:45:00 PM	painfull red eye]	2	#NULL!	#NULL!	1	#NULL!	#NULL!	Red eye	3:05:00 PM	#NULL!	#NULL!	#NULL!		#NULL!	#NULL!
CS34018	0	3	8	0	05-Aug-16	11:05:00 PM	12:34:00 AM	?UTI	3	#NULL!	#NULL!	1	#NULL!	#NULL!	Urinary tract infection	2:57:00 AM	#NULL!	#NULL!	#NULL!		#NULL!	#NULL!
CS34076	0	3	3	1	05-Aug-16	6:35:00 PM	8:54:00 PM	? chronns flare up/black stools	4	#NULL!	#NULL!	1	#NULL!	#NULL!	Crohn's disease (disorder)	10:34:00 PM	#NULL!	#NULL!	#NULL!		#NULL!	#NULL!
CS31018	1	3	9	1	05-Aug-16	6:26:00 PM	8:19:00 PM	SOB(inpatient langley green)	4	#NULL!	#NULL!	0	1	#NULL!	Pneumonia	4:57:00 AM	1	1.00	########	SPR	0	#NULL!
CS31643	1	3	5	0	05-Aug-16	8:03:00 PM	10:05:00 PM	Flank Pain(renal colic)	3	0	1	0	0	#NULL!	Left flank pain	11:13:00 PM	0	1.00	########	Consutlan	0	#NULL!
CS32456	1	3	7	0	05-Aug-16	7:27:00 PM	9:39:00 PM	sudden onset pain L testis	4	0	#NULL!	0	0	#NULL!	O/E - testicular swelling	11:23:00 PM	0	1.00	########	Consutlan	0	#NULL!
CS30038	0	3	7	0	06-Aug-16	7:45:00 PM	9:01:00 PM	2/7 hx CCP	3	#NULL!	#NULL!	1	#NULL!	#NULL!	Community acquired pneumonia	9:46:00 PM	#NULL!	#NULL!	#NULL!		#NULL!	#NULL!
CS30156	0	3	3	1	06-Aug-16	1:19:00 AM	3:08:00 AM	Headache took 24xParacetamol 500mg+4 kalms	2	#NULL!	1	1	#NULL!	#NULL!	Paracetamol overdose	7:39:00 AM	#NULL!	#NULL!	#NULL!		#NULL!	#NULL!
CS30320	0	3	5	0	06-Aug-16	1:55:00 AM	4:00:00 AM	Assault HI/Lft shoulder pain	4	#NULL!	1	1	#NULL!	#NULL!	Assault	5:55:00 AM	#NULL!	#NULL!	#NULL!		#NULL!	#NULL!
CS30351	0	3	3	1	06-Aug-16	2:02:00 AM	4:16:00 AM	Ear Infection(flucloxacillin)	4	#NULL!	#NULL!	1	#NULL!	#NULL!	Otitis media	5:25:00 AM	#NULL!	#NULL!	#NULL!		#NULL!	#NULL!
CS30392	0	3	7	0	06-Aug-16	7:08:00 PM	8:58:00 PM	R flank pain	4	#NULL!	#NULL!	1	#NULL!	#NULL!	Renal colic	10:24:00 PM	#NULL!	#NULL!	#NULL!		#NULL!	#NULL!
CS30398	0	3	3	0	06-Aug-16	7:00:00 PM	8:58:00 PM	?collapse - been drinking	3	#NULL!	#NULL!	1	#NULL!	#NULL!	Drunk	10:43:00 PM	#NULL!	#NULL!	#NULL!		#NULL!	#NULL!
CS30458	0	3	9	0	06-Aug-16	11:49:00 PM	1:47:00 AM	chest pain	3	#NULL!	#NULL!	1	#NULL!	#NULL!	Stable angina (disorder)	3:49:00 AM	#NULL!	#NULL!	#NULL!		#NULL!	#NULL!
CS30695	0	3	6	0	06-Aug-16	9:49:00 PM	12:02:00 AM	RTC 14hrs ago lower back pain,headache	4	#NULL!	#NULL!	1	#NULL!	#NULL!	Back pain	12:44:00 AM	#NULL!	#NULL!	#NULL!		#NULL!	#NULL!
CS30834	0	3	8	0	06-Aug-16	11:19:00 PM	12:55:00 AM	non epileptic seizure	3	#NULL!	#NULL!	1	#NULL!	#NULL!	Chronic confusion	10:13:00 AM	#NULL!	#NULL!	#NULL!		#NULL!	#NULL!
CS31121	0	3	3	1	06-Aug-16	9:08:00 PM	11:14:00 PM	OD/Mental health	#NULL!	#NULL!	#NULL!	1	#NULL!	#NULL!	Self-discharge	12:26:00 AM	#NULL!	#NULL!	#NULL!		#NULL!	#NULL!
CS31205	0	3	3	1	06-Aug-16	2:22:00 AM	3:53:00 AM	Passing small amounts urine	#NULL!	#NULL!	1	1	#NULL!	#NULL!	Renal colic	5:37:00 AM	#NULL!	#NULL!	#NULL!		#NULL!	#NULL!
CS31252	0	3	2	1	06-Aug-16	2:29:00 AM	5:03:00 AM	smoked cigarette.feels funny	4	#NULL!	1	1	#NULL!	#NULL!	Cigarette consumption	5:42:00 AM	#NULL!	#NULL!	#NULL!		#NULL!	#NULL!
CS31358	0	3	4	1	06-Aug-16	8:00:00 PM	9:39:00 PM	FLANK PAIN, KIDNEY STONES	4	#NULL!	#NULL!	1	#NULL!	#NULL!	Flank pain (finding)	12:00:00 AM	#NULL!	#NULL!	#NULL!		#NULL!	#NULL!
CS31534	0	3	3	1	06-Aug-16	12:45:00 AM	2:08:00 AM	Epigastric Pain	4	#NULL!	1	1	#NULL!	#NULL!	Gastritis	4:39:00 AM	#NULL!	#NULL!	#NULL!		#NULL!	#NULL!
CS31607	0	3	3	1	06-Aug-16	1:41:00 AM	3:08:00 AM	Abdo/back Pain	4	#NULL!	#NULL!	1	#NULL!	#NULL!	Palpitations - fluttering	4:37:00 AM	#NULL!	#NULL!	#NULL!		#NULL!	#NULL!
CS31624	0	3	4	0	06-Aug-16	11:32:00 PM	12:55:00 AM	?Cellulitis/Ulcers Bilateral	2	#NULL!	#NULL!	1	#NULL!	#NULL!	Cellulitis	3:32:00 AM	#NULL!	#NULL!	#NULL!		#NULL!	#NULL!

For peer review only

Supplementary material 2, assessment of clinical adequacy questions

Excel spread sheet sent to each reviewer to complete for all records. One column for each record – reviewer to fill in id number

	Questions *	Insert study ID number from top of record<br>*Drop down response options followed by a cell for free text if appropriate*
1	Record of the patient's medical history	Appropriate<br>Or<br>Error or omission – but unlikely to have resulted in harm or different treatment<br>Or<br>An error or omission seen that caused a breach in normal guidelines and procedures that would have altered the patient's treatment<br>Or<br>An error or omission seen that resulted in significant probability that the patient might be harmed
	Free text on rationale or comment on response to item 1	
2	Examination of the patient	Appropriate<br>Or<br>Error or omission – but unlikely to have resulted in harm or different treatment<br>Or<br>An error or omission seen that caused a breach in normal guidelines and procedures that would have altered the patient's treatment<br>Or<br>An error or omission seen that resulted in significant probability that the patient might be harmed
	Free text on rationale or comment on response to item 2	
3	Request for radiography	Appropriate<br>Or<br>Error or omission – but unlikely to have resulted in harm or different treatment<br>Or<br>An error or omission seen that caused a breach in normal guidelines and procedures that would have altered the patient's treatment<br>Or<br>An error or omission seen that resulted in significant probability that the patient might be harmed
	Free text on rationale or comment on response to item 3	
4	Treatment plan and decision	Appropriate<br>Or<br>Error or omission – but unlikely to have resulted in harm or different treatment<br>Or<br>An error or omission seen that caused a breach in normal guidelines and procedures that would have altered the patient's treatment<br>Or<br>An error or omission seen that resulted in significant probability that the patient might be harmed

For peer review only - http://bmjopen.bmj.com/site/about/guidelines.xhtml

	Questions *	Insert study ID number from top of record<br>*Drop down response options followed by a cell for free text if appropriate*
	Free text on rationale or comment on response to item 4	
5	Treatment plan and decision reviewed by senior doctor	YES<br>Or<br>NO
6	Advice given	Appropriate<br>Or<br>Error or omission – but unlikely to have resulted in harm or different treatment<br>Or<br>An error or omission seen that caused a breach in normal guidelines and procedures that would have altered the patient's treatment<br>Or<br>An error or omission seen that resulted in significant probability that the patient might be harmed
	Free text on rationale or comment on response to item 6	
7	Follow-up	Appropriate<br>Or<br>Error or omission – but unlikely to have resulted in harm or different treatment<br>Or<br>An error or omission seen that caused a breach in normal guidelines and procedures that would have altered the patient's treatment<br>Or<br>An error or omission seen that resulted in significant probability that the patient might be harmed
	Free text on rationale or comment on response to item 7	
8	In your view what type of clinician attended this patient?	Doctor<br>Or<br>Physician associate<br>Or<br>Unable to decide
	Free text on rationale or comment on response to item 8	

* Review questions taken from Sakr et al study [17] comparing patients attended by advanced nurse practitioners with doctors in the ED.

For peer review only - http://bmjopen.bmj.com/site/about/guidelines.xhtml

Supplementary file 3: Observation guide

PA-SCER Physician Associates in Secondary Care in England Research

OBSERVATION: AIDE MEMOIRE for researchers

Our observation aims to support answering the four study research questions but specifically to provide data on impact on organisation of services, other team members working practices and team relationships.

We wish to be able to consider this in terms of:

- Acceptability -how do they appear to be viewed or treated by others?
- Appropriateness – how are they observed in terms of safety e.g. how do they check, how are they checked upon, how are they supervised?
- Equity - who receives the PA service; do any patient groups appear to be over represented?
- Efficiency - how do they appear to contribute to this? How are issues, such as prescribing, worked around?
- Effectiveness – are the outcomes of PA care or contribution to the team observed?

We are observing context, relationships and activities.

Conduct of the observation

- Put up approved notices of our observation activity in the study setting places advised by the clinical team
- Provide the PA with the approved script to inform patients/ patients' representatives to gain permission for the researcher's presence. Each patient is to be asked for permission.
- Researcher to maintain an unobtrusive presence
- Record observations in the ethnographic tradition - take detailed unstructured notes, bearing in mind the importance of capturing context, relationships and activities
- Record as much as possible at the time and add as soon as possible afterwards
- Length of observation to be pre-planned but also to allow for flexibility according to the PA's wishes, the demands of the clinical setting and researcher's length of focus
- Allow the PA to see the notes at any point

After the observation

- Add to the notes as soon as possible where detail was not able to be captured at the time
- Maintain a reflective diary associated with the observation conduct and analytical processes.
- Discuss the observation with local research team members to promote group understanding and consistency across researchers
- Transfer data into NVivo software.

For peer review only - http://bmjopen.bmj.com/site/about/guidelines.xhtml

Supplementary data file 4: Tailored topic guides for the interview

Topic guide for senior managers and clinicians

Topic areas

- Confirm the person's job role
- Ask them to describe their involvement with physician associate employment in the hospital to date
- Ask questions on the factors supporting the adoption of the employment of physician associates
- Ask questions on the factors inhibiting the employment of physician associates
- Questions on their views of physician associates' impact on (ask for examples):
    - Organisation of services
    - Patient experience and outcomes
    - Other staff
    - Costs
- Anything else they would like to say?

Interviewer to probe on all answers to ensure the meaning is clear (e.g. that is an interesting point , can you explain a bit more about it) and check for understanding (e.g. so can I check I have understood you correctly)…….

Interviewer to check for any routine management reports or data or evaluations that the hospital team would be willing to share with the researchers.

Thank them and ask if they would like to receive updates on the study and a final summary of the findings. If so could they please give contact details which will be kept separate from the interview data.

For peer review only

Topic guide for physician associate interviews

Topic areas

- Ask them to describe how long they have been a physician associate, how many posts, type and length as a physician associate
- Ask them to describe the work they undertake, with what type of medical/surgical team
- Ask about their supervising doctor and arrangements when they are not there
- Ask questions on their views of the factors supporting the adoption of the employment of physician associate in their experience

For peer review only - http://bmjopen.bmj.com/site/about/guidelines.xhtml

- Ask questions on their views of the factors inhibiting the employment of physician associate in their experience
- Ask how they have been received in the hospital as a new type of health professional?
- Ask how they explain to patients, family and staff – who they are and what a physician associate is

- Questions on their views of their, or other physician associates, impact on (ask for examples):
    - Organisation of services
    - Patient experience and outcomes
    - Other staff
    - Costs

- Anything else they would like to say?

Interviewer to probe on all answers to ensure the meaning is clear (e.g that is an interesting point , can you explain a bit more about it) and check for understanding (e.g. so can I check I have understood you correctly)…….

Interviewer to check for any routine management reports or data or evaluations that the hospital team would be willing to share with the researchers .

Thank them and ask if they would like to receive updates on the study and a final summary of the findings. If so could they please give contact details which will be kept separate from the interview data.

Topic guide for all other types of professionals/managers interviews

- Confirm the person's job role
- Ask them to describe their involvement with physician associate employment in the hospital to date
- Ask questions on their views of any factors supporting the adoption of the employment of physician associates in their experience
- Ask questions on their views of any factors inhibiting the employment of physician associates in their experience
- Ask their views as to how the PAs have been received in that service/team, and probe for any explanations

For peer review only - http://bmjopen.bmj.com/site/about/guidelines.xhtml

- Questions on their views of physician associates' impact on (ask for examples):
  - Organisation of services
  - Boundaries between the job roles of different types of professionals e.g. with nurses
  - Patient experience and outcomes
  - Other staff
  - Costs

- Anything else they would like to say?

Interviewer to probe on all answers to ensure the meaning is clear (e.g that is an interesting point , can you explain a bit more about it) and check for understanding (e.g. so can I check I have understood you correctly)…….

Interviewer to check for any routine management reports or data or evaluations that the hospital team would be willing to share with the researchers.

Thank them and ask if they would like to receive updates on the study and a final summary of the findings. If so could they please give contact details which will be kept separate from the interview data.

Topic guide for patient interviews

Topic areas

- Confirm the person is/has been a patient
- Ask them to outline the type of care they have been in receipt of without giving personal medical details e.g. in patient for x days
- Confirm the patient has met the physician associate
- Explore what sort of involvement the physician associate has had with them
- Ask them how they understand the role of the physician associate in the medical/surgical team
- Ask them how they found receiving care from a physician associate
- If they were to need similar medical or surgical care, would they be content to receive similar care from a physician associate in the future as they had this time (and can

For peer review only - http://bmjopen.bmj.com/site/about/guidelines.xhtml

they explain why) or would they prefer someone different? And if yes, can they explain why?

- Anything else they would like to say?

Interviewer to probe on all answers to ensure the meaning is clear (e.g. that is an interesting point , can you explain a bit more about it) and check for understanding (e.g. so can I check I have understood you correctly).

Thank them and ask if they would like to receive updates on the study and a final summary of the findings. If so could they please give contact details which will be kept separate from the interview data.

For peer review only

For peer review only - http://bmjopen.bmj.com/site/about/guidelines.xhtml

O'Cathain A, Murphy E, Nicholl J. The quality of mixed methods studies in health services research. J Health Serv Res Policy. 2008;13(2):92-98.

Box 1 Good Reporting of A Mixed Methods Study (GRAMMS)

GRAMMS criterion	Page in manuscript
(1) Describe the justification for using a mixed methods approach to the research question	8
(2) Describe the design in terms of the purpose, priority and sequence of methods	8
(3) Describe each method in terms of sampling, data collection and analysis	8-10
(4) Describe where integration has occurred, how it has occurred and who has participated in it	10
(5) Describe any limitation of one method associated with the present of the other method	18-19
(6) Describe any insights gained from mixing or integrating methods	18-19

For peer review only

No.	Topic	Item
	**Title and abstract**	
S1	Title	Concise description of the nature and topic of the study Identifying the study as qualitative or indicating the approach (e.g., ethnography, grounded theory) or data collection methods (e.g., interview, focus group) is recommended
S2	Abstract	Summary of key elements of the study using the abstract format of the intended publication; typically includes background, purpose, methods, results, and conclusions
	**Introduction**	
S3	Problem formulation	Description and significance of the problem/phenomenon studied; review of relevant theory and empirical work; problem statement
S4	Purpose or research question	Purpose of the study and specific objectives or questions
	**Methods**	
S5	Qualitative approach and research paradigm	Qualitative approach (e.g., ethnography, grounded theory, case study, phenomenology, narrative research) and guiding theory if appropriate; identifying the research paradigm (e.g., postpositivist, constructivist/ interpretivist) is also recommended; rationale[b]
S6	Researcher characteristics and reflexivity	Researchers' characteristics that may influence the research, including personal attributes, qualifications/experience, relationship with participants, assumptions, and/or presuppositions; potential or actual interaction between researchers' characteristics and the research questions, approach, methods, results, and/or transferability
S7	Context	Setting/site and salient contextual factors; rationale[b]
S8	Sampling strategy	How and why research participants, documents, or events were selected; criteria for deciding when no further sampling was necessary (e.g., sampling saturation); rationale[b]
S9	Ethical issues pertaining to human subjects	Documentation of approval by an appropriate ethics review board and participant consent, or explanation for lack thereof; other confidentiality and data security issues
S10	Data collection methods	Types of data collected; details of data collection procedures including (as appropriate) start and stop dates of data collection and analysis, iterative process, triangulation of sources/methods, and modification of procedures in response to evolving study findings; rationale[b]
S11	Data collection instruments and technologies	Description of instruments (e.g., interview guides, questionnaires) and devices (e.g., audio recorders) used for data collection; if/how the instrument(s) changed over the course of the study
S12	Units of study	Number and relevant characteristics of participants, documents, or events included in the study; level of participation (could be reported in results)
S13	Data processing	Methods for processing data prior to and during analysis, including transcription, data entry, data management and security, verification of data integrity, data coding, and anonymization/deidentification of excerpts
S14	Data analysis	Process by which inferences, themes, etc., were identified and developed, including the researchers involved in data analysis; usually references a specific paradigm or approach; rationale [b]
S15	Techniques to enhance trustworthiness	Techniques to enhance trustworthiness and credibility of data analysis (e.g., member checking, audit trail, triangulation); rationale[b]
	**Results/findings**	
S16	Synthesis and interpretation	Main findings (e.g., interpretations, inferences, and themes); might include development of a theory or model, or integration with prior research or theory
S17	Links to empirical data	Evidence (e.g., quotes, field notes, text excerpts, photographs) to substantiate analytic findings
	**Discussion**	
S18	Integration with prior work, implications, transferability, and contribution(s) to the field	Short summary of main findings; explanation of how findings and conclusions connect to, support, elaborate on, or challenge conclusions of earlier scholarship; discussion of scope of application/ generalizability; identification of unique contribution(s) to scholarship in a discipline or field
S19	Limitations	Trustworthiness and limitations of findings
	**Other**	
S20	Conflicts of interest	Potential sources of influence or perceived influence on study conduct and conclusions; how these were managed
S21	Funding	Sources of funding and other support; role of funders in data collection, interpretation, and reporting

[a]The authors created the SRQR by searching the literature to identify guidelines, reporting standards, and critical appraisal criteria for qualitative research; reviewing the reference lists of retrieved sources; and contacting experts to gain feedback. The SRQR aims to improve the transparency of all aspects of qualitative research by providing clear standards for reporting qualitative research.

[b]The rationale should briefly discuss the justification for choosing that theory, approach, method, or technique rather than other options available, the assumptions and limitations implicit in those choices, and how those choices influence study conclusions and transferability. As appropriate, the rationale for several items might be discussed together.

ACADEMIC MEDICINE

S1 p3 (MMR cannot fit in more detail in title)

S2 P3

S3 P7

S4 P7

S5 P8, 9

S6 P10

S7 P8

S8 P9-10

S9 P10

S10 P9-10

S11 P10 (refers to detail elsewhere)

S12 P11

S13 P10 (refers to detail elsewhere)

S14 P10 (refers to detail elsewhere)
S14 As above

S16 P11-17, embedded alongside quant
S17 As above

S18 P17-19

S19 P18-19

S20&21 P5

only

For peer review only

The RECORD statement: Checklist of items, extended from the STROBE statement, that should be reported in observational studies using routinely collected health data.

	Item Number	STROBE Items	RECORD Items	Page number
**Title and Abstract**				
	1	(a) Indicate the study's design with a commonly used term in the title or the abstract. (b) Provide in the abstract an informative and balanced summary of what was done and what was found.	RECORD 1.1: The type of data used should be specified in the title or abstract. When possible, the name of the databases used should be included. RECORD 1.2: If applicable, the geographic region and time frame within which the study took place should be reported in the title or abstract. RECORD 1.3: If linkage between databases was conducted for the study, this should be clearly stated in the title or abstract.	3
**Introduction**				
Background rationale	2	Explain the scientific background and rationale for the investigation being reported.		7
Objectives	3	State specific objectives, including any prespecified hypotheses.		7
**Methods**				
Study Design	4	Present key elements of study design early in the paper.		8
Setting	5	Describe the setting, locations, and relevant dates, including periods of recruitment, exposure, follow-up, and data collection.		8
Participants	6	*(a) Cohort study*: Give the eligibility criteria and the sources and methods of selection of participants. Describe methods of follow-up. *Case-control study*: Give the eligibility criteria and the sources and methods of case ascertainment and control selection. Give the rationale for the choice of cases and controls. *Cross-sectional study*: Give the eligibility criteria and the sources and methods of	RECORD 6.1: The methods of study population selection (such as codes or algorithms used to identify subjects) should be listed in detail. If this is not possible, an explanation should be provided. RECORD 6.2: Any validation studies of the codes or algorithms used to select the population should be referenced. If validation was conducted for this study and not published	8

For peer review only

	Item Number	STROBE Items	RECORD Items	Page number
		selection of participants. *(b) Cohort study*: For matched studies, give matching criteria and number of exposed and unexposed. *Case-control study*: For matched studies, give matching criteria and the number of controls per case.	elsewhere, detailed methods and results should be provided. RECORD 6.3: If the study involved linkage of databases, consider use of a flow diagram or other graphical display to demonstrate the data linkage process, including the number of individuals with linked data at each stage.	
Variables	7	Clearly define all outcomes, exposures, predictors, potential confounders, and effect modifiers. Give diagnostic criteria, if applicable.	RECORD 7.1: A complete list of codes and algorithms used to classify exposures, outcomes, confounders, and effect modifiers should be provided. If these cannot be reported, an explanation should be provided.	10, 12
Data sources/measurement	8	For each variable of interest, give sources of data and details of methods of assessment (measurement). Describe comparability of assessment methods if there is more than one group.		8,10
Bias	9	Describe any efforts to address potential sources of bias.		10
Study size	10	Explain how the study size was arrived at.		9
Quantitative variables	11	Explain how quantitative variables were handled in the analyses. If applicable, describe which groupings were chosen and why.		8,10
Statistical methods	12	(a) Describe all statistical methods, including those used to control for confounding. (b) Describe any methods used to examine subgroups and interactions. (c) Explain how missing data were addressed. (d) *Cohort study*: If applicable, explain how loss to follow-up was addressed. *Case-control study*: If applicable, explain how matching of cases and controls was addressed. *Cross-sectional study*: If applicable, describe analytical methods taking account of sampling strategy. (e) Describe any sensitivity analyses.		10
Data access and cleaning methods		N/A	RECORD 12.1: Authors should describe the extent to which the investigators had access to the database population used to create the study population. RECORD 12.2: Authors	8-9

For peer review only

	Item Number	STROBE Items	RECORD Items	Page number
			should provide information on the data cleaning methods used in the study.	
Linkage		N/A	RECORD 12.3: State whether the study included person-level, institutional-level, or other data linkage across two or more databases. The methods of linkage and methods of linkage quality evaluation should be provided.	8-9
**Results**				
Participants	13	(a) Report the numbers of individuals at each stage of the study (e.g., numbers potentially eligible, examined for eligibility, confirmed eligible, included in the study, completing follow-up, and analysed). (b) Give reasons for nonparticipation at each stage. (c) Consider use of a flow diagram.	RECORD 13.1: Describe in detail the selection of the persons included in the study (i.e., study population selection), including filtering based on data quality, data availability, and linkage. The selection of included persons can be described in the text and/or by means of the study flow diagram.	11, 12, 13
Descriptive data	14	(a) Give characteristics of study participants (e.g., demographic, clinical, and social) and information on exposures and potential confounders. (b) Indicate the number of participants with missing data for each variable of interest. (c) *Cohort study*: summarise follow-up time (e.g., average and total amount).		12
Outcome data	15	*Cohort study*: Report numbers of outcome events or summary measures over time. *Case-control study*: Report numbers in each exposure category or summary measures of exposure. *Cross-sectional study*: Report numbers of outcome events or summary measures.		13
Main results	16	(a) Give unadjusted estimates and, if applicable, confounder-adjusted estimates and their precision (e.g., 95% confidence interval). Make clear which confounders were adjusted for and why they were included. (b) Report category boundaries when continuous variables were categorized. (c) If relevant, consider translating estimates of relative risk into absolute risk for a meaningful time period.		13,14

For peer review only

	Item Number	STROBE Items	RECORD Items	Page number
Other analyses	17	Report other analyses done—e.g., analyses of subgroups and interactions and sensitivity analyses		15
**Discussion**				
Key results	18	Summarise key results with reference to study objectives.		17-18
Limitations	19	Discuss limitations of the study, taking into account sources of potential bias or imprecision. Discuss both direction and magnitude of any potential bias.	RECORD 19.1: Discuss the implications of using data that were not created or collected to answer the specific research question(s). Include discussion of misclassification bias, unmeasured confounding, missing data, and changing eligibility over time, as they pertain to the study being reported.	19
Interpretation	20	Give a cautious overall interpretation of results considering objectives, limitations, multiplicity of analyses, results from similar studies, and other relevant evidence.		19
Generalisability	21	Discuss the generalisability (external validity) of the study results.		18-19
**Other Information**				
Funding	22	Give the source of funding and the role of the funders for the present study and, if applicable, for the original study on which the present article is based.		5
Accessibility of protocol, raw data, and programming code		N/A	RECORD 22.1: Authors should provide information on how to access any supplemental information such as the study protocol, raw data, or programming code.	24

N/A, not applicable

For peer review only

BMJ Open

Comparing physician associates and foundation year two doctors in training undertaking emergency medicine consultations in England: a mixed methods study of processes and outcomes

Journal:	*BMJ Open*
Manuscript ID	bmjopen-2020-037557.R1
Article Type:	Original research
Date Submitted by the Author:	03-Jun-2020
Complete List of Authors:	Halter, Mary; Kingston University and St George's, University of London, Faculty of Health, Social Care and Education<br>Drennan, Vari; Kingston University Faculty of Health Social Care and Education, Centre for Health & Social Care Research<br>Wang, Chao; Kingston University Faculty of Health Social Care and Education,<br>Wheeler, Carly; Imperial College Healthcare NHS Trust, Centre for Medication Safety and Service Quality<br>Gage, Heather; University of Surrey, School of Economics<br>Nice, Laura; University of Birmingham,<br>de Lusignan, Simon; University of Oxford, Nuffield Department of Primary Care Health Sciences; University of Surrey, Department of Clinical and Experimental Medicine<br>Gabe, Jonathan; Royal Holloway University of London, School of Law and Social Science<br>Brearley, Sally; Kingston University and St George's, university of London, Faculty of Health, Social Care and Education<br>Ennis, James; University of Chester, Chester Medical School<br>Begg, Phil; Royal Orthopaedic Hospital, Executive<br>Parle, Jim; University of Birmingham, Institute of Clinical Sciences
**Primary Subject Heading**:	Emergency medicine
Secondary Subject Heading:	Health services research, Qualitative research
Keywords:	ACCIDENT & EMERGENCY MEDICINE, Organisation of health services < HEALTH SERVICES ADMINISTRATION & MANAGEMENT, Quality in health care < HEALTH SERVICES ADMINISTRATION & MANAGEMENT

SCHOLARONE™
Manuscripts

I, the Submitting Author has the right to grant and does grant on behalf of all authors of the Work (as defined in the below author licence), an exclusive licence and/or a non-exclusive licence for contributions from authors who are: i) UK Crown employees; ii) where BMJ has agreed a CC-BY licence shall apply, and/or iii) in accordance with the terms applicable for US Federal Government officers or employees acting as part of their official duties; on a worldwide, perpetual, irrevocable, royalty-free basis to BMJ Publishing Group Ltd ("BMJ") its licensees and where the relevant Journal is co-owned by BMJ to the co-owners of the Journal, to publish the Work in this journal and any other BMJ products and to exploit all rights, as set out in our licence.

The Submitting Author accepts and understands that any supply made under these terms is made by BMJ to the Submitting Author unless you are acting as an employee on behalf of your employer or a postgraduate student of an affiliated institution which is paying any applicable article publishing charge ("APC") for Open Access articles. Where the Submitting Author wishes to make the Work available on an Open Access basis (and intends to pay the relevant APC), the terms of reuse of such Open Access shall be governed by a Creative Commons licence – details of these licences and which Creative Commons licence will apply to this Work are set out in our licence referred to above.

Other than as permitted in any relevant BMJ Author's Self Archiving Policies, I confirm this Work has not been accepted for publication elsewhere, is not being considered for publication elsewhere and does not duplicate material already published. I confirm all authors consent to publication of this Work and authorise the granting of this licence.

For peer review only

Comparing physician associates and foundation year two doctors in training undertaking emergency medicine consultations in England: a mixed methods study of processes and outcomes

Corresponding author

Mary Halter
Faculty of Health, Social Care and Education, Kingston University St George's, University of London, Cranmer Terrace, London SW17 0RE, UK. m.halter@sgul.kingston.ac.uk 020 8725 0337

Co-authors

Vari M Drennan
Faculty of Health, Social Care and Education, Kingston University St George's, University of London, London, UK
V.Drennan@sgul.kingston.ac.uk

Chao Wang
Faculty of Health, Social Care and Education, Kingston University St George's, University of London, Cranmer Terrace, London, UK
C.Wang@sgul.kingston.ac.uk

Carly Wheeler
Centre for Medication Safety and Service Quality, Imperial College Healthcare NHS Trust, London, UK
carly.wheeler@gmail.com

Heather Gage
Surrey Health Economics Centre, Department of Clinical and Experimental Medicine, University of Surrey, Guildford, UK
h.gage@surrey.ac.uk

Laura Nice
Institute of Clinical Sciences, University of Birmingham, UK
L.Nice@bham.ac.uk

For peer review only

Simon de Lusignan
Nuffield Department of Primary Care Health Sciences, Oxford, UK
simon.delusignan@phc.ox.ac.uk

Jonathan Gabe PhD
School of Law and Social Science, Royal Holloway, University of London, Egham, UK
J.Gabe@rhul.ac.uk

Sally Brearley
Faculty of Health, Social Care and Education, Kingston University St George's, University of London, Cranmer Terrace, London, UK
S.Brearley@sgul.kingston.ac.uk

James Ennis
Chester Medical School, University of Chester, UK
j.ennis@chester.ac.uk

Philip Begg
The Royal Orthopaedic Hospital Birmingham, UK
philip.begg@nhs.net

Jim Parle
Institute of Clinical Sciences, University of Birmingham, UK
j.v.parle@bham.ac.uk

Keywords

Physician Assistants; physician associates; Emergency Service, Hospital; emergency department; medical education

Word count:
Abstract 300
Main text 3960

For peer review only

Comparing physician associates and foundation year two doctors in training undertaking emergency medicine consultations in England: a mixed methods study of processes and outcomes

ABSTRACT

Objectives

To compare the contribution of physician associates to the processes and outcomes of emergency medicine consultations to that of foundation year two doctors-in-training.

Design

Mixed methods study: retrospective chart review using four months' anonymised clinical record data of all patients seen by physician associates or foundation year two doctors-in-training in 2016; review of a sub sample of 40 records for clinical adequacy; semi-structured interviews with staff and patients; observations of physician associates.

Setting

Three emergency departments in England

Participants

The records of 8816 patients attended by six physician associates and 40 foundation year two doctors-in-training; of these n=3197 had the primary outcome recorded (n=1129 PA, n=2068 doctor); 14 clinicians and managers and six patients or relatives for interview; five phyisican associates for observation.

Primary and secondary outcome measures

The primary outcome was unplanned re-attendance at the same emergency department within seven days. Secondary outcomes: consultation processes, clinical adequacy of care, and staff and patient experience.

Results

Re-attendances within seven days (n=194 [6.1%]) showed no difference between physician associates and foundation year two doctors-in-training (OR 0.87 [95% CI 0.61, 1.24], p=0.437). If seen by a physician associate, patients were more likely receive an x-ray investigation (OR 2.10 [95% CI 1.72, 4.24)] , p<0.001), , after adjustment for patient characteristics, triage severity of condition and statistically significant clinician intra-class correlation. Clinical reviewers found almost all patients' charts clinically adequate. Physician associates were evaluated as assessing patients in a similar way to foundation year two doctors-in-training and providing continuity in the team. Patients were

For peer review only - http://bmjopen.bmj.com/site/about/guidelines.xhtml

positive about the care they had received from a physician associate, but had poor understanding of the role.

Conclusions

Physician associates in emergency departments in England treated patients with a range of conditions safely, and at a similar level to foundation year two doctors-in-training, providing clinical operational efficiencies.

For peer review only

For peer review only - http://bmjopen.bmj.com/site/about/guidelines.xhtml

Article summary

Strengths and limitations of this study

- This study provides a well-powered quantitative comparative analysis of the documented processes and outcomes of patient care by physician associates and foundaion year two doctors-in-training in three emergency departments in different parts of England.
- We believe this to be the first empirical study of the outcomes of care provided by UK-trained physician associates in the emergency department, and the first internationally to include interview and observation data.
- Patients' views have not been previously reported for physician associates in this setting.
- The low sensitivity of the emergency department triage system to identify conditions other than the most serious was a problem and impaired the study's ability to describe case mix fully.

The original protocol for the study

The protocol for the study is available at the funding body's website https://www.journalslibrary.nihr.ac.uk/programmes/hsdr/141926/#/

Funding

This project was funded by the National Institute for Health Research Health Services and Delivery Research Programme (project number 14/19/26). This paper presents independent research commissioned by the National Institute for Health Research (NIHR). The views and opinions expressed by authors in this publication are those of the authors and do not necessarily reflect those of the NHS, the NIHR, the Health Service and Delivery Research Programme or the Department of Health.

Competing Interests

SdeL was head of the Department of Clinical and Experimental Medicine until June 2019 at the University of Surrey, which launched a physician associate course in 2016. JP is the immediate past chair of the UK and Ireland Board for Physician Associate Education and immediate past director of the physician associate programme at the University of Birmingham. PB is honorary faculty at the University of Birmingham and has taught on the physician associate programme since 2008. JE taught part time on the University of Birmingham physician associate programme until 2020. VMD was a HS&DR Board Member in 2015.

For peer review only

Main text

Introduction

Health care systems internationally are challenged to ensure good patient outcomes, within financial constraints, as well as to attend to the work life of the workforce.[1] Health workforce shortages, particularly of doctors, are resulting in the development of advanced clinical practitioners or non-physician clinicians (NPCs), such as nurse practitioners (NPs) and physician assistants/associates (PAs) in many countries.[2] Numerous countries are experiencing rising patient demand for emergency services and concommittant shortages of doctors in emergency medicine.[3-7] This situation has led to the development of NPC roles in emergency departments (EDs) in many countries such as the United States (US),[8] Australia,[9] Canada,[10] and the United Kingdom (UK).[11] In the US, 25% (n=14,360) of all emergency medicine clinicians are NPCs, and 68% of these are PAs.[8]

PAs are trained in the medical model to take histories, diagnose illness, develop management plans and prescribe medications as agreed with their supervising physician. PAs have a fifty year history in the US and are a developing part of the workforce in some other countries such as Canada, the Netherlands and Germany.[12] The PA workforce is growing in the UK (where they are known as physician associates). In 2018 there was an estimated 600 qualified PAs with approximately 1000 graduating each year since then.[11] Their employment specialties include EDs,[11] where they are deployed in both the minor and the major illness or injury sections.[8]

Descriptive observations have been published concerning the positive contributions by US-trained PAs employed in EDs in the UK,[13] Australia and New Zealand,[14] and by UK-trained PAs in England.[15] Unlike in the US, PAs in these other countries cannot prescribe medicines or order ionising radiation. PAs in North American EDs are reported to be well accepted by other staff and patients, and reliable in assessing certain medical complaints and performing procedures.[16] No difference is reported between patients attended by a PA and those attended by a doctor for wound infection rates, or rate of revisit within 72 hours to a pediatric ED; but studies find less consistency in practice when analysing prescribing patterns, length of stay and wait times of physicians, PAs and NPs in the ED.[17] There is relatively little research evidence on their clinical effectiveness,[17] little quantitative evidence on outcomes from outside of the US and no qualitative evidence of how PAs deliver care in the ED. In this context our goal was to investigate the contribution of PAs to the processes and outcomes of emergency medicine consultations compared to that of foundation year two (FY2) doctors-in-training in EDs in English hospitals.

For peer review only

Methods

Study design

We conducted a pragmatic, mixed methods convergence study in which we compare and contrast and simultaneously interpret quantitative and qualitative data[18] in three EDs in England, with three components. We undertook a quantitative observational retrospective chart review of patient consultations by PAs compared with FY2 doctors-in-training; and qualitatively we directly observed PAs' practice; and we conducted semi-structured interviews with members of the staff team. Our planned prospective study of patient records with a linked patient satisfaction and outcomes survey had to be revised to a pragmatic retrospective chart review due to practicalities within the participating NHS organisations in the period of the study.

Population and sampling

Three consultant-led, 24 hour EDs with full resuscitation facilities ('type one') participated. Two EDs had annual attendances in the range of 100,000 – 120,000 adult and pediatric patients and the third in the range of 170,000 -190,000. One was an university hospital; two were district general hospitals. The hospitals had been recruited as part of a larger study investigating the work and contribution of PAs between 2016 and 2017.[19] We selected FY2 doctors-in-training as the comparator for PAs, as PAs are offered as part of a solution to junior medical workforce shortages[7] and the most junior doctors working in the UK ED at the time were FY2s.

Selection of participants, measurements and outcomes

Our primary outcome was unplanned re-attendance at the same ED within seven days - one of the NHS clinical quality indicators for EDs in England.[20] Our secondary outcomes were: consultation processes (length of time in the ED, use of x-ray, prescriptions and referrals); clinical adequacy of care, referrals and planned follow up; and patient experience.

Chart review

For a 16-week period (the standard duration of ED placement for FY2 doctors-in-training in the UK), we obtained anonymised, routinely-collected electronic records of all patients attended by a PA or FY2 doctor-in-training, provided in Microsoft Excel by the hospital information teams in each trust, using queries based on staff job role, dates and requested data items. Hospital staff extracted additional data items (supplementary material 1) – age, sex, acuity (as categorised by the Manchester

For peer review only - http://bmjopen.bmj.com/site/about/guidelines.xhtml

triage score [21]), x-ray orders, diagnosis, prescription issued, admission, area treated, overall time in the ED (from check in to discharge, in minutes), and re-consultation within seven days (the primary outcome). No data linkage was required. The researchers did not have access to the original data set and so could not identify if any patients appeared more than once in the dataset, and further data cleaning could not be performed.

We calculated a sample size for the primary outcome based on rate of 18.3% (the highest of two rates for nurse practitioners substituting for physicians, [at 28 days]).[22,23] Aiming to find a relative difference of 50%, in a non-inferiority hypothesis, we required 284 patients in each group (calculation from Stata v11.1 software) to compare 18.3% to 27.4% unplanned re-consultations, with conventional 80% power at 5% significance. We included an extra 20 to allow for adjustment for case mix, requiring a minimum of 304 patients in total in each group to achieve the said power. As 28 day data could not be collected we went on to use the seven day reattendance rate, with its national average of 7.4% (range 2.4% to 21.7%) for unplanned re-attendance at the same ED in England within for all patients.

Two of the participating EDs also agreed to take part in the analysis of clinical adequacy of documented care in every tenth case from the chart review sample (n=40), with equal numbers of cases seen by PA and FY2 doctors-in-training, and using the full anonymised clinical record. We recruited two specialty registrars (doctors in their sixth year of emergency medicine training), one PA lecturer with 20 years ED experience, and one emergency medicine consultant (with 17 years experience at consultant level) from outside the three study hospitals to review these records. All four clinicians independently recorded their judgement as to the clinical adequacy of care for each record using the categories of past medical history, examination, request for radiography, treatment plan and decision, advice given and follow up. Their assessments were blinded to the type of professional attending the patient and to each other's assessment, using a proforma (supplementary material 2) based on published studies.[22,23] As the senior clinician, we accepted the decision of the consultant in cases of disagreement.

Observation

This element drew on the ethnographic tradition used in many health service research studies.[24] We invited all PAs working in the ED in our three study hospitals to participate (n=6). Five PAs volunteered and gave written informed consent to be observed. One of three researchers (CWh, LN, MH) observed each PA for two or three pre-arranged sessions, of varying lengths, on weekdays in periods between 08.00 and 22.00, following a broad guide (supplementary material 3). Researchers made notes on context, relationships and activities following this guide.We judged data saturation to

For peer review only - http://bmjopen.bmj.com/site/about/guidelines.xhtml

have been reached with individual PAs when the processes of care observed did not differ significantly from previous observations. During the observation period, PAs asked for patient assent to the researcher's presence. Researchers reflected on the observations, discussing them in pairs.

Interview

Semi structured interviews [25] were undertaken with a purposive sample of managerial, medical and nursing ED staff who volunteered after receiving information about the study from the researcher during observation periods and/or via their site manager. We also opportunistically interviewed patients and/or their relative who were being seen by a PA in the ED, identified and invited to particpate during observation periods, once they had been assessed and treated by the PA but before discharge from the ED. We used tailored topic guides (supplementary material 4) to explore interviewees' perceptions of the PA role and its impact on service organization, role boundaries, patient experience, patient outcomes, and activities and attitudes of other staff. We digitally recorded interviews or took notes if the participant preferred. Recordings were transcribed verbatim and anonymised.

Analysis

Chart review: The characteristics of patients treated by PAs and FY2 doctors-in-training were compared using chi-squared tests. We carried out a logistic regression to examine whether the primary and binary secondary outcomes differed between PAs and FY2 doctors-in-training, while adjusting for confounding factors - patient age, sex and triage score. Since patients seen by the same clinician are likely to be correlated, we calculated intraclass correlation coefficients (ICC) for each outcome and report results using a random-effects model if the ICC is statistically significant. We report odds ratios, their confidence intervals (CI), and two-tailed p values. For length of stay, a linear regression was used for data transformed to logarithm scale to reduce heteroscedasticity and reflect the fact that the value of length of stay is positive. To account for unobserved heterogeneity, the unobserved component is modelled as a latent variable in a latent class linear model. The assessment of clinical adequacy is reported using descriptive statistics, sensitivity and specificity of the judgment of whether the record was that of a PA or FY2 doctor-in-training, and Fleiss kappa for inter-rater agreement, calculated for each of the four components of the assessment .

Qualitative: Our methods for the analysis of observation data drew on methods to identify ethnographic vignettes.[26] We employed thematic analysis[27] of all-specialty interview data for the wider study. Both are described in full elsewhere.[19] For the subsequent specialty-specific analysis we re-read all ED observation data and interview transcripts to identify all data related to the primary and secondary outcomes, and which both confirmed or disconfirmed findings.

For peer review only - http://bmjopen.bmj.com/site/about/guidelines.xhtml

Mixed methods: Following the separate quantitative and qualitative analyses, we (MH and VMD, in consultation with all authors) merged [18] the quantitative and qualitative datasets by presenting the quantitative results by study outcomes and following these with qualitative data findings (themes and/or excerpts or quotes) that confirmed or disconfirmed the quantitative results.

Ethical approval

We gained approval from the NHS Health Research Authority London-Central Research Ethics Committee (15/LO/1339).

Results

Characteristics of chart review subjects

In the 16 week period studied, 8,816 patients seen by six PAs (n=2890) or forty FY2 doctors-in-training (n=5926) were identified; some secondary outcomes were available for all cases. For 3197 of these patient episodes (n=1129 by the six PAs and n= 2068 by 22 FY2 doctors-in-training) the primary outcome was collected at site for the research team. Characteristics of the patients are shown in Table 1. PAs saw a lower proportion of patients categorised on triage into the urgent category than FY2 doctors-in-training.

In interview, the type of patient seen, patient throughput and role of PAs and FY2 doctors-in-training were described as similar:

They're [the PAs] pretty much equal to .....a senior FY2 doctor in training level. As a consultant we feel comfort because we know [PA name 1] can work in majors, she can clear [majors] pretty much...... And [PA name 2],... can clear paeds minors... Participant 150 Emergency Medicine consultant

However more than one participant tentatively suggested that PAs saw the less acutely unwell patients.:

So my understanding is like they're [the PAs] equivalent to, I would put it like a certain level of like a junior physician.....I wouldn't say they would be at registrar level.....I'd put them somewhere in between. You know a...lot better than like a newly qualified physician because they've got the skills and stuff, so in that gap of what I would say equivalent to maybe like a second to four years post qualified doctor. Participant 144, Registrar

Characteristics of interview and observation participants

The staff interviewed included four PAs, two managers, five nurses and three senior doctors; six patients and/or their relatives were also interviewed, spread across the three sites. We observed four PAs, at three sites; we do not report further demographic details due to concerns about anonymity in a small population.

For peer review only

1 Table 1 Characteristics of chart review sample

Characteristic	PA (N = 2381) N (%)	FY2 doctor (N = 6435) N (%)	Total (N = 8816) N (%)	p-value
**Age band**				
0-20	300 (13.0%)	656 (10.3%)	956 (11.0%)	0.002
21-40	543 (23.5%)	1493 (23.5%)	2036 (23.5%)	
41-60	530 (22.9%)	1406 (22.1%)	1936 (22.3%)	
61-80	551 (23.8%)	1596 (25.1%)	2147 (24.7%)	
81 and over	390 (16.9%)	1212 (19.0%)	1602 (18.5%)	
Unknown				
**Sex**				
Male	1132 (47.5%)	2933 (45.6%)	4065 (46.1%)	0.102
Female	1249 (52.5%)	3501 (54.4%)	4750 (53.9%)	
Unknown				
**Manchester triage score**				
1 Immediate	10 (0.6%)	3 (0.1%)	13 (0.2%)	<0.001
2 Very urgent	163 (9.3%)	565 (11.1%)	728 (10.6%)	
3 Urgent	770 (43.8%)	2841 (55.7%)	3611 (52.6%)	
4 Standard	811 (46.1%)	1681 (32.9%)	2492 (36.3%)	
5 Non Urgent	5 (0.3%)	12 (0.2%)	17 (0.2%)	
Unknown				
**ED area treated in**				
Minor	369 (20.1%)	275 (6.8%)	644 (10.9%)	<0.001
Major	1266 (68.8%)	3601 (88.4%)	4867 (82.3%)	
Resuscitation	2 (0.1%)	4 (0.1%)	6 (0.1%)	
Paediatrics	181 (9.8%)	174 (4.3%)	355 (6.0%)	
Clinical decision unit or primary care	21 (1.1%)	20 (0.5%)	41 (0.7%)	
Unknown				

For peer review only - http://bmjopen.bmj.com/site/about/guidelines.xhtml

The primary outcome: rate of return to the ED within seven days

Re-attendance within seven days was found following 2.2% (N = 194) of the 3197 index visits for which these data were available. The high rate of unknown data is accounted by one site where these data were not captured in the electronic dataset and were only retrieved manually for a random sample (n=205) for the purposes of this study. After adjustment for confounding, no statistically significant difference was found for cases seen by PAs or FY2 doctors-in-training; see Table 2.

Table 2: Re-attendance at the same ED within seven days

**Re-attendance at the same ED within seven days**	PA (N = 1129)	FY2 doctor (N = 2068)	Total (N = 3197)	Unadjusted odds ratio (95% CI) and p-value (PA relative to FY2 doctor-in-training) in rate of re-attendance	Adjusted odds ratio (95% CI) and p-value (PA relative to FY2 doctor-in-training) in rate of re-attendancee†
No	1066 (94.4%)	1937 (93.7%)	3003 (93.9%)	0.87 (0.64, 1.19) p=0.388	0.87 (0.61, 1.24) p=0.437
Yes	63 (5.6%)	131 (6.3%)	194 (6.1%)		
Unknown	1251	4368	5619	-	-

†Adjustment made for triage score (as a measure of acuity), age band, sex, admission, x-ray and site; no adjustment was made for clustering as the ICC by individual staff member on outcome was small (0.008) and statistically insignificant (p= 0.236).

Secondary outcome: consultation processes

No differences were found between patients attended by PAs or by FY2 doctors-in-training in: whether prescriptions were given, admission to hospital from the ED, or if a discharge summary was completed. However, patients seen by a PA were more likely to have an x-ray performed in the ED (Table 3), less likely to be admitted to hospital, and to have a shorter length of stay in the ED (by 35 minutes), after adjustment for age, sex, acuity, whether admitted, x-ray taken, and site, as well as for clustering by individual clinician, although no account was able to be taken of the staffing level.

We observed PAs being the first member of the medical team to carry out assessment of patients following triage to either the major, minor or paediatric areas of the ED. We noted that PAs saw patients independently, following a medical history taking and examination model, before reporting in

For peer review only

person to the senior ED physician in the same way as nurse practitioners and FY2 doctors-in-training did.

For peer review only

Table 3 Clinical process measures

Clinical process measure	PA (N = 2381)	FY2 doctor (N = 6435)	Total (N = 8816)	Unadjusted odds ratio (95% CI) and p-value (PA relative to FY2 doctor-in-training) in rate of re-attendance	Adjusted odds ratio (95% CI) and p-value (PA relative to FY2 doctor-in-training) in rate of re-attendance†
**X-ray investigations performed**					
No	559 (49.4%)	1701 (82.3%)	2260 (70.7%)	4.76 (4.04, 5.59) p<0.001	2.70 (1.72, 4.24) p<0.001
Yes	572 (50.6%)	366 (17.7%)	938 (29.3%)		
Unknown	1250	4368	5618	-	-
**Prescriptions given in the ED**					
No	174 (58.0%)	157 (51.8%)	331 (54.9%)	0.79 (0.56, 1.07) p=0.127	1.35 (0.08, 23.5) p=0.838
Yes	126 (42.0%)	146 (48.2%)	272 (45.1%)		
Unknown	2081	6132	8213		
**Admitted as an inpatient from the ED**					
No	883 (78.2%)	1436 (70.1%)	2319 (73.0%)	0.65 (0.55, 0.77) p<0.001	0.78 (0.55, 1.1) p=0.158
Yes	246 (21.8%)	613 (29.9%)	859 (27.0%)		
Unknown	1762	3876	5638		
**Discharge summary completed**					
No	86 (42.4%)	71 (34.6%)	157 (38.5%)	0.72 (0.48, 1.08) p=0.109	1.57 (0.93, 2.66) p=0.09
Yes	117 (57.6%)	134 (65.4%)	251 (61.5%)		
Unknown	2178	6230	8408		

†Adjustment made for MTS (as a measure of acuity), age band, sex, and site, and for clustering where the ICC (and p-value) is significant: x-ray 0.04 (p<0.001), prescriptions 0.73 (p<0.001), admitted 0.02 (p=0.001), discharge summary <0.001 (p=0.498)

PAs were differentiated from FY2 doctors-in-training by many of our interviewees for not being able to prescribe medications or order tests utilising ionising radiation. Some participants considered this to have a detrimental impact on PAs and patients:

For peer review only

[prescribing] would make a massive difference for them as well and [for] patients because at the end of the day they're having to wait for the PAs to go talk through [with] the physicians what's going on and then probably see somebody else. Participant 118 Nurse practitioner

However, PAs were observed taking on several roles in relation to prescriptions and x-ray orders, for example suggesting medications to or charting the medication for a senior doctor to sign off:

So when one of my PAs comes to me and says "This patient has a temperature of 38, they're coughing up horrible green sputum and they're tachycardic and I listened to their chest and they've got crackles at the left base, can we order a chest x-ray and prescribe sepsis drugs for, you know, pneumonia?" I say "Yes" and I sign it. With probably more confidence at this stage having had [number] PAs here for a year than I would with a junior physician in training on day two. And the irony of that is of course, the junior physician in training doesn't need to come and ask me, technically, they can prescribe themselves. Participant 21 Emergency Medicine consultant

PAs were also observed making referrals to medical and surgical teams outside of the ED, completing discharge summary information, and carrying out procedures, most commonly cannulation, phlebotomy and suturing.

Secondary outcome: clinical adequacy

Our reviewers found the chart documentation to have been 'appropriate' or 'with no errors or omissions that resulted in significant probability that the patient might be harmed' in 36/40 cases for all of the key consultation components (Table 4). In the three records (two of FY2 doctors-in-training and one of a PA) judged as having errors or omissions at the level of a breach in normal guidelines and procedures that would have altered the patient's treatment, all reviewers agreed that a senior doctor review had occurred in one case; this was unclear in the other cases. Our observation data suggest that such a senior review was undertaken for all assessment and clinical decision making in the 'majors' sections of the ED, but that 'minors' care was often completed independently.

Our reviewers were 40% sensitive, 46% specific on judging the clinician type: 68% (13/19) of the PA records were thought to be of a FY2 doctor-in-training and 60% (9/15) vice versa (kappa score for inter-rater agreement 0.15).

For peer review only

For peer review only - http://bmjopen.bmj.com/site/about/guidelines.xhtml

Table 4: Chart reviewers’ assessments of clinical adequacy

PA or FY2 consultation record		Judgment of appropriateness: Past medical history				Examination				Request for radiography*				Treatment plan and decision				Advice given				Follow up			
		Appropriate	Error or omission			Appropriate	Error or omission			Appropriate	Error or omission			Appropriate	Error or omission			Appropriate	Error or omission			Appropriate	Error or omission		
			Harm unlikely	Altered treatment	Harm		Harm unlikely	Altered treatment	Harm		Harm unlikely	Altered treatment	Harm		Harm unlikely	Altered treatment	Harm		Harm unlikely	Altered treatment	Harm		Harm unlikely	Altered treatment	Harm
FY2	n	14	6	0	0	15	5	0	0	9	0	1	0	14	5	1	0	4	1	1	0	16	3	0	0
	%	70	37	0	0	75	25	0	0	45	0	5	0	70	25	5	0	20	5	5	0	80	15	0	0
PA	n	13	5	1	0	11	7	1	0	9	3	0	0	13	5	1	0	3	1	1	0	13	4	1	0
	%	65	25	5	0	55	35	5	0	45	15	0	0	65	25	5	0	15	5	5	0	65	20	5	0
Not rated*	n	1				1				18				1				29				3			
	%	2.5				2.5				45				2.5				73				7.5			
Total	n	27	11	1	0	26	12	1	0	18	3	1	0	27	10	2	0	7	2	2	0	29	7	1	0
	%	68	28	3	0	65	30	3	0	45	8	3	0	66	25	5	0	18	5	5	0	73	18	3	0
Agreement (Fleiss kappa, combined)		0.01				0.15				0.26				0.15				-0.03				0.30			
Agreement (Fliess kappa, by response)		0.04	-0.02	-0.04	n/a	0.17	0.12	0.15	n/a	0.29	0.11	0.33	n/a	0.24	0.01	0.14	n/a	-0.00	0.11	0.08	n/a	0.36	0.20	0.28	n/a

*Missing rating or rated as ‘not applicable’ if no request for radiography was made or no advice given

For peer review only

Interviewees also presented other aspects related to clinical adequacy, particularly the PAs' stability in the team. The clinicians' familiarity with the longer standing team member PA/s - in contrast to FY2 doctors-in-training on rotation - was raised repeatedly:

> *If there was a junior physician over here, and he said oh, what do you think of this wound, which they do ask us. And I say yeah, it needs suturing. I then have to say, but can you suture or do you want me to suture it?.....Because I don't know, and some will say oh no, I can't.....I've never sutured before, and some will say oh yeah, that's fine, I'll suture it.....Whereas I know with PAs they'll suture their own. Because I know that they've got that skill set.* Participant 177 Advanced nurse practitioner

Secondary outcome: patient experience

Patients were positive about the care they had received from the PA, but had not understood what the PA role meant, with two participants believing they had been seen by a doctor and another unsure in the context of multiple ED staff:

> *I presumed he was a fully-qualified physician, yes his approach and everything was absolutely 100%.* Participant 120 Patient

Most of our patient participants were receptive to the role on the grounds that it might speed up care, although they were not without concern for the difference in training from a doctor and the diminishment of a senior medical workforce:

> *It's good to have another person, another opinion…but would it not perhaps be better to have another doctor?* Participant 083 Patient's relative

Discussion

Summary of findings

The study presents evidence from three English EDs and has demonstrated no difference in safety or appropriateness between PAs and FY2 doctors-in-training. We report no difference in re-attendance rates. Those patients seen by a PA (within PA working hours 08-22.00) had a shorter average length of stay in the ED than those seen by doctors-in-training (24 hour working period). Our review of clinical adequacy found few errors and no difference between PAs and FY2 doctors-in-training. Patients appeared relatively unconcerned with the title of the clinician treating them and thought they

For peer review only - http://bmjopen.bmj.com/site/about/guidelines.xhtml

had been treated by a doctor; however they were keen to know that the employment of PAs would not represent a widespread substitution for doctors in the ED.

How this study is similar or different from prior studies

We believe this to be the first empirical study of the outcomes of care provided by UK-trained PAs in the ED, and the first internationally to include interview and observation data. Additonally, patients' views do not appear to have been previously gathered at the time of the visit (and qualitaitvely), although there have been previous questionnaire studies in the USA of patient satisfaction, administered after the visit.[28,29,30]

We reported few differences in the the practice and processes of care – other than prescribing (which PAs currently cannot do independently in the UK) – between PAs and doctors in their second foundation year of training. Our finding of no difference in the primary outcome (ED reattendance rate within seven days) for patients of PAs and FY2 doctors-in-training is consistent with the comparisons of nurse-qualified NPCs and FY2 doctors-in-training on which we based our study design [22,23] and other PA literature from the USA.[16] It should be noted that for patients in the majors section of ED, all assessment and treatment plans by FY2 doctors-in-training and NPCs were reviewed and agreed by a senior clinician. Our participants commented frequently on the transient nature of FY2 doctors-in-training, whose rotation in the ED only last four months. In contrast, PAs remained long-term and provided continuity in the team. Their accumulated knowledge of the policies and practices (clinical and otherwise) of the department, the consultants and the hospital was reported to enable operational efficiencies. Simlar observations about PAs providing continuity within the medical/surgical team have been made in North America and the Netherlands[31-33] and also for other NPCs.[34]

This study's strengths lie in its mixed-methods approach to the study of PAs in the ED, allowing consideration of different types of data on their contribution, compared to that of FY2 doctors-in-training, to be considered. We were able to carry out a well-powered quantitative comparative analysis of the documented processes and outcomes of patient care by PAs and FY2 doctors-in-training in three EDs in different parts of the country, and to gather qualitative data on PAs 'in practice'. The qualitative component of our mixed methods approach enabled contextual explanations of the quantitative analysis.

Our study however has several limitations. Our comparison of PAs and doctors working in all areas of the ED introduced the potential for PAs and FY2 doctors-in-training to be attending to patients of different acuity and complexity. We sought to mitigate this by using three different EDs, taking a

For peer review only - http://bmjopen.bmj.com/site/about/guidelines.xhtml

sample across a 16 week period at all times of day and night (although the FY2 doctors-in-training worked over the 24 hour period when staff:patient ratios may have fluctuated). We also made statistical adjustments that included triage category. The low sensitivity of most ED triage systems to identification of conditions other than the most serious, however, is a drawback.[35] The prevention of collection of 28 day outcomes by NHS organisations was also a barrier, particularly as we had based our sample size calculation on that, as opposed to the lower 7-day return rate.

The level of missing data for some variables in the routinely collected data, and not having data from which to take into account whether PA reduced the staff: patient ratio (or fully replaced FY2 doctors-in-training) is a further limitation and needs to be borne in mind in the comparisons we present. Likewise, our observation data illustrated care is predominently delivered by teams which creates difficulties in attributing outcomes or processes to individual staff, and compromised our ability to undertake an economic evaluation.

Our interview invitations yielded relatively small numbers of particpants, particularly amongst patients/relatives. While we attribute this in part to the fast patient throughput of the ED and limited availability of the researcher, this limits our analysis

Implications for policy and practice

PAs in the ED are acceptable to patients and can help to relieve staffing pressures and improve efficiency in the delivery of care. They are able to treat patients safely with a range of conditions and FY2 doctors-in-training deliver similar care to that provided by doctors in their second year of training. Deployment of PAs within ED teams is a potential solution to the situation of growing patient demand and predicted shortage of junior doctors in the British NHS[7], of which FY2 doctors on rotation in specialties such as the ED are one part; it is not our intention to raise or limit PAs to one particular junior doctor comparator level, but we have used this here as the closest pragmatic comparator. An alternative, which is to hire locum doctors, comes at a higher costs and loss of team continuity, and has potential implications for patient safety. Moves to regulate the PA profession under the General Medical Council were started in 2019.[36]

The findings of this study support employment of appropriately trained, supervised PAs with professional registration in ED teams. Further research is needed to investigate fully the impacts we have observed, particularly the cost effectiveness.

For peer review only - http://bmjopen.bmj.com/site/about/guidelines.xhtml

References

1. Bodenheimer, T. and Sinsky, C., 2014. From triple to quadruple aim: care of the patient requires care of the provider. The Annals of Family Medicine, 12(6), pp.573-576.
2. World Health Organization. Task Shifting: Rational Redistribution Of Tasks Among Health Workforce Teams – Global Recommendations and Guidelines. Geneva: World Health Organization; 2008. URL: www.who.int/healthsystems/task_shifting/en (last accessed 8-11-2019)
3. Reiter M, Wen LS, Allen BW. The Emergency Medicine Workforce: Profile and Projections. J Emerg Med. 2016 Apr;50(4):690-3. doi: 10.1016/j.jemermed.2015.09.022. Epub 2016
4. Takagi, K. and Tagami, T., 2019. Work-style reform of emergency physicians: the Japanese experience. European Journal of Emergency Medicine, 26(6), pp.398-399.
5. Suriyawongpaisal P, Atiksawedparit P, Srithamrongsawad S, Thongtan T. Assessment of nationwide emergency systems in Thailand, a middle-income country setting with UHC. Int J Health Plann Manage. 2019 Apr;34(2):e1346-e1355. doi: 10.1002/hpm.2783. Epub 2019 Apr 4.
6. Sinclair D, Abu-Laban RB, Toth P, LeBlanc C, Eisener-Parsche P, Frank JR, Holroyd B.Emergency Medicine Training and Practice in Canada: Celebrating the Past & Evolving for the Future. Collaborative Working Group on the Future of Emergency Medicine in Canada CJEM. 2017 Jul;19(S2):S1-S8. doi: 10.1017/cem.2017.372.
7. The Royal College of Emergency Medicine and the NHS. Securing the future workforce for emergency departments in England. October 2017 https://improvement.nhs.uk/documents/1826/Emergency_department_workforce_plan_-_111017_Final.3.pdf Accessed July 31, 2019.
8. Hall MK, Burns K, Carius M et al. State of the National Emergency Department Workforce: Who Provides Care Where? *Ann Emerg Med*. 2018;72:302-307.
9. Gardner G, Gardner A, Middleton S et al. Mapping workforce configuration and operational models in Australian emergency departments: a national survey. *Austr Health Rev*. 2018;42:340-7.
10. Thrasher C, Purc-Stephenson R.J. Patient satisfaction with nurse practitioner care in emergency departments in Canada. *Am Acad Nurse Pract*. 2008;20:231-7.
11. Ritsema T. The Faculty of Physician Associates Census Results 2018. Faculty of Physician Associates at the Royal College of Physicians. http://www.fparcp.co.uk/about-fpa/fpa-census Updated date unknown. Accessed September 10, 2019.
12. Cawley JF, Hooker RS. Determinants of the physician assistant/associate concept in global health systems. *International Journal of Healthcare*. 2018;4:50-60.
13. Farmer J, Currie M, Hyman J et. al. Evaluation of physician assistants in National Health Service Scotland. *Scott Med J*. 2011;56:130-4.

For peer review only - http://bmjopen.bmj.com/site/about/guidelines.xhtml

14. Close B, Zolcinski R. Physician assistants in Australasian emergency departments. *Emerg Med Australas*. 2016;28:475-477.

15. Howie N. A new kid on the block: the role of physician associates. Clinical Medicine; 2015; 14, 6: 691–4 DOI: 10.7861/clinmedicine.15-3-309a ·

16. Doan Q, Sabhaney V, Kissoon N, et al. A systematic review: The role and impact of the physician assistant in the emergency department. *Emerg Med Australas*. 2011;23:7-15.

17. Halter M, Wheeler C, Pelone F, et al. Contribution of physician assistants/associates to secondary care: a systematic review. BMJ Open. 2018 8:e019573. doi: 10.1136/bmjopen-2017-019573.

18. Creswell JW, Clark VL. Designing and Conducting Mixed Methods Research. Thousand Oaks, CA: Sage Publications Ltd; 2007.

19. Drennan VM, Halter M, Wheeler C, et al. What is the contribution of physician associates in hospital care in England? A mixed methods, multiple case study *BMJ Open* 2019;9 e027012.

20. NHS Digital Provisional Accident and Emergency Quality Indicators - England, by provider for April 2018. Accessed 2-8-2018 https://digital.nhs.uk/data-and-information/publications/statistical/accident-and-emergency-quality-indicators/april-2018-by-provider

21. Mackway Jones K, Marsden J, Windle J. Emergency Triage: Manchester Triage Group, Third Edition . 2013. London: John Wiley & Sons.

22. Sakr M, Angus J, Perrin J, Nixon C, Nicholl J, Wardrope J. Care of minor injuries by emergency nurse practitioners or junior doctors: a randomised controlled trial. *Lancet* 1999;354:1321-6.

23. Cooper MA, Lindsay GM, Kinn S, Swann IJ. Evaluating Emergency Nurse Practitioner services: a randomized controlled trial. *J Adv Nurs*. 2002.40:721-30.

24. Pope C, Mays N. Observational Methods. In Pope C, Mays N, editors. Qualitative Research in Health Care. 4th edn. Chichester: John Wiley & Sons; 2008. pp. 32–42.

25. Kvale S. Doing Interviews. London: Sage Publications; 2007. https://doi.org/10.4135/9781849208963

26. McCann, L, Granter E, Hyde P, Hassard, J. Still Blue-Collar after all these Years? An Ethnography of the Professionalization of Emergency Ambulance Work. *Journal of Management Studies* 2013;50:750–776.

27. Boyatzis RE. *Transforming qualitative information: Thematic analysis and code development.* Thousand Oaks, Calif: Sage;1998.

28. Maxfield RG. Use of physician's assistants in a general surgical practice. The American Journal of Surgery. 1976;131:504–508. doi: 10.1016/0002-9610(76)90165-3.

29. Rodi SW, Grau MV, Orsini CM. Evaluation of a fast track unit: alignment of resources and demand results in improved satisfaction and decreased length of stay for emergency department patients. Quality Management in Healthcare. 2006;15:163–170. doi: 10.1097/00019514-200607000-00006.

For peer review only - http://bmjopen.bmj.com/site/about/guidelines.xhtml

30. Berg GM, Crowe RE, Nyberg S, Burdsal C. Trauma patient satisfaction with physician assistants: testing a structural equation model. Journal of the American Academy of PAs. 2012;25(5):42–43.
31. Fréchette D, Shrichand A. Insights into the physician assistant profession in Canada. *JAAPA* 2016;29:35–9.
32. Kartha A, Restuccia JD, Burgess JF, Benzer J, Glasgow J, Hockenberry J, et al. Nurse practitioner and physician assistant scope of practice in 118 acute care hospitals. *J Hosp Med* 2014;9:615–20.
33. Timmermans MJ, van Vught AJ, Maassen IT, Draaijer L, Hoofwijk AG, Spanier M, et al. Determinants of the sustained employment of physician assistants in hospitals: a qualitative study. *BMJ Open* 2016;6:e011949.
34. Williams K. Advanced practitioners in emergency care: a literature review. *Emergency Nurse* 2017;25:36-41.
35. Hinson JS, Martinez DA, Cabral S, et al. Triage Performance in Emergency Medicine: A Systematic Review. *Annals Emerg Med*; 2018;S0196-0644(18)31282-4.
36. General Medical Council. GMC to regulate two new associates roles. Press release 18-July-2019. https://www.gmc-uk.org/news/news-archive/gmc-to-regulate-two-new-associates-roles---pas-and-aas Accessed July 2019.

review only

For peer review only - http://bmjopen.bmj.com/site/about/guidelines.xhtml

Supplementary material

Supplementary material 1: Extract of the chart review dataset

Supplementary material 2: Proforma for the assessment of clinical adequacy

Supplementary material 3: Observation guide

Supplementary material 4: Tailored topic guides for the interview

Author Statement

VMD (PhD, health policy and service delivery research), MH (PhD, health services research), JP (MD, general practice and clinical education), HG (PhD, health economics), SdeL (MD[Res], general practice and information science), JG (PhD, medical sociology), SB (BSc, patient and public engagement), and PB (PhD, audiology and strategic management) conceived and designed the study and obtained research funding. VMD, MH, JP supervised the conduct of the study and data collection. VMD, MH, JP undertook recruitment of participating centers and managed the data, including quality. CWa (PhD, statistics) undertook the statistical analysis; CW (PhD, health services research), LN (PhD,health services research), MH, JE (MSc, physician associate and education) and VMD undertook qualitative data collection and thematic analysis and HG considered the economic aspects. MH drafted the manuscript, and all authors contributed substantially to its revision. VMD takes responsibility for the paper as a whole.

All authors attest to meeting the four ICMJE.org authorship criteria: (1) Substantial contributions to the conception or design of the work; or the acquisition, analysis, or interpretation of data for the work; AND (2) Drafting the work or revising it critically for important intellectual content; AND (3) Final approval of the version to be published; AND (4) Agreement to be accountable for all aspects of the work in ensuring that questions related to the accuracy or integrity of any part of the work are appropriately investigated and resolved.

The Patient and Public Involvement statement

The patient and public voice was important to this study and informed the design, conduct, analsyis, interpretation and final reporting. We brought the views of our public and patient representative forum for a previous study on physician associates into the research questions and design of the study. These were views such as how do patients understand this new role. Sally Brearley, as a public voice representative, was a co-applicant and member of the research team. The study advisory group had two public voice members who were reimbursed for their time, following NIHR INVOLVE guidance. Two patient and public voice groups were formed: one in London and the other in the West Midlands and members reimbursed as per NIHR Involve guidelines. The patient and public voice groups

For peer review only - http://bmjopen.bmj.com/site/about/guidelines.xhtml

informed the design of the research tools such as topic guides and participant information sheets, developed coding frameworks and analysed interview transcripts , and participated in the overall synthesis of findings. Sally Brearley continues to be involved in the dissemination of the study.

Acknowledgements

Our thanks to all those clinicians, administrative and information staff in the participating centers who assisted the study at a time of heightened workload within the emergency services in the National Health Service in England. Our thanks also to Robert Grant who provided statistical advice in the design of the study and obtained research funding but left the research team before the data were obtained for analysis.

Data statement

No additional data are available.

For peer review only - http://bmjopen.bmj.com/site/about/guidelines.xhtml

Supplementary file 1: Extract of the chart review dataset (first 50 cases, according to date of attendance)

study_id	sample_case	site_numb	age_formatted	sex_formatted	arrival_date	arrival_time	seen_time	presenting_complaint	mts	ews	ed_stream	profession	xrays_form	pxn_forma	diagnosis	discharge_time	destination_gro	discharge_	discharge_	discharge_	reattend_	reattend_date
CS30115	0	3	5	0	05-Aug-16	9:10:00 PM	10:56:00 PM	Flank Pain(renal colic 3yrs ago)	#NULL!	#NULL!	1	1	#NULL!	#NULL!	Renal colic	2:07:00 AM	#NULL!	#NULL!	#NULL!		#NULL!	#NULL!
CS30197	0	3	6	1	05-Aug-16	7:54:00 PM	10:01:00 PM	Abdo/Back Pain 5/7	3	#NULL!	#NULL!	1	#NULL!	#NULL!	Generally unwell	1:40:00 AM	#NULL!	#NULL!	#NULL!		#NULL!	#NULL!
CS30415	0	3	4	1	05-Aug-16	4:51:00 PM	7:05:00 PM	21/40 ?DVT on clexane	3	#NULL!	#NULL!	1	#NULL!	#NULL!	Acute coronary syndrome	8:50:00 PM	#NULL!	#NULL!	#NULL!		#NULL!	#NULL!
CS30514	0	3	5	1	05-Aug-16	1:08:00 PM	2:15:00 PM	swelling to arm/hand ?clot	3	#NULL!	#NULL!	1	#NULL!	#NULL!	Deep venous thrombosis	5:07:00 PM	#NULL!	#NULL!	#NULL!		#NULL!	#NULL!
CS30726	0	3	3	0	05-Aug-16	11:45:00 PM	1:12:00 AM	HEADACHE	2	#NULL!	1	1	#NULL!	#NULL!	Unknown problem	1:58:00 AM	#NULL!	#NULL!	#NULL!		#NULL!	#NULL!
CS30962	0	3	4	0	05-Aug-16	4:26:00 PM	6:59:00 PM	Back Pain(mr maurice bulging disc)	3	#NULL!	#NULL!	0	#NULL!	#NULL!	Chronic back pain	8:20:00 PM	#NULL!	#NULL!	#NULL!		#NULL!	#NULL!
CS30993	0	3	4	1	05-Aug-16	6:57:00 AM	8:04:00 AM	Epigastric Pain 13months on/off	4	#NULL!	#NULL!	1	#NULL!	#NULL!	Acute gastritis	10:54:00 AM	#NULL!	#NULL!	#NULL!		#NULL!	#NULL!
CS31043	0	3	9	1	05-Aug-16	10:49:00 PM	12:00:00 AM	AP ?UTI	4	#NULL!	#NULL!	1	#NULL!	#NULL!	UTI - Urinary tract infection	2:13:00 AM	#NULL!	#NULL!	#NULL!		#NULL!	#NULL!
CS31165	0	3	3	1	05-Aug-16	9:56:00 PM	11:31:00 PM	PV Bleed+cramps(bloods with gp nad)	4	#NULL!	#NULL!	1	#NULL!	#NULL!	Menorrhagia	1:56:00 AM	#NULL!	#NULL!	#NULL!		#NULL!	#NULL!
CS31275	0	3	8	1	05-Aug-16	8:55:00 PM	10:50:00 PM	epigastric pain	3	#NULL!	1	0	#NULL!	#NULL!		12:38:00 AM	#NULL!	#NULL!	#NULL!		#NULL!	#NULL!
CS31564	0	3	1	0	05-Aug-16	4:26:00 PM	5:48:00 PM	not passing urine/feeding unsettled	3	#NULL!	#NULL!	0	#NULL!	#NULL!	Well child	6:52:00 PM	#NULL!	#NULL!	#NULL!		#NULL!	#NULL!
CS31572	0	3	3	0	05-Aug-16	11:20:00 PM	12:43:00 AM	Sickle cell attack	3	#NULL!	#NULL!	1	#NULL!	#NULL!	Sickle cell anemia crisis	3:18:00 AM	#NULL!	#NULL!	#NULL!		#NULL!	#NULL!
CS31639	0	3	8	1	05-Aug-16	8:53:00 AM	9:38:00 AM	HI, unwitnessed, stumbling on street	3	#NULL!	#NULL!	1	#NULL!	#NULL!	Head injury	12:50:00 PM	#NULL!	#NULL!	#NULL!		#NULL!	#NULL!
CS31640	0	3	8	1	05-Aug-16	10:24:00 AM	10:33:00 AM	FALL	3	#NULL!	#NULL!	1	#NULL!	#NULL!	Fall	2:23:00 PM	#NULL!	#NULL!	#NULL!		#NULL!	#NULL!
CS31641	0	3	3	1	05-Aug-16	10:46:00 AM	11:39:00 AM	FEELING FAINT	4	#NULL!	#NULL!	1	#NULL!	#NULL!	Fainting	2:03:00 PM	#NULL!	#NULL!	#NULL!		#NULL!	#NULL!
CS31642	0	3	1	0	05-Aug-16	3:04:00 PM	3:55:00 PM	unwell vomiting yellow	3	#NULL!	#NULL!	1	#NULL!	#NULL!	Vomiting - bile stained	6:01:00 PM	#NULL!	#NULL!	#NULL!		#NULL!	#NULL!
CS31644	0	3	8	1	05-Aug-16	8:17:00 PM	10:06:00 PM	abdo pain	#NULL!	#NULL!	1	1	#NULL!	#NULL!	Gastroenteritis	12:17:00 AM	#NULL!	#NULL!	#NULL!		#NULL!	#NULL!
CS32315	0	3	4	1	05-Aug-16	9:35:00 PM	11:24:00 PM	headaches	3	#NULL!	#NULL!	1	#NULL!	#NULL!	Headache	2:49:00 PM	#NULL!	#NULL!	#NULL!		#NULL!	#NULL!
CS32354	0	3	2	1	05-Aug-16	8:43:00 PM	10:24:00 PM	early pregnant, abdominal pain	3	#NULL!	#NULL!	1	#NULL!	#NULL!	Abdominal pain	12:18:00 AM	#NULL!	#NULL!	#NULL!		#NULL!	#NULL!
CS32524	0	3	9	1	05-Aug-16	2:48:00 PM	3:46:00 PM	FALL unwitnessed, unsteady (dementia)	3	#NULL!	#NULL!	1	#NULL!	#NULL!	Fall - accidental	6:44:00 PM	#NULL!	#NULL!	#NULL!		#NULL!	#NULL!
CS32671	0	3	6	0	05-Aug-16	3:10:00 PM	4:02:00 PM	?SEIZURE, hand was twitching (ca brain)	3	#NULL!	#NULL!	0	#NULL!	#NULL!	Partial seizure	7:10:00 PM	#NULL!	#NULL!	#NULL!		#NULL!	#NULL!
CS32693	0	3	8	1	05-Aug-16	10:20:00 PM	11:23:00 PM	RUQ Pain 3/7	4	#NULL!	1	1	#NULL!	#NULL!	Abdominal pain	2:19:00 AM	#NULL!	#NULL!	#NULL!		#NULL!	#NULL!
CS32713	0	3	9	0	05-Aug-16	4:09:00 PM	4:37:00 PM	UNWELL(bradycardia atropine given)	3	#NULL!	#NULL!	0	#NULL!	#NULL!	Acute confusion	8:09:00 PM	#NULL!	#NULL!	#NULL!		#NULL!	#NULL!
CS32840	0	3	7	0	05-Aug-16	11:06:00 AM	12:02:00 PM	SOB/Right sided CP (PE, pneumonia)	3	#NULL!	#NULL!	1	#NULL!	#NULL!	Pulmonary embolism	3:07:00 PM	#NULL!	#NULL!	#NULL!		#NULL!	#NULL!
CS32973	0	3	6	0	05-Aug-16	6:32:00 PM	9:01:00 PM	Paraphimosis	4	#NULL!	#NULL!	1	#NULL!	#NULL!	Paraphimosis	10:02:00 PM	#NULL!	#NULL!	#NULL!		#NULL!	#NULL!
CS33011	0	3	8	1	05-Aug-16	6:52:00 PM	8:55:00 PM	Suicidal/Intoxicated (etoh)	#NULL!	#NULL!	1	1	#NULL!	#NULL!	Alcohol intoxication (disorder)	10:51:00 PM	#NULL!	#NULL!	#NULL!		#NULL!	#NULL!
CS33793	0	3	7	1	05-Aug-16	2:25:00 PM	3:14:00 PM	COLLAPSED/AP ?seizure	3	#NULL!	#NULL!	1	#NULL!	#NULL!	Small bowel obstruction	6:25:00 PM	#NULL!	#NULL!	#NULL!		#NULL!	#NULL!
CS33926	0	3	7	1	05-Aug-16	11:44:00 AM	12:45:00 PM	painfull red eye]	2	#NULL!	#NULL!	1	#NULL!	#NULL!	Red eye	3:05:00 PM	#NULL!	#NULL!	#NULL!		#NULL!	#NULL!
CS34018	0	3	8	0	05-Aug-16	11:05:00 PM	12:34:00 AM	?UTI	3	#NULL!	#NULL!	1	#NULL!	#NULL!	Urinary tract infection	2:57:00 AM	#NULL!	#NULL!	#NULL!		#NULL!	#NULL!
CS34076	0	3	3	1	05-Aug-16	6:35:00 PM	8:54:00 PM	? chronns flare up/black stools	4	#NULL!	#NULL!	1	#NULL!	#NULL!	Crohn's disease (disorder)	10:34:00 PM	#NULL!	#NULL!	#NULL!		#NULL!	#NULL!
CS31018	1	3	9	1	05-Aug-16	6:26:00 PM	8:19:00 PM	SOB(inpatient langley green)	4	#NULL!	#NULL!	0	1	#NULL!	Pneumonia	4:57:00 AM	1	1.00	########	SPR	0	#NULL!
CS31643	1	3	5	0	05-Aug-16	8:03:00 PM	10:05:00 PM	Flank Pain(renal colic)	3	0	1	0	0	#NULL!	Left flank pain	11:13:00 PM	0	1.00	########	Consutlan	0	#NULL!
CS32456	1	3	7	0	05-Aug-16	7:27:00 PM	9:39:00 PM	sudden onset pain L testis	4	0	#NULL!	0	0	#NULL!	O/E - testicular swelling	11:23:00 PM	0	1.00	########	Consutlan	0	#NULL!
CS30038	0	3	7	0	06-Aug-16	7:45:00 PM	9:01:00 PM	2/7 hx CCP	3	#NULL!	#NULL!	1	#NULL!	#NULL!	Community acquired pneumonia	9:46:00 PM	#NULL!	#NULL!	#NULL!		#NULL!	#NULL!
CS30156	0	3	3	1	06-Aug-16	1:19:00 AM	3:08:00 AM	Headache took 24xParacetamol 500mg+4 kalms	2	#NULL!	1	1	#NULL!	#NULL!	Paracetamol overdose	7:39:00 AM	#NULL!	#NULL!	#NULL!		#NULL!	#NULL!
CS30320	0	3	5	0	06-Aug-16	1:55:00 AM	4:00:00 AM	Assault HI/Lft shoulder pain	4	#NULL!	1	1	#NULL!	#NULL!	Assault	5:55:00 AM	#NULL!	#NULL!	#NULL!		#NULL!	#NULL!
CS30351	0	3	3	1	06-Aug-16	2:02:00 AM	4:16:00 AM	Ear Infection(flucloxacillin)	4	#NULL!	#NULL!	1	#NULL!	#NULL!	Otitis media	5:25:00 AM	#NULL!	#NULL!	#NULL!		#NULL!	#NULL!
CS30392	0	3	7	0	06-Aug-16	7:08:00 PM	8:58:00 PM	R flank pain	4	#NULL!	#NULL!	1	#NULL!	#NULL!	Renal colic	10:24:00 PM	#NULL!	#NULL!	#NULL!		#NULL!	#NULL!
CS30398	0	3	3	0	06-Aug-16	7:00:00 PM	8:58:00 PM	?collapse - been drinking	3	#NULL!	#NULL!	1	#NULL!	#NULL!	Drunk	10:43:00 PM	#NULL!	#NULL!	#NULL!		#NULL!	#NULL!
CS30458	0	3	9	0	06-Aug-16	11:49:00 PM	1:47:00 AM	chest pain	3	#NULL!	#NULL!	1	#NULL!	#NULL!	Stable angina (disorder)	3:49:00 AM	#NULL!	#NULL!	#NULL!		#NULL!	#NULL!
CS30695	0	3	6	0	06-Aug-16	9:49:00 PM	12:02:00 AM	RTC 14hrs ago lower back pain,headache	4	#NULL!	#NULL!	1	#NULL!	#NULL!	Back pain	12:44:00 AM	#NULL!	#NULL!	#NULL!		#NULL!	#NULL!
CS30834	0	3	8	0	06-Aug-16	11:19:00 PM	12:55:00 AM	non epileptic seizure	3	#NULL!	#NULL!	1	#NULL!	#NULL!	Chronic confusion	10:13:00 AM	#NULL!	#NULL!	#NULL!		#NULL!	#NULL!
CS31121	0	3	3	1	06-Aug-16	9:08:00 PM	11:14:00 PM	OD/Mental health	#NULL!	#NULL!	#NULL!	1	#NULL!	#NULL!	Self-discharge	12:26:00 AM	#NULL!	#NULL!	#NULL!		#NULL!	#NULL!
CS31205	0	3	3	1	06-Aug-16	2:22:00 AM	3:53:00 AM	Passing small amounts urine	#NULL!	#NULL!	1	1	#NULL!	#NULL!	Renal colic	5:37:00 AM	#NULL!	#NULL!	#NULL!		#NULL!	#NULL!
CS31252	0	3	2	1	06-Aug-16	2:29:00 AM	5:03:00 AM	smoked cigarette.feels funny	4	#NULL!	1	1	#NULL!	#NULL!	Cigarette consumption	5:42:00 AM	#NULL!	#NULL!	#NULL!		#NULL!	#NULL!
CS31358	0	3	4	1	06-Aug-16	8:00:00 PM	9:39:00 PM	FLANK PAIN, KIDNEY STONES	4	#NULL!	#NULL!	1	#NULL!	#NULL!	Flank pain (finding)	12:00:00 AM	#NULL!	#NULL!	#NULL!		#NULL!	#NULL!
CS31534	0	3	3	1	06-Aug-16	12:45:00 AM	2:08:00 AM	Epigastric Pain	4	#NULL!	1	1	#NULL!	#NULL!	Gastritis	4:39:00 AM	#NULL!	#NULL!	#NULL!		#NULL!	#NULL!
CS31607	0	3	3	1	06-Aug-16	1:41:00 AM	3:08:00 AM	Abdo/back Pain	4	#NULL!	#NULL!	1	#NULL!	#NULL!	Palpitations - fluttering	4:37:00 AM	#NULL!	#NULL!	#NULL!		#NULL!	#NULL!
CS31624	0	3	4	0	06-Aug-16	11:32:00 PM	12:55:00 AM	?Cellulitis/Ulcers Bilateral	2	#NULL!	#NULL!	1	#NULL!	#NULL!	Cellulitis	3:32:00 AM	#NULL!	#NULL!	#NULL!		#NULL!	#NULL!

For peer review only

Supplementary material 2, assessment of clinical adequacy questions

Excel spread sheet sent to each reviewer to complete for all records. One column for each record – reviewer to fill in id number

	Questions *	Insert study ID number from top of record<br>*Drop down response options followed by a cell for free text if appropriate*
1	Record of the patient's medical history	Appropriate<br>Or<br>Error or omission – but unlikely to have resulted in harm or different treatment<br>Or<br>An error or omission seen that caused a breach in normal guidelines and procedures that would have altered the patient's treatment<br>Or<br>An error or omission seen that resulted in significant probability that the patient might be harmed
	Free text on rationale or comment on response to item 1	
2	Examination of the patient	Appropriate<br>Or<br>Error or omission – but unlikely to have resulted in harm or different treatment<br>Or<br>An error or omission seen that caused a breach in normal guidelines and procedures that would have altered the patient's treatment<br>Or<br>An error or omission seen that resulted in significant probability that the patient might be harmed
	Free text on rationale or comment on response to item 2	
3	Request for radiography	Appropriate<br>Or<br>Error or omission – but unlikely to have resulted in harm or different treatment<br>Or<br>An error or omission seen that caused a breach in normal guidelines and procedures that would have altered the patient's treatment<br>Or<br>An error or omission seen that resulted in significant probability that the patient might be harmed
	Free text on rationale or comment on response to item 3	
4	Treatment plan and decision	Appropriate<br>Or<br>Error or omission – but unlikely to have resulted in harm or different treatment<br>Or<br>An error or omission seen that caused a breach in normal guidelines and procedures that would have altered the patient's treatment<br>Or<br>An error or omission seen that resulted in significant probability that the patient might be harmed

For peer review only - http://bmjopen.bmj.com/site/about/guidelines.xhtml

	Questions *	Insert study ID number from top of record<br>*Drop down response options followed by a cell for free text if appropriate*
	Free text on rationale or comment on response to item 4	
5	Treatment plan and decision reviewed by senior doctor	YES<br>Or<br>NO
6	Advice given	Appropriate<br>Or<br>Error or omission – but unlikely to have resulted in harm or different treatment<br>Or<br>An error or omission seen that caused a breach in normal guidelines and procedures that would have altered the patient's treatment<br>Or<br>An error or omission seen that resulted in significant probability that the patient might be harmed
	Free text on rationale or comment on response to item 6	
7	Follow-up	Appropriate<br>Or<br>Error or omission – but unlikely to have resulted in harm or different treatment<br>Or<br>An error or omission seen that caused a breach in normal guidelines and procedures that would have altered the patient's treatment<br>Or<br>An error or omission seen that resulted in significant probability that the patient might be harmed
	Free text on rationale or comment on response to item 7	
8	In your view what type of clinician attended this patient?	Doctor<br>Or<br>Physician associate<br>Or<br>Unable to decide
	Free text on rationale or comment on response to item 8	

* Review questions taken from Sakr et al study [17] comparing patients attended by advanced nurse practitioners with doctors in the ED.

For peer review only

Supplementary file 3: Observation guide

OBSERVATION: AIDE MEMOIRE for researchers

Our observation aims to support answering the four study research questions but specifically to provide data on impact on organisation of services, other team members working practices and team relationships.

We wish to be able to consider this in terms of:

- Acceptability -how do they appear to be viewed or treated by others?
- Appropriateness – how are they observed in terms of safety e.g. how do they check, how are they checked upon, how are they supervised?
- Equity - who receives the PA service; do any patient groups appear to be over represented?
- Efficiency - how do they appear to contribute to this? How are issues, such as prescribing, worked around?
- Effectiveness – are the outcomes of PA care or contribution to the team observed?

We are observing context, relationships and activities.

Conduct of the observation

- Put up approved notices of our observation activity in the study setting places advised by the clinical team
- Provide the PA with the approved script to inform patients/ patients' representatives to gain permission for the researcher's presence. Each patient is to be asked for permission.
- Researcher to maintain an unobtrusive presence
- Record observations in the ethnographic tradition - take detailed unstructured notes, bearing in mind the importance of capturing context, relationships and activities
- Record as much as possible at the time and add as soon as possible afterwards
- Length of observation to be pre-planned but also to allow for flexibility according to the PA's wishes, the demands of the clinical setting and researcher's length of focus
- Allow the PA to see the notes at any point

After the observation

- Add to the notes as soon as possible where detail was not able to be captured at the time
- Maintain a reflective diary associated with the observation conduct and analytical processes.
- Discuss the observation with local research team members to promote group understanding and consistency across researchers
- Transfer data into NVivo software.

For peer review only - http://bmjopen.bmj.com/site/about/guidelines.xhtml

Supplementary data file 4: Tailored topic guides for the interview

Topic guide for senior managers and clinicians

Topic areas

- Confirm the person's job role
- Ask them to describe their involvement with physician associate employment in the hospital to date
- Ask questions on the factors supporting the adoption of the employment of physician associates
- Ask questions on the factors inhibiting the employment of physician associates
- Questions on their views of physician associates' impact on (ask for examples):
  - Organisation of services
  - Patient experience and outcomes
  - Other staff
  - Costs
- Anything else they would like to say?

Interviewer to probe on all answers to ensure the meaning is clear (e.g. that is an interesting point , can you explain a bit more about it) and check for understanding (e.g. so can I check I have understood you correctly)…….

Interviewer to check for any routine management reports or data or evaluations that the hospital team would be willing to share with the researchers.

Thank them and ask if they would like to receive updates on the study and a final summary of the findings. If so could they please give contact details which will be kept separate from the interview data.

For peer review only

Topic guide for physician associate interviews

Topic areas

- Ask them to describe how long they have been a physician associate, how many posts, type and length as a physician associate
- Ask them to describe the work they undertake, with what type of medical/surgical team
- Ask about their supervising doctor and arrangements when they are not there
- Ask questions on their views of the factors supporting the adoption of the employment of physician associate in their experience

For peer review only - http://bmjopen.bmj.com/site/about/guidelines.xhtml

- Ask questions on their views of the factors inhibiting the employment of physician associate in their experience
- Ask how they have been received in the hospital as a new type of health professional?
- Ask how they explain to patients, family and staff – who they are and what a physician associate is

- Questions on their views of their, or other physician associates, impact on (ask for examples):
    - Organisation of services
    - Patient experience and outcomes
    - Other staff
    - Costs

- Anything else they would like to say?

Interviewer to probe on all answers to ensure the meaning is clear (e.g that is an interesting point , can you explain a bit more about it) and check for understanding (e.g. so can I check I have understood you correctly)…….

Interviewer to check for any routine management reports or data or evaluations that the hospital team would be willing to share with the researchers .

Thank them and ask if they would like to receive updates on the study and a final summary of the findings. If so could they please give contact details which will be kept separate from the interview data.

Topic guide for all other types of professionals/managers interviews

- Confirm the person's job role
- Ask them to describe their involvement with physician associate employment in the hospital to date
- Ask questions on their views of any factors supporting the adoption of the employment of physician associates in their experience
- Ask questions on their views of any factors inhibiting the employment of physician associates in their experience
- Ask their views as to how the PAs have been received in that service/team, and probe for any explanations

For peer review only - http://bmjopen.bmj.com/site/about/guidelines.xhtml

- Questions on their views of physician associates' impact on (ask for examples):
  - Organisation of services
  - Boundaries between the job roles of different types of professionals e.g. with nurses
  - Patient experience and outcomes
  - Other staff
  - Costs

- Anything else they would like to say?

Interviewer to probe on all answers to ensure the meaning is clear (e.g that is an interesting point , can you explain a bit more about it) and check for understanding (e.g. so can I check I have understood you correctly)…….

Interviewer to check for any routine management reports or data or evaluations that the hospital team would be willing to share with the researchers.

Thank them and ask if they would like to receive updates on the study and a final summary of the findings. If so could they please give contact details which will be kept separate from the interview data.

Topic guide for patient interviews

<u>Topic areas</u>

- Confirm the person is/has been a patient
- Ask them to outline the type of care they have been in receipt of without giving personal medical details e.g. in patient for x days
- Confirm the patient has met the physician associate
- Explore what sort of involvement the physician associate has had with them
- Ask them how they understand the role of the physician associate in the medical/surgical team
- Ask them how they found receiving care from a physician associate
- If they were to need similar medical or surgical care, would they be content to receive similar care from a physician associate in the future as they had this time (and can

For peer review only - http://bmjopen.bmj.com/site/about/guidelines.xhtml

they explain why) or would they prefer someone different? And if yes, can they explain why?

- Anything else they would like to say?

Interviewer to probe on all answers to ensure the meaning is clear (e.g. that is an interesting point , can you explain a bit more about it) and check for understanding (e.g. so can I check I have understood you correctly).

Thank them and ask if they would like to receive updates on the study and a final summary of the findings. If so could they please give contact details which will be kept separate from the interview data.

For peer review only

For peer review only - http://bmjopen.bmj.com/site/about/guidelines.xhtml

O'Cathain A, Murphy E, Nicholl J. The quality of mixed methods studies in health services research. J Health Serv Res Policy. 2008;13(2):92-98.

Box 1 Good Reporting of A Mixed Methods Study (GRAMMS)

GRAMMS criterion	Page in manuscript
(1) Describe the justification for using a mixed methods approach to the research question	8
(2) Describe the design in terms of the purpose, priority and sequence of methods	8
(3) Describe each method in terms of sampling, data collection and analysis	8-10
(4) Describe where integration has occurred, how it has occurred and who has participated in it	10
(5) Describe any limitation of one method associated with the present of the other method	18-19
(6) Describe any insights gained from mixing or integrating methods	18-19

For peer review only

No.	Topic	Item
	**Title and abstract**	
S1	Title	Concise description of the nature and topic of the study Identifying the study as qualitative or indicating the approach (e.g., ethnography, grounded theory) or data collection methods (e.g., interview, focus group) is recommended
S2	Abstract	Summary of key elements of the study using the abstract format of the intended publication; typically includes background, purpose, methods, results, and conclusions
	**Introduction**	
S3	Problem formulation	Description and significance of the problem/phenomenon studied; review of relevant theory and empirical work; problem statement
S4	Purpose or research question	Purpose of the study and specific objectives or questions
	**Methods**	
S5	Qualitative approach and research paradigm	Qualitative approach (e.g., ethnography, grounded theory, case study, phenomenology, narrative research) and guiding theory if appropriate; identifying the research paradigm (e.g., postpositivist, constructivist/ interpretivist) is also recommended; rationale[b]
S6	Researcher characteristics and reflexivity	Researchers' characteristics that may influence the research, including personal attributes, qualifications/experience, relationship with participants, assumptions, and/or presuppositions; potential or actual interaction between researchers' characteristics and the research questions, approach, methods, results, and/or transferability
S7	Context	Setting/site and salient contextual factors; rationale[b]
S8	Sampling strategy	How and why research participants, documents, or events were selected; criteria for deciding when no further sampling was necessary (e.g., sampling saturation); rationale[b]
S9	Ethical issues pertaining to human subjects	Documentation of approval by an appropriate ethics review board and participant consent, or explanation for lack thereof; other confidentiality and data security issues
S10	Data collection methods	Types of data collected; details of data collection procedures including (as appropriate) start and stop dates of data collection and analysis, iterative process, triangulation of sources/methods, and modification of procedures in response to evolving study findings; rationale[b]
S11	Data collection instruments and technologies	Description of instruments (e.g., interview guides, questionnaires) and devices (e.g., audio recorders) used for data collection; if/how the instrument(s) changed over the course of the study
S12	Units of study	Number and relevant characteristics of participants, documents, or events included in the study; level of participation (could be reported in results)
S13	Data processing	Methods for processing data prior to and during analysis, including transcription, data entry, data management and security, verification of data integrity, data coding, and anonymization/deidentification of excerpts
S14	Data analysis	Process by which inferences, themes, etc., were identified and developed, including the researchers involved in data analysis; usually references a specific paradigm or approach; rationale [b]
S15	Techniques to enhance trustworthiness	Techniques to enhance trustworthiness and credibility of data analysis (e.g., member checking, audit trail, triangulation); rationale[b]
	**Results/findings**	
S16	Synthesis and interpretation	Main findings (e.g., interpretations, inferences, and themes); might include development of a theory or model, or integration with prior research or theory
S17	Links to empirical data	Evidence (e.g., quotes, field notes, text excerpts, photographs) to substantiate analytic findings
	**Discussion**	
S18	Integration with prior work, implications, transferability, and contribution(s) to the field	Short summary of main findings; explanation of how findings and conclusions connect to, support, elaborate on, or challenge conclusions of earlier scholarship; discussion of scope of application/ generalizability; identification of unique contribution(s) to scholarship in a discipline or field
S19	Limitations	Trustworthiness and limitations of findings
	**Other**	
S20	Conflicts of interest	Potential sources of influence or perceived influence on study conduct and conclusions; how these were managed
S21	Funding	Sources of funding and other support; role of funders in data collection, interpretation, and reporting

[a]The authors created the SRQR by searching the literature to identify guidelines, reporting standards, and critical appraisal criteria for qualitative research; reviewing the reference lists of retrieved sources; and contacting experts to gain feedback. The SRQR aims to improve the transparency of all aspects of qualitative research by providing clear standards for reporting qualitative research.

[b]The rationale should briefly discuss the justification for choosing that theory, approach, method, or technique rather than other options available, the assumptions and limitations implicit in those choices, and how those choices influence study conclusions and transferability. As appropriate, the rationale for several items might be discussed together.

ACADEMIC MEDICINE

S1 p3 (MMR cannot fit in more detail in title)

S2 P3

S3 P7

S4 P7

S5 P8, 9

S6 P10

S7 P8

S8 P9-10

S9 P10

S10 P9-10

S11 P10 (refers to detail elsewhere)

S12 P11

S13 P10 (refers to detail elsewhere)

S14 P10 (refers to detail elsewhere)
S14 As above

S16 P11-17, embedded alongside quant
S17 As above

S18 P17-19

S19 P18-19

S20&21 P5

only

For peer review only

The RECORD statement: Checklist of items, extended from the STROBE statement, that should be reported in observational studies using routinely collected health data.

	Item Number	STROBE Items	RECORD Items	Page number
**Title and Abstract**				
	1	(a) Indicate the study's design with a commonly used term in the title or the abstract. (b) Provide in the abstract an informative and balanced summary of what was done and what was found.	RECORD 1.1: The type of data used should be specified in the title or abstract. When possible, the name of the databases used should be included. RECORD 1.2: If applicable, the geographic region and time frame within which the study took place should be reported in the title or abstract. RECORD 1.3: If linkage between databases was conducted for the study, this should be clearly stated in the title or abstract.	3
**Introduction**				
Background rationale	2	Explain the scientific background and rationale for the investigation being reported.		7
Objectives	3	State specific objectives, including any prespecified hypotheses.		7
**Methods**				
Study Design	4	Present key elements of study design early in the paper.		8
Setting	5	Describe the setting, locations, and relevant dates, including periods of recruitment, exposure, follow-up, and data collection.		8
Participants	6	*(a) Cohort study*: Give the eligibility criteria and the sources and methods of selection of participants. Describe methods of follow-up. *Case-control study*: Give the eligibility criteria and the sources and methods of case ascertainment and control selection. Give the rationale for the choice of cases and controls. *Cross-sectional study*: Give the eligibility criteria and the sources and methods of	RECORD 6.1: The methods of study population selection (such as codes or algorithms used to identify subjects) should be listed in detail. If this is not possible, an explanation should be provided. RECORD 6.2: Any validation studies of the codes or algorithms used to select the population should be referenced. If validation was conducted for this study and not published	8

For peer review only

	Item Number	STROBE Items	RECORD Items	Page number
		selection of participants. *(b) Cohort study*: For matched studies, give matching criteria and number of exposed and unexposed. *Case-control study*: For matched studies, give matching criteria and the number of controls per case.	elsewhere, detailed methods and results should be provided. RECORD 6.3: If the study involved linkage of databases, consider use of a flow diagram or other graphical display to demonstrate the data linkage process, including the number of individuals with linked data at each stage.	
Variables	7	Clearly define all outcomes, exposures, predictors, potential confounders, and effect modifiers. Give diagnostic criteria, if applicable.	RECORD 7.1: A complete list of codes and algorithms used to classify exposures, outcomes, confounders, and effect modifiers should be provided. If these cannot be reported, an explanation should be provided.	10, 12
Data sources/measurement	8	For each variable of interest, give sources of data and details of methods of assessment (measurement). Describe comparability of assessment methods if there is more than one group.		8,10
Bias	9	Describe any efforts to address potential sources of bias.		10
Study size	10	Explain how the study size was arrived at.		9
Quantitative variables	11	Explain how quantitative variables were handled in the analyses. If applicable, describe which groupings were chosen and why.		8,10
Statistical methods	12	(a) Describe all statistical methods, including those used to control for confounding. (b) Describe any methods used to examine subgroups and interactions. (c) Explain how missing data were addressed. (d) *Cohort study*: If applicable, explain how loss to follow-up was addressed. *Case-control study*: If applicable, explain how matching of cases and controls was addressed. *Cross-sectional study*: If applicable, describe analytical methods taking account of sampling strategy. (e) Describe any sensitivity analyses.		10
Data access and cleaning methods		N/A	RECORD 12.1: Authors should describe the extent to which the investigators had access to the database population used to create the study population. RECORD 12.2: Authors	8-9

For peer review only

	Item Number	STROBE Items	RECORD Items	Page number
			should provide information on the data cleaning methods used in the study.	
Linkage		N/A	RECORD 12.3: State whether the study included person-level, institutional-level, or other data linkage across two or more databases. The methods of linkage and methods of linkage quality evaluation should be provided.	8-9
**Results**				
Participants	13	(a) Report the numbers of individuals at each stage of the study (e.g., numbers potentially eligible, examined for eligibility, confirmed eligible, included in the study, completing follow-up, and analysed). (b) Give reasons for nonparticipation at each stage. (c) Consider use of a flow diagram.	RECORD 13.1: Describe in detail the selection of the persons included in the study (i.e., study population selection), including filtering based on data quality, data availability, and linkage. The selection of included persons can be described in the text and/or by means of the study flow diagram.	11, 12, 13
Descriptive data	14	(a) Give characteristics of study participants (e.g., demographic, clinical, and social) and information on exposures and potential confounders. (b) Indicate the number of participants with missing data for each variable of interest. (c) *Cohort study*: summarise follow-up time (e.g., average and total amount).		12
Outcome data	15	*Cohort study*: Report numbers of outcome events or summary measures over time. *Case-control study*: Report numbers in each exposure category or summary measures of exposure. *Cross-sectional study*: Report numbers of outcome events or summary measures.		13
Main results	16	(a) Give unadjusted estimates and, if applicable, confounder-adjusted estimates and their precision (e.g., 95% confidence interval). Make clear which confounders were adjusted for and why they were included. (b) Report category boundaries when continuous variables were categorized. (c) If relevant, consider translating estimates of relative risk into absolute risk for a meaningful time period.		13,14

For peer review only

	Item Number	STROBE Items	RECORD Items	Page number
Other analyses	17	Report other analyses done—e.g., analyses of subgroups and interactions and sensitivity analyses		15
**Discussion**				
Key results	18	Summarise key results with reference to study objectives.		17-18
Limitations	19	Discuss limitations of the study, taking into account sources of potential bias or imprecision. Discuss both direction and magnitude of any potential bias.	RECORD 19.1: Discuss the implications of using data that were not created or collected to answer the specific research question(s). Include discussion of misclassification bias, unmeasured confounding, missing data, and changing eligibility over time, as they pertain to the study being reported.	19
Interpretation	20	Give a cautious overall interpretation of results considering objectives, limitations, multiplicity of analyses, results from similar studies, and other relevant evidence.		19
Generalisability	21	Discuss the generalisability (external validity) of the study results.		18-19
**Other Information**				
Funding	22	Give the source of funding and the role of the funders for the present study and, if applicable, for the original study on which the present article is based.		5
Accessibility of protocol, raw data, and programming code		N/A	RECORD 22.1: Authors should provide information on how to access any supplemental information such as the study protocol, raw data, or programming code.	24

N/A, not applicable

For peer review only

BMJ Open

Comparing physician associates and foundation year two doctors in training undertaking emergency medicine consultations in England: a mixed methods study of processes and outcomes

Journal:	*BMJ Open*
Manuscript ID	bmjopen-2020-037557.R2
Article Type:	Original research
Date Submitted by the Author:	25-Jun-2020
Complete List of Authors:	Halter, Mary; Kingston University and St George's, University of London, Faculty of Health, Social Care and Education<br>Drennan, Vari; Kingston University Faculty of Health Social Care and Education, Centre for Health & Social Care Research<br>Wang, Chao; Kingston University Faculty of Health Social Care and Education,<br>Wheeler, Carly; Imperial College Healthcare NHS Trust, Centre for Medication Safety and Service Quality<br>Gage, Heather; University of Surrey, School of Economics<br>Nice, Laura; University of Birmingham,<br>de Lusignan, Simon; University of Oxford, Nuffield Department of Primary Care Health Sciences; University of Surrey, Department of Clinical and Experimental Medicine<br>Gabe, Jonathan; Royal Holloway University of London, School of Law and Social Science<br>Brearley, Sally; Kingston University and St George's, university of London, Faculty of Health, Social Care and Education<br>Ennis, James; University of Chester, Chester Medical School<br>Begg, Phil; Royal Orthopaedic Hospital, Executive<br>Parle, Jim; University of Birmingham, Institute of Clinical Sciences
**Primary Subject Heading**:	Emergency medicine
Secondary Subject Heading:	Health services research, Qualitative research
Keywords:	ACCIDENT & EMERGENCY MEDICINE, Organisation of health services < HEALTH SERVICES ADMINISTRATION & MANAGEMENT, Quality in health care < HEALTH SERVICES ADMINISTRATION & MANAGEMENT

SCHOLARONE™
Manuscripts

I, the Submitting Author has the right to grant and does grant on behalf of all authors of the Work (as defined in the below author licence), an exclusive licence and/or a non-exclusive licence for contributions from authors who are: i) UK Crown employees; ii) where BMJ has agreed a CC-BY licence shall apply, and/or iii) in accordance with the terms applicable for US Federal Government officers or employees acting as part of their official duties; on a worldwide, perpetual, irrevocable, royalty-free basis to BMJ Publishing Group Ltd ("BMJ") its licensees and where the relevant Journal is co-owned by BMJ to the co-owners of the Journal, to publish the Work in this journal and any other BMJ products and to exploit all rights, as set out in our licence.

The Submitting Author accepts and understands that any supply made under these terms is made by BMJ to the Submitting Author unless you are acting as an employee on behalf of your employer or a postgraduate student of an affiliated institution which is paying any applicable article publishing charge ("APC") for Open Access articles. Where the Submitting Author wishes to make the Work available on an Open Access basis (and intends to pay the relevant APC), the terms of reuse of such Open Access shall be governed by a Creative Commons licence – details of these licences and which Creative Commons licence will apply to this Work are set out in our licence referred to above.

Other than as permitted in any relevant BMJ Author's Self Archiving Policies, I confirm this Work has not been accepted for publication elsewhere, is not being considered for publication elsewhere and does not duplicate material already published. I confirm all authors consent to publication of this Work and authorise the granting of this licence.

For peer review only

Comparing physician associates and foundation year two doctors in training undertaking emergency medicine consultations in England: a mixed methods study of processes and outcomes

Corresponding author

Mary Halter

Faculty of Health, Social Care and Education, Kingston University St George's, University of London, Cranmer Terrace, London SW17 0RE, UK. m.halter@sgul.kingston.ac.uk 020 8725 0337

Co-authors

Vari M Drennan

Faculty of Health, Social Care and Education, Kingston University St George's, University of London, London, UK

V.Drennan@sgul.kingston.ac.uk

Chao Wang

Faculty of Health, Social Care and Education, Kingston University St George's, University of London, Cranmer Terrace, London, UK

C.Wang@sgul.kingston.ac.uk

Carly Wheeler

Centre for Medication Safety and Service Quality, Imperial College Healthcare NHS Trust, London, UK

carly.wheeler@gmail.com

Heather Gage

Surrey Health Economics Centre, Department of Clinical and Experimental Medicine, University of Surrey, Guildford, UK

h.gage@surrey.ac.uk

Laura Nice

Institute of Clinical Sciences, University of Birmingham, UK

L.Nice@bham.ac.uk

For peer review only

Simon de Lusignan
Nuffield Department of Primary Care Health Sciences, Oxford, UK
simon.delusignan@phc.ox.ac.uk

Jonathan Gabe PhD
School of Law and Social Science, Royal Holloway, University of London, Egham, UK
J.Gabe@rhul.ac.uk

Sally Brearley
Faculty of Health, Social Care and Education, Kingston University St George's, University of London, Cranmer Terrace, London, UK
S.Brearley@sgul.kingston.ac.uk

James Ennis
Chester Medical School, University of Chester, UK
j.ennis@chester.ac.uk

Philip Begg
The Royal Orthopaedic Hospital Birmingham, UK
philip.begg@nhs.net

Jim Parle
Institute of Clinical Sciences, University of Birmingham, UK
j.v.parle@bham.ac.uk

Keywords

Physician Assistants; physician associates; Emergency Service, Hospital; emergency department; medical education

Word count:
Abstract 300
Main text 3960

For peer review only

Comparing physician associates and foundation year two doctors in training undertaking emergency medicine consultations in England: a mixed methods study of processes and outcomes

ABSTRACT

Objectives

To compare the contribution of physician associates to the processes and outcomes of emergency medicine consultations to that of foundation year two doctors-in-training.

Design

Mixed methods study: retrospective chart review using four months' anonymised clinical record data of all patients seen by physician associates or foundation year two doctors-in-training in 2016; review of a sub sample of 40 records for clinical adequacy; semi-structured interviews with staff and patients; observations of physician associates.

Setting

Three emergency departments in England

Participants

The records of 8816 patients attended by six physician associates and 40 foundation year two doctors-in-training; of these n=3197 had the primary outcome recorded (n=1129 PA, n=2068 doctor); 14 clinicians and managers and six patients or relatives for interview; five phyisican associates for observation.

Primary and secondary outcome measures

The primary outcome was unplanned re-attendance at the same emergency department within seven days. Secondary outcomes: consultation processes, clinical adequacy of care, and staff and patient experience.

Results

Re-attendances within seven days (n=194 [6.1%]) showed no difference between physician associates and foundation year two doctors-in-training (OR 0.87 [95% CI 0.61, 1.24], p=0.437). If seen by a physician associate, patients were more likely receive an x-ray investigation (OR 2.10 [95% CI 1.72, 4.24)] , p<0.001), , after adjustment for patient characteristics, triage severity of condition and statistically significant clinician intra-class correlation. Clinical reviewers found almost all patients' charts clinically adequate. Physician associates were evaluated as assessing patients in a similar way to foundation year two doctors-in-training and providing continuity in the team. Patients were

For peer review only - http://bmjopen.bmj.com/site/about/guidelines.xhtml

positive about the care they had received from a physician associate, but had poor understanding of the role.

Conclusions

Physician associates in emergency departments in England treated patients with a range of conditions safely, and at a similar level to foundation year two doctors-in-training, providing clinical operational efficiencies.

For peer review only

Article summary

Strengths and limitations of this study

- This study provides a well-powered quantitative comparative analysis of the documented processes and outcomes of patient care by physician associates and foundaion year two doctors-in-training in three emergency departments in different parts of England.
- We believe this to be the first empirical study of the outcomes of care provided by UK-trained physician associates in the emergency department, and the first internationally to include interview and observation data.
- Patients' views have not been previously reported for physician associates in this setting.
- The low sensitivity of the emergency department triage system to identify conditions other than the most serious was a problem and impaired the study's ability to describe case mix fully.

The original protocol for the study

The protocol for the study is available at the funding body's website https://www.journalslibrary.nihr.ac.uk/programmes/hsdr/141926/#/

Funding

This project was funded by the National Institute for Health Research Health Services and Delivery Research Programme (project number 14/19/26). This paper presents independent research commissioned by the National Institute for Health Research (NIHR). The views and opinions expressed by authors in this publication are those of the authors and do not necessarily reflect those of the NHS, the NIHR, the Health Service and Delivery Research Programme or the Department of Health.

Competing Interests

SdeL was head of the Department of Clinical and Experimental Medicine until June 2019 at the University of Surrey, which launched a physician associate course in 2016. JP is the immediate past chair of the UK and Ireland Board for Physician Associate Education and immediate past director of the physician associate programme at the University of Birmingham. PB is honorary faculty at the University of Birmingham and has taught on the physician associate programme since 2008. JE taught part time on the University of Birmingham physician associate programme until 2020. VMD was a HS&DR Board Member in 2015.

For peer review only

Main text

Introduction

Health care systems internationally are challenged to ensure good patient outcomes, within financial constraints, as well as to attend to the work life of the workforce.[1] Health workforce shortages, particularly of doctors, are resulting in the development of advanced clinical practitioners or non-physician clinicians (NPCs), such as nurse practitioners (NPs) and physician assistants/associates (PAs) in many countries.[2] Numerous countries are experiencing rising patient demand for emergency services and concommittant shortages of doctors in emergency medicine.[3-7] This situation has led to the development of NPC roles in emergency departments (EDs) in many countries such as the United States (US),[8] Australia,[9] Canada,[10] and the United Kingdom (UK).[11] In the US, 25% (n=14,360) of all emergency medicine clinicians are NPCs, and 68% of these are PAs.[8]

PAs are trained in the medical model to take histories, diagnose illness, develop management plans and prescribe medications as agreed with their supervising physician. PAs have a fifty year history in the US and are a developing part of the workforce in some other countries such as Canada, the Netherlands and Germany.[12] The PA workforce is growing in the UK (where they are known as physician associates). In 2018 there was an estimated 600 qualified PAs with approximately 1000 graduating each year since then.[11] Their employment specialties include EDs,[11] where they are deployed in both the minor and the major illness or injury sections.[8]

Descriptive observations have been published concerning the positive contributions by US-trained PAs employed in EDs in the UK,[13] Australia and New Zealand,[14] and by UK-trained PAs in England.[15] Unlike in the US, PAs in these other countries cannot prescribe medicines or order ionising radiation. PAs in North American EDs are reported to be well accepted by other staff and patients, and reliable in assessing certain medical complaints and performing procedures.[16] No difference is reported between patients attended by a PA and those attended by a doctor for wound infection rates, or rate of revisit within 72 hours to a pediatric ED; but studies find less consistency in practice when analysing prescribing patterns, length of stay and wait times of physicians, PAs and NPs in the ED.[17] There is relatively little research evidence on their clinical effectiveness,[17] little quantitative evidence on outcomes from outside of the US and no qualitative evidence of how PAs deliver care in the ED. In this context our goal was to investigate the contribution of PAs to the processes and outcomes of emergency medicine consultations compared to that of foundation year two (FY2) doctors-in-training in EDs in English hospitals.

For peer review only - http://bmjopen.bmj.com/site/about/guidelines.xhtml

Methods

Study design

We conducted a pragmatic, mixed methods convergence study in which we compare and contrast and simultaneously interpret quantitative and qualitative data[18] in three EDs in England, with three components. We undertook a quantitative observational retrospective chart review of patient consultations by PAs compared with FY2 doctors-in-training; and qualitatively we directly observed PAs' practice; and we conducted semi-structured interviews with members of the staff team. Our planned prospective study of patient records with a linked patient satisfaction and outcomes survey had to be revised to a pragmatic retrospective chart review due to practicalities within the participating NHS organisations in the period of the study.

Population and sampling

Three consultant-led, 24 hour EDs with full resuscitation facilities ('type one') participated. Two EDs had annual attendances in the range of 100,000 – 120,000 adult and pediatric patients and the third in the range of 170,000 -190,000. One was an university hospital; two were district general hospitals. The hospitals had been recruited as part of a larger study investigating the work and contribution of PAs between 2016 and 2017.[19] We selected FY2 doctors-in-training as the comparator for PAs, as PAs are offered as part of a solution to junior medical workforce shortages[7] and the most junior doctors working in the UK ED at the time were FY2s.

Selection of participants, measurements and outcomes

Our primary outcome was unplanned re-attendance at the same ED within seven days - one of the NHS clinical quality indicators for EDs in England.[20] Our secondary outcomes were: consultation processes (length of time in the ED, use of x-ray, prescriptions and referrals); clinical adequacy of care, referrals and planned follow up; and patient experience.

Chart review

For a 16-week period (the standard duration of ED placement for FY2 doctors-in-training in the UK), we obtained anonymised, routinely-collected electronic records of all patients attended by a PA or FY2 doctor-in-training, provided in Microsoft Excel by the hospital information teams in each trust, using queries based on staff job role, dates and requested data items. Hospital staff extracted additional data items (supplementary material 1) – age, sex, acuity (as categorised by the Manchester

For peer review only - http://bmjopen.bmj.com/site/about/guidelines.xhtml

triage score [21]), x-ray orders, diagnosis, prescription issued, admission, area treated, overall time in the ED (from check in to discharge, in minutes), and re-consultation within seven days (the primary outcome). No data linkage was required. The researchers did not have access to the original data set and so could not identify if any patients appeared more than once in the dataset, and further data cleaning could not be performed.

We calculated a sample size for the primary outcome based on rate of 18.3% (the highest of two rates for nurse practitioners substituting for physicians, [at 28 days]).[22,23] Aiming to find a relative difference of 50%, in a non-inferiority hypothesis, we required 284 patients in each group (calculation from Stata v11.1 software) to compare 18.3% to 27.4% unplanned re-consultations, with conventional 80% power at 5% significance. We included an extra 20 to allow for adjustment for case mix, requiring a minimum of 304 patients in total in each group to achieve the said power. As 28 day data could not be collected we went on to use the seven day reattendance rate, with its national average of 7.4% (range 2.4% to 21.7%) for unplanned re-attendance at the same ED in England within for all patients.

Two of the participating EDs also agreed to take part in the analysis of clinical adequacy of documented care in every tenth case from the chart review sample (n=40), with equal numbers of cases seen by PA and FY2 doctors-in-training, and using the full anonymised clinical record. We recruited two specialty registrars (doctors in their sixth year of emergency medicine training), one PA lecturer with 20 years ED experience, and one emergency medicine consultant (with 17 years experience at consultant level) from outside the three study hospitals to review these records. All four clinicians independently recorded their judgement as to the clinical adequacy of care for each record using the categories of past medical history, examination, request for radiography, treatment plan and decision, advice given and follow up. Their assessments were blinded to the type of professional attending the patient and to each other's assessment, using a proforma (supplementary material 2) based on published studies.[22,23] As the senior clinician, we accepted the decision of the consultant in cases of disagreement.

Observation

This element drew on the ethnographic tradition used in many health service research studies.[24] We invited all PAs working in the ED in our three study hospitals to participate (n=6). Five PAs volunteered and gave written informed consent to be observed. One of three researchers (CWh, LN, MH) observed each PA for two or three pre-arranged sessions, of varying lengths, on weekdays in periods between 08.00 and 22.00, following a broad guide (supplementary material 3). Researchers made notes on context, relationships and activities following this guide.We judged data saturation to

For peer review only - http://bmjopen.bmj.com/site/about/guidelines.xhtml

have been reached with individual PAs when the processes of care observed did not differ significantly from previous observations. During the observation period, PAs asked for patient assent to the researcher's presence. Researchers reflected on the observations, discussing them in pairs.

Interview

Semi structured interviews [25] were undertaken with a purposive sample of managerial, medical and nursing ED staff who volunteered after receiving information about the study from the researcher during observation periods and/or via their site manager. We also opportunistically interviewed patients and/or their relative who were being seen by a PA in the ED, identified and invited to particpate during observation periods, once they had been assessed and treated by the PA but before discharge from the ED. We used tailored topic guides (supplementary material 4) to explore interviewees' perceptions of the PA role and its impact on service organization, role boundaries, patient experience, patient outcomes, and activities and attitudes of other staff. We digitally recorded interviews or took notes if the participant preferred. Recordings were transcribed verbatim and anonymised.

Analysis

Chart review: The characteristics of patients treated by PAs and FY2 doctors-in-training were compared using chi-squared tests. We carried out a logistic regression to examine whether the primary and binary secondary outcomes differed between PAs and FY2 doctors-in-training, while adjusting for confounding factors - patient age, sex and triage score. Since patients seen by the same clinician are likely to be correlated, we calculated intraclass correlation coefficients (ICC) for each outcome and report results using a random-effects model if the ICC is statistically significant. We report odds ratios, their confidence intervals (CI), and two-tailed p values. For length of stay, a linear regression was used for data transformed to logarithm scale to reduce heteroscedasticity and reflect the fact that the value of length of stay is positive. To account for unobserved heterogeneity, the unobserved component is modelled as a latent variable in a latent class linear model. The assessment of clinical adequacy is reported using descriptive statistics, sensitivity and specificity of the judgment of whether the record was that of a PA or FY2 doctor-in-training, and Fleiss kappa for inter-rater agreement, calculated for each of the four components of the assessment and per response.

Qualitative: Our methods for the analysis of observation data drew on methods to identify ethnographic vignettes.[26] We employed thematic analysis[27] of all-specialty interview data for the wider study. Both are described in full elsewhere.[19] For the subsequent specialty-specific analysis we re-read all ED observation data and interview transcripts to identify all data related to the primary and secondary outcomes, and which both confirmed or disconfirmed findings.

For peer review only - http://bmjopen.bmj.com/site/about/guidelines.xhtml

Mixed methods: Following the separate quantitative and qualitative analyses, we (MH and VMD, in consultation with all authors) merged [18] the quantitative and qualitative datasets by presenting the quantitative results by study outcomes and following these with qualitative data findings (themes and/or excerpts or quotes) that confirmed or disconfirmed the quantitative results.

Ethical approval

We gained approval from the NHS Health Research Authority London-Central Research Ethics Committee (15/LO/1339).

Results

Characteristics of chart review subjects

In the 16 week period studied, 8,816 patients seen by six PAs (n=2890) or forty FY2 doctors-in-training (n=5926) were identified; some secondary outcomes were available for all cases. For 3197 of these patient episodes (n=1129 by the six PAs and n= 2068 by 22 FY2 doctors-in-training) the primary outcome was collected at site for the research team. Characteristics of the patients are shown in Table 1. PAs saw a lower proportion of patients categorised on triage into the urgent category than FY2 doctors-in-training.

In interview, the type of patient seen, patient throughput and role of PAs and FY2 doctors-in-training were described as similar:

They're [the PAs] pretty much equal to .....a senior FY2 doctor in training level. As a consultant we feel comfort because we know [PA name 1] can work in majors, she can clear [majors] pretty much...... And [PA name 2],... can clear paeds minors... Participant 150 Emergency Medicine consultant

However more than one participant tentatively suggested that PAs saw the less acutely unwell patients.:

So my understanding is like they're [the PAs] equivalent to, I would put it like a certain level of like a junior physician.....I wouldn't say they would be at registrar level.....I'd put them somewhere in between. You know a...lot better than like a newly qualified physician because they've got the skills and stuff, so in that gap of what I would say equivalent to maybe like a second to four years post qualified doctor. Participant 144, Registrar

For peer review only - http://bmjopen.bmj.com/site/about/guidelines.xhtml

Characteristics of interview and observation participants

The staff interviewed included four PAs, two managers, five nurses and three senior doctors; six patients and/or their relatives were also interviewed, spread across the three sites. We observed four PAs, at three sites; we do not report further demographic details due to concerns about anonymity in a small population.

For peer review only

For peer review only - http://bmjopen.bmj.com/site/about/guidelines.xhtml

1 Table 1 Characteristics of chart review sample

Characteristic	PA (N = 2381) N (%)	FY2 doctor (N = 6435) N (%)	Total (N = 8816) N (%)	p-value
**Age band**				
0-20	300 (13.0%)	656 (10.3%)	956 (11.0%)	0.002
21-40	543 (23.5%)	1493 (23.5%)	2036 (23.5%)	
41-60	530 (22.9%)	1406 (22.1%)	1936 (22.3%)	
61-80	551 (23.8%)	1596 (25.1%)	2147 (24.7%)	
81 and over	390 (16.9%)	1212 (19.0%)	1602 (18.5%)	
Unknown				
**Sex**				
Male	1132 (47.5%)	2933 (45.6%)	4065 (46.1%)	0.102
Female	1249 (52.5%)	3501 (54.4%)	4750 (53.9%)	
Unknown				
**Manchester triage score**				
1 Immediate	10 (0.6%)	3 (0.1%)	13 (0.2%)	<0.001
2 Very urgent	163 (9.3%)	565 (11.1%)	728 (10.6%)	
3 Urgent	770 (43.8%)	2841 (55.7%)	3611 (52.6%)	
4 Standard	811 (46.1%)	1681 (32.9%)	2492 (36.3%)	
5 Non Urgent	5 (0.3%)	12 (0.2%)	17 (0.2%)	
Unknown				
**ED area treated in**				
Minor	369 (20.1%)	275 (6.8%)	644 (10.9%)	<0.001
Major	1266 (68.8%)	3601 (88.4%)	4867 (82.3%)	
Resuscitation	2 (0.1%)	4 (0.1%)	6 (0.1%)	
Paediatrics	181 (9.8%)	174 (4.3%)	355 (6.0%)	
Clinical decision unit or primary care	21 (1.1%)	20 (0.5%)	41 (0.7%)	
Unknown				

For peer review only - http://bmjopen.bmj.com/site/about/guidelines.xhtml

The primary outcome: rate of return to the ED within seven days

Re-attendance within seven days was found following 6.1% (N = 194) of the 3197 index visits for which these data were available. The high rate of unknown data is accounted by one site where these data were not captured in the electronic dataset and were only retrieved manually for a random sample (n=205) for the purposes of this study. After adjustment for confounding, no statistically significant difference was found for cases seen by PAs or FY2 doctors-in-training; see Table 2.

Table 2: Re-attendance at the same ED within seven days

**Re-attendance at the same ED within seven days**	PA (N = 1129)	FY2 doctor (N = 2068)	Total (N = 3197)	Unadjusted odds ratio (95% CI) and p-value (PA relative to FY2 doctor-in-training) in rate of re-attendance	Adjusted odds ratio (95% CI) and p-value (PA relative to FY2 doctor-in-training) in rate of re-attendancee†
No	1066 (94.4%)	1937 (93.7%)	3003 (93.9%)	0.87 (0.64, 1.19) p=0.388	0.87 (0.61, 1.24) p=0.437
Yes	63 (5.6%)	131 (6.3%)	194 (6.1%)		
Unknown	1251	4368	5619	-	-

†Adjustment made for triage score (as a measure of acuity), age band, sex, admission, x-ray and site; no adjustment was made for clustering as the ICC by individual staff member on outcome was small (0.008) and statistically insignificant (p= 0.236).

Secondary outcome: consultation processes

No differences were found between patients attended by PAs or by FY2 doctors-in-training in: whether prescriptions were given, admission to hospital from the ED, or if a discharge summary was completed. However, patients seen by a PA were more likely to have an x-ray performed in the ED (Table 3), less likely to be admitted to hospital, and to have a shorter length of stay in the ED (by 35 minutes), after adjustment for age, sex, acuity, whether admitted, x-ray taken, and site, as well as for clustering by individual clinician, although no account was able to be taken of the staffing level.

We observed PAs being the first member of the medical team to carry out assessment of patients following triage to either the major, minor or paediatric areas of the ED. We noted that PAs saw patients independently, following a medical history taking and examination model, before reporting in

For peer review only

person to the senior ED physician in the same way as nurse practitioners and FY2 doctors-in-training did.

For peer review only

1 Table 3 Clinical process measures

Clinical process measure	PA (N = 2381)	FY2 doctor (N = 6435)	Total (N = 8816)	Unadjusted odds ratio (95% CI) and p-value (PA relative to FY2 doctor-in-training) in rate of re-attendance	Adjusted odds ratio (95% CI) and p-value (PA relative to FY2 doctor-in-training) in rate of re-attendance†
**X-ray investigations performed**					
No	559 (49.4%)	1701 (82.3%)	2260 (70.7%)	4.76 (4.04, 5.59) p<0.001	2.70 (1.72, 4.24) p<0.001
Yes	572 (50.6%)	366 (17.7%)	938 (29.3%)		
Unknown	1250	4368	5618	-	-
**Prescriptions given in the ED**					
No	174 (58.0%)	157 (51.8%)	331 (54.9%)	0.79 (0.56, 1.07) p=0.127	1.35 (0.08, 23.5) p=0.838
Yes	126 (42.0%)	146 (48.2%)	272 (45.1%)		
Unknown	2081	6132	8213		
**Admitted as an inpatient from the ED**					
No	883 (78.2%)	1436 (70.1%)	2319 (73.0%)	0.65 (0.55, 0.77) p<0.001	0.78 (0.55, 1.1) p=0.158
Yes	246 (21.8%)	613 (29.9%)	859 (27.0%)		
Unknown	1762	3876	5638		
**Discharge summary completed**					
No	86 (42.4%)	71 (34.6%)	157 (38.5%)	0.72 (0.48, 1.08) p=0.109	1.57 (0.93, 2.66) p=0.09
Yes	117 (57.6%)	134 (65.4%)	251 (61.5%)		
Unknown	2178	6230	8408		

2 †Adjustment made for MTS (as a measure of acuity), age band, sex, and site, and for clustering where the ICC
3 (and p-value) is significant: x-ray 0.04 (p<0.001), prescriptions 0.73 (p<0.001), admitted 0.02 (p=0.001),
4 discharge summary <0.001 (p=0.498)

5

6 PAs were differentiated from FY2 doctors-in-training by many of our interviewees for not being able
7 to prescribe medications or order tests utilising ionising radiation. Some participants considered this
8 to have a detrimental impact on PAs and patients:

9

For peer review only

[prescribing] would make a massive difference for them as well and [for] patients because at the end of the day they're having to wait for the PAs to go talk through [with] the physicians what's going on and then probably see somebody else. Participant 118 Nurse practitioner

However, PAs were observed taking on several roles in relation to prescriptions and x-ray orders, for example suggesting medications to or charting the medication for a senior doctor to sign off:

So when one of my PAs comes to me and says "This patient has a temperature of 38, they're coughing up horrible green sputum and they're tachycardic and I listened to their chest and they've got crackles at the left base, can we order a chest x-ray and prescribe sepsis drugs for, you know, pneumonia?" I say "Yes" and I sign it. With probably more confidence at this stage having had [number] PAs here for a year than I would with a junior physician in training on day two. And the irony of that is of course, the junior physician in training doesn't need to come and ask me, technically, they can prescribe themselves. Participant 21 Emergency Medicine consultant

PAs were also observed making referrals to medical and surgical teams outside of the ED, completing discharge summary information, and carrying out procedures, most commonly cannulation, phlebotomy and suturing.

Secondary outcome: clinical adequacy

Our reviewers found the chart documentation to have been 'appropriate' or 'with no errors or omissions that resulted in significant probability that the patient might be harmed' in 36/40 cases for all of the key consultation components (Table 4). In the three records (two of FY2 doctors-in-training and one of a PA) judged as having errors or omissions at the level of a breach in normal guidelines and procedures that would have altered the patient's treatment, all reviewers agreed that a senior doctor review had occurred in one case; this was unclear in the other cases. Our observation data suggest that such a senior review was undertaken for all assessment and clinical decision making in the 'majors' sections of the ED, but that 'minors' care was often completed independently.

Our reviewers were 40% sensitive, 46% specific on judging the clinician type: 68% (13/19) of the PA records were thought to be of a FY2 doctor-in-training and 60% (9/15) vice versa (kappa score for inter-rater agreement 0.15).

For peer review only

Table 4: Chart reviewers' assessments of clinical adequacy

**PA or FY2 consultation record**		**Judgment of appropriateness**																							
		**Past medical history**				**Examination**				**Request for radiography***				**Treatment plan and decision**				**Advice given**				**Follow up**			
		Appropriate	Error or omission			Appropriate	Error or omission			Appropriate	Error or omission			Appropriate	Error or omission			Appropriate	Error or omission			Appropriate	Error or omission		
			Harm unlikely	Altered treatment	Harm		Harm unlikely	Altered treatment	Harm		Harm unlikely	Altered treatment	Harm		Harm unlikely	Altered treatment	Harm		Harm unlikely	Altered treatment	Harm		Harm unlikely	Altered treatment	Harm
FY2	n	14	6	0	0	15	5	0	0	9	0	1	0	14	5	1	0	4	1	1	0	16	3	0	0
	%	70	37	0	0	75	25	0	0	45	0	5	0	70	25	5	0	20	5	5	0	80	15	0	0
PA	n	13	5	1	0	11	7	1	0	9	3	0	0	13	5	1	0	3	1	1	0	13	4	1	0
	%	65	25	5	0	55	35	5	0	45	15	0	0	65	25	5	0	15	5	5	0	65	20	5	0
Not rated*	n	1				1				18				1				29				3			
	%	2.5				2.5				45				2.5				73				7.5			
Total	n	27	11	1	0	26	12	1	0	18	3	1	0	27	10	2	0	7	2	2	0	29	7	1	0
	%	68	28	3	0	65	30	3	0	45	8	3	0	66	25	5	0	18	5	5	0	73	18	3	0
Agreement (Fleiss kappa, combined)		0.01				0.15				0.26				0.15				-0.03				0.30			
Agreement (Fleiss kappa, per response)		0.04	-0.02	-0.04	n/a	0.17	0.12	0.15	n/a	0.29	0.11	0.33	n/a	0.24	0.01	0.14	n/a	-0.00	0.11	0.08	n/a	0.36	0.20	0.28	n/a

*Missing rating or rated as 'not applicable' if no request for radiography was made or no advice given

For peer review only

Interviewees also presented other aspects related to clinical adequacy, particularly the PAs' stability in the team. The clinicians' familiarity with the longer standing team member PA/s - in contrast to FY2 doctors-in-training on rotation - was raised repeatedly:

> *If there was a junior physician over here, and he said oh, what do you think of this wound, which they do ask us. And I say yeah, it needs suturing. I then have to say, but can you suture or do you want me to suture it?.....Because I don't know, and some will say oh no, I can't.....I've never sutured before, and some will say oh yeah, that's fine, I'll suture it.....Whereas I know with PAs they'll suture their own. Because I know that they've got that skill set.* Participant 177 Advanced nurse practitioner

Secondary outcome: patient experience

Patients were positive about the care they had received from the PA, but had not understood what the PA role meant, with two participants believing they had been seen by a doctor and another unsure in the context of multiple ED staff:

> *I presumed he was a fully-qualified physician, yes his approach and everything was absolutely 100%.* Participant 120 Patient

Most of our patient participants were receptive to the role on the grounds that it might speed up care, although they were not without concern for the difference in training from a doctor and the diminishment of a senior medical workforce:

> *It's good to have another person, another opinion…but would it not perhaps be better to have another doctor?* Participant 083 Patient's relative

Discussion

Summary of findings

The study presents evidence from three English EDs and has demonstrated no difference in safety or appropriateness between PAs and FY2 doctors-in-training. We report no difference in re-attendance rates. Those patients seen by a PA (within PA working hours 08-22.00) had a shorter average length of stay in the ED than those seen by doctors-in-training (24 hour working period). Our review of clinical adequacy found few errors and no difference between PAs and FY2 doctors-in-training. Patients appeared relatively unconcerned with the title of the clinician treating them and thought they

For peer review only - http://bmjopen.bmj.com/site/about/guidelines.xhtml

had been treated by a doctor; however they were keen to know that the employment of PAs would not represent a widespread substitution for doctors in the ED.

How this study is similar or different from prior studies

We believe this to be the first empirical study of the outcomes of care provided by UK-trained PAs in the ED, and the first internationally to include interview and observation data. Additonally, patients' views do not appear to have been previously gathered at the time of the visit (and qualitaitvely), although there have been previous questionnaire studies in the USA of patient satisfaction, administered after the visit.[28,29,30]

We reported few differences in the the practice and processes of care – other than prescribing (which PAs currently cannot do independently in the UK) – between PAs and doctors in their second foundation year of training. Our finding of no difference in the primary outcome (ED reattendance rate within seven days) for patients of PAs and FY2 doctors-in-training is consistent with the comparisons of nurse-qualified NPCs and FY2 doctors-in-training on which we based our study design [22,23] and other PA literature from the USA.[16] It should be noted that for patients in the majors section of ED, all assessment and treatment plans by FY2 doctors-in-training and NPCs were reviewed and agreed by a senior clinician. Our participants commented frequently on the transient nature of FY2 doctors-in-training, whose rotation in the ED only last four months. In contrast, PAs remained long-term and provided continuity in the team. Their accumulated knowledge of the policies and practices (clinical and otherwise) of the department, the consultants and the hospital was reported to enable operational efficiencies. Simlar observations about PAs providing continuity within the medical/surgical team have been made in North America and the Netherlands[31-33] and also for other NPCs.[34]

This study's strengths lie in its mixed-methods approach to the study of PAs in the ED, allowing consideration of different types of data on their contribution, compared to that of FY2 doctors-in-training, to be considered. We were able to carry out a well-powered quantitative comparative analysis of the documented processes and outcomes of patient care by PAs and FY2 doctors-in-training in three EDs in different parts of the country, and to gather qualitative data on PAs 'in practice'. The qualitative component of our mixed methods approach enabled contextual explanations of the quantitative analysis.

Our study however has several limitations. Our comparison of PAs and doctors working in all areas of the ED introduced the potential for PAs and FY2 doctors-in-training to be attending to patients of different acuity and complexity. We sought to mitigate this by using three different EDs, taking a

For peer review only - http://bmjopen.bmj.com/site/about/guidelines.xhtml

sample across a 16 week period at all times of day and night (although the FY2 doctors-in-training worked over the 24 hour period when staff:patient ratios may have fluctuated). We also made statistical adjustments that included triage category. The low sensitivity of most ED triage systems to identification of conditions other than the most serious, however, is a drawback.[35] The prevention of collection of 28 day outcomes by NHS organisations was also a barrier, particularly as we had based our sample size calculation on that, as opposed to the lower 7-day return rate.

The level of missing data for some variables in the routinely collected data, and not having data from which to take into account whether PA reduced the staff: patient ratio (or fully replaced FY2 doctors-in-training) is a further limitation and needs to be borne in mind in the comparisons we present. Likewise, our observation data illustrated care is predominently delivered by teams which creates difficulties in attributing outcomes or processes to individual staff, and compromised our ability to undertake an economic evaluation.

Our interview invitations yielded relatively small numbers of particpants, particularly amongst patients/relatives. While we attribute this in part to the fast patient throughput of the ED and limited availability of the researcher, this limits our analysis

Implications for policy and practice

PAs in the ED are acceptable to patients and can help to relieve staffing pressures and improve efficiency in the delivery of care. They are able to treat patients safely with a range of conditions and FY2 doctors-in-training deliver similar care to that provided by doctors in their second year of training. Deployment of PAs within ED teams is a potential solution to the situation of growing patient demand and predicted shortage of junior doctors in the British NHS[7], of which FY2 doctors on rotation in specialties such as the ED are one part; it is not our intention to raise or limit PAs to one particular junior doctor comparator level, but we have used this here as the closest pragmatic comparator. An alternative, which is to hire locum doctors, comes at a higher costs and loss of team continuity, and has potential implications for patient safety. Moves to regulate the PA profession under the General Medical Council were started in 2019.[36]

The findings of this study support employment of appropriately trained, supervised PAs with professional registration in ED teams. Further research is needed to investigate fully the impacts we have observed, particularly the cost effectiveness.

For peer review only - http://bmjopen.bmj.com/site/about/guidelines.xhtml

References

1. Bodenheimer, T. and Sinsky, C., 2014. From triple to quadruple aim: care of the patient requires care of the provider. The Annals of Family Medicine, 12(6), pp.573-576.
2. World Health Organization. Task Shifting: Rational Redistribution Of Tasks Among Health Workforce Teams – Global Recommendations and Guidelines. Geneva: World Health Organization; 2008. URL: www.who.int/healthsystems/task_shifting/en (last accessed 8-11-2019)
3. Reiter M, Wen LS, Allen BW. The Emergency Medicine Workforce: Profile and Projections. J Emerg Med. 2016 Apr;50(4):690-3. doi: 10.1016/j.jemermed.2015.09.022. Epub 2016
4. Takagi, K. and Tagami, T., 2019. Work-style reform of emergency physicians: the Japanese experience. European Journal of Emergency Medicine, 26(6), pp.398-399.
5. Suriyawongpaisal P, Atiksawedparit P, Srithamrongsawad S, Thongtan T. Assessment of nationwide emergency systems in Thailand, a middle-income country setting with UHC. Int J Health Plann Manage. 2019 Apr;34(2):e1346-e1355. doi: 10.1002/hpm.2783. Epub 2019 Apr 4.
6. Sinclair D, Abu-Laban RB, Toth P, LeBlanc C, Eisener-Parsche P, Frank JR, Holroyd B.Emergency Medicine Training and Practice in Canada: Celebrating the Past & Evolving for the Future. Collaborative Working Group on the Future of Emergency Medicine in Canada CJEM. 2017 Jul;19(S2):S1-S8. doi: 10.1017/cem.2017.372.
7. The Royal College of Emergency Medicine and the NHS. Securing the future workforce for emergency departments in England. October 2017 https://improvement.nhs.uk/documents/1826/Emergency_department_workforce_plan_-_111017_Final.3.pdf Accessed July 31, 2019.
8. Hall MK, Burns K, Carius M et al. State of the National Emergency Department Workforce: Who Provides Care Where? *Ann Emerg Med*. 2018;72:302-307.
9. Gardner G, Gardner A, Middleton S et al. Mapping workforce configuration and operational models in Australian emergency departments: a national survey. *Austr Health Rev*. 2018;42:340-7.
10. Thrasher C, Purc-Stephenson R.J. Patient satisfaction with nurse practitioner care in emergency departments in Canada. *Am Acad Nurse Pract*. 2008;20:231-7.
11. Ritsema T. The Faculty of Physician Associates Census Results 2018. Faculty of Physician Associates at the Royal College of Physicians. http://www.fparcp.co.uk/about-fpa/fpa-census Updated date unknown. Accessed September 10, 2019.
12. Cawley JF, Hooker RS. Determinants of the physician assistant/associate concept in global health systems. *International Journal of Healthcare*. 2018;4:50-60.
13. Farmer J, Currie M, Hyman J et. al. Evaluation of physician assistants in National Health Service Scotland. *Scott Med J*. 2011;56:130-4.

For peer review only - http://bmjopen.bmj.com/site/about/guidelines.xhtml

14. Close B, Zolcinski R. Physician assistants in Australasian emergency departments. *Emerg Med Australas*. 2016;28:475-477.
15. Howie N. A new kid on the block: the role of physician associates. Clinical Medicine; 2015; 14, 6: 691–4 DOI: 10.7861/clinmedicine.15-3-309a ·
16. Doan Q, Sabhaney V, Kissoon N, et al. A systematic review: The role and impact of the physician assistant in the emergency department. *Emerg Med Australas*. 2011;23:7-15.
17. Halter M, Wheeler C, Pelone F, et al. Contribution of physician assistants/associates to secondary care: a systematic review. BMJ Open. 2018 8:e019573. doi: 10.1136/bmjopen-2017-019573.
18. Creswell JW, Clark VL. Designing and Conducting Mixed Methods Research. Thousand Oaks, CA: Sage Publications Ltd; 2007.
19. Drennan VM, Halter M, Wheeler C, et al. What is the contribution of physician associates in hospital care in England? A mixed methods, multiple case study *BMJ Open* 2019;9 e027012.
20. NHS Digital Provisional Accident and Emergency Quality Indicators - England, by provider for April 2018. Accessed 2-8-2018 https://digital.nhs.uk/data-and-information/publications/statistical/accident-and-emergency-quality-indicators/april-2018-by-provider
21. Mackway Jones K, Marsden J, Windle J. Emergency Triage: Manchester Triage Group, Third Edition . 2013. London: John Wiley & Sons.
22. Sakr M, Angus J, Perrin J, Nixon C, Nicholl J, Wardrope J. Care of minor injuries by emergency nurse practitioners or junior doctors: a randomised controlled trial. *Lancet* 1999;354:1321-6.
23. Cooper MA, Lindsay GM, Kinn S, Swann IJ. Evaluating Emergency Nurse Practitioner services: a randomized controlled trial. *J Adv Nurs*. 2002.40:721-30.
24. Pope C, Mays N. Observational Methods. In Pope C, Mays N, editors. Qualitative Research in Health Care. 4th edn. Chichester: John Wiley & Sons; 2008. pp. 32–42.
25. Kvale S. Doing Interviews. London: Sage Publications; 2007. https://doi.org/10.4135/9781849208963
26. McCann, L, Granter E, Hyde P, Hassard, J. Still Blue-Collar after all these Years? An Ethnography of the Professionalization of Emergency Ambulance Work. *Journal of Management Studies* 2013;50:750–776.
27. Boyatzis RE. *Transforming qualitative information: Thematic analysis and code development.* Thousand Oaks, Calif: Sage;1998.
28. Maxfield RG. Use of physician's assistants in a general surgical practice. The American Journal of Surgery. 1976;131:504–508. doi: 10.1016/0002-9610(76)90165-3.
29. Rodi SW, Grau MV, Orsini CM. Evaluation of a fast track unit: alignment of resources and demand results in improved satisfaction and decreased length of stay for emergency department patients. Quality Management in Healthcare. 2006;15:163–170. doi: 10.1097/00019514-200607000-00006.

For peer review only - http://bmjopen.bmj.com/site/about/guidelines.xhtml

30. Berg GM, Crowe RE, Nyberg S, Burdsal C. Trauma patient satisfaction with physician assistants: testing a structural equation model. Journal of the American Academy of PAs. 2012;25(5):42–43.
31. Fréchette D, Shrichand A. Insights into the physician assistant profession in Canada. *JAAPA* 2016;29:35–9.
32. Kartha A, Restuccia JD, Burgess JF, Benzer J, Glasgow J, Hockenberry J, et al. Nurse practitioner and physician assistant scope of practice in 118 acute care hospitals. *J Hosp Med* 2014;9:615–20.
33. Timmermans MJ, van Vught AJ, Maassen IT, Draaijer L, Hoofwijk AG, Spanier M, et al. Determinants of the sustained employment of physician assistants in hospitals: a qualitative study. *BMJ Open* 2016;6:e011949.
34. Williams K. Advanced practitioners in emergency care: a literature review. *Emergency Nurse* 2017;25:36-41.
35. Hinson JS, Martinez DA, Cabral S, et al. Triage Performance in Emergency Medicine: A Systematic Review. *Annals Emerg Med*; 2018;S0196-0644(18)31282-4.
36. General Medical Council. GMC to regulate two new associates roles. Press release 18-July-2019. https://www.gmc-uk.org/news/news-archive/gmc-to-regulate-two-new-associates-roles---pas-and-aas Accessed July 2019.

review only

For peer review only - http://bmjopen.bmj.com/site/about/guidelines.xhtml

Supplementary material

Supplementary material 1: Extract of the chart review dataset

Supplementary material 2: Proforma for the assessment of clinical adequacy

Supplementary material 3: Observation guide

Supplementary material 4: Tailored topic guides for the interview

Author Statement

VMD (PhD, health policy and service delivery research), MH (PhD, health services research), JP (MD, general practice and clinical education), HG (PhD, health economics), SdeL (MD[Res], general practice and information science), JG (PhD, medical sociology), SB (BSc, patient and public engagement), and PB (PhD, audiology and strategic management) conceived and designed the study and obtained research funding. VMD, MH, JP supervised the conduct of the study and data collection. VMD, MH, JP undertook recruitment of participating centers and managed the data, including quality. CWa (PhD, statistics) undertook the statistical analysis; CW (PhD, health services research), LN (PhD,health services research), MH, JE (MSc, physician associate and education) and VMD undertook qualitative data collection and thematic analysis and HG considered the economic aspects. MH drafted the manuscript, and all authors contributed substantially to its revision. VMD takes responsibility for the paper as a whole.

All authors attest to meeting the four ICMJE.org authorship criteria: (1) Substantial contributions to the conception or design of the work; or the acquisition, analysis, or interpretation of data for the work; AND (2) Drafting the work or revising it critically for important intellectual content; AND (3) Final approval of the version to be published; AND (4) Agreement to be accountable for all aspects of the work in ensuring that questions related to the accuracy or integrity of any part of the work are appropriately investigated and resolved.

The Patient and Public Involvement statement

The patient and public voice was important to this study and informed the design, conduct, analsyis, interpretation and final reporting. We brought the views of our public and patient representative forum for a previous study on physician associates into the research questions and design of the study. These were views such as how do patients understand this new role. Sally Brearley, as a public voice representative, was a co-applicant and member of the research team. The study advisory group had two public voice members who were reimbursed for their time, following NIHR INVOLVE guidance. Two patient and public voice groups were formed: one in London and the other in the West Midlands and members reimbursed as per NIHR Involve guidelines. The patient and public voice groups

For peer review only - http://bmjopen.bmj.com/site/about/guidelines.xhtml

informed the design of the research tools such as topic guides and participant information sheets, developed coding frameworks and analysed interview transcripts , and participated in the overall synthesis of findings. Sally Brearley continues to be involved in the dissemination of the study.

Acknowledgements

Our thanks to all those clinicians, administrative and information staff in the participating centers who assisted the study at a time of heightened workload within the emergency services in the National Health Service in England. Our thanks also to Robert Grant who provided statistical advice in the design of the study and obtained research funding but left the research team before the data were obtained for analysis.

Data statement

No additional data are available.

For peer review only

Supplementary file 1: Extract of the chart review dataset (first 50 cases, according to date of attendance)

study_id	sample_case	site_numb	age_formatted	sex_formatted	arrival_date	arrival_time	seen_time	presenting_complaint	mts	ews	ed_strean	professior	xrays_forr	pxn_forma	diagnosis	discharge_time	destination_grc	discharge_	discharge_	discharge_	reattend_	reattend_date
CS30115	0	3	5	0	05-Aug-16	9:10:00 PM	10:56:00 PM	Flank Pain(renal colic 3yrs ago)	#NULL!	#NULL!	1	1	#NULL!	#NULL!	Renal colic	2:07:00 AM	#NULL!	#NULL!	#NULL!		#NULL!	#NULL!
CS30197	0	3	6	1	05-Aug-16	7:54:00 PM	10:01:00 PM	Abdo/Back Pain 5/7	3	#NULL!	#NULL!	1	#NULL!	#NULL!	Generally unwell	1:40:00 AM	#NULL!	#NULL!	#NULL!		#NULL!	#NULL!
CS30415	0	3	4	1	05-Aug-16	4:51:00 PM	7:05:00 PM	21/40 ?DVT on clexane	3	#NULL!	#NULL!	1	#NULL!	#NULL!	Acute coronary syndrome	8:50:00 PM	#NULL!	#NULL!	#NULL!		#NULL!	#NULL!
CS30514	0	3	5	1	05-Aug-16	1:08:00 PM	2:15:00 PM	swelling to arm/hand ?clot	3	#NULL!	#NULL!	1	#NULL!	#NULL!	Deep venous thrombosis	5:07:00 PM	#NULL!	#NULL!	#NULL!		#NULL!	#NULL!
CS30726	0	3	3	0	05-Aug-16	11:45:00 PM	1:12:00 AM	HEADACHE	2	#NULL!	1	1	#NULL!	#NULL!	Unknown problem	1:58:00 AM	#NULL!	#NULL!	#NULL!		#NULL!	#NULL!
CS30962	0	3	4	0	05-Aug-16	4:26:00 PM	6:59:00 PM	Back Pain(mr maurice bulging disc)	3	#NULL!	#NULL!	0	#NULL!	#NULL!	Chronic back pain	8:20:00 PM	#NULL!	#NULL!	#NULL!		#NULL!	#NULL!
CS30993	0	3	4	1	05-Aug-16	6:57:00 AM	8:04:00 AM	Epigastric Pain 13months on/off	4	#NULL!	#NULL!	1	#NULL!	#NULL!	Acute gastritis	10:54:00 AM	#NULL!	#NULL!	#NULL!		#NULL!	#NULL!
CS31043	0	3	9	1	05-Aug-16	10:49:00 PM	12:00:00 AM	AP ?UTI	4	#NULL!	#NULL!	1	#NULL!	#NULL!	UTI - Urinary tract infection	2:13:00 AM	#NULL!	#NULL!	#NULL!		#NULL!	#NULL!
CS31165	0	3	3	1	05-Aug-16	9:56:00 PM	11:31:00 PM	PV Bleed+cramps(bloods with gp nad)	4	#NULL!	#NULL!	1	#NULL!	#NULL!	Menorrhagia	1:56:00 AM	#NULL!	#NULL!	#NULL!		#NULL!	#NULL!
CS31275	0	3	8	1	05-Aug-16	8:55:00 PM	10:50:00 PM	epigastric pain	3	#NULL!	1	0	#NULL!	#NULL!		12:38:00 AM	#NULL!	#NULL!	#NULL!		#NULL!	#NULL!
CS31564	0	3	1	0	05-Aug-16	4:26:00 PM	5:48:00 PM	not passing urine/feeding unsettled	3	#NULL!	#NULL!	0	#NULL!	#NULL!	Well child	6:52:00 PM	#NULL!	#NULL!	#NULL!		#NULL!	#NULL!
CS31572	0	3	3	0	05-Aug-16	11:20:00 PM	12:43:00 AM	Sickle cell attack	3	#NULL!	#NULL!	1	#NULL!	#NULL!	Sickle cell anemia crisis	3:18:00 AM	#NULL!	#NULL!	#NULL!		#NULL!	#NULL!
CS31639	0	3	8	1	05-Aug-16	8:53:00 AM	9:38:00 AM	HI, unwitnessed, stumbling on street	3	#NULL!	#NULL!	1	#NULL!	#NULL!	Head injury	12:50:00 PM	#NULL!	#NULL!	#NULL!		#NULL!	#NULL!
CS31640	0	3	8	1	05-Aug-16	10:24:00 AM	10:33:00 AM	FALL	3	#NULL!	#NULL!	1	#NULL!	#NULL!	Fall	2:23:00 PM	#NULL!	#NULL!	#NULL!		#NULL!	#NULL!
CS31641	0	3	3	1	05-Aug-16	10:46:00 AM	11:39:00 AM	FEELING FAINT	4	#NULL!	#NULL!	1	#NULL!	#NULL!	Fainting	2:03:00 PM	#NULL!	#NULL!	#NULL!		#NULL!	#NULL!
CS31642	0	3	1	0	05-Aug-16	3:04:00 PM	3:55:00 PM	unwell vomiting yellow	3	#NULL!	#NULL!	1	#NULL!	#NULL!	Vomiting - bile stained	6:01:00 PM	#NULL!	#NULL!	#NULL!		#NULL!	#NULL!
CS31644	0	3	8	1	05-Aug-16	8:17:00 PM	10:06:00 PM	abdo pain	#NULL!	#NULL!	1	1	#NULL!	#NULL!	Gastroenteritis	12:17:00 AM	#NULL!	#NULL!	#NULL!		#NULL!	#NULL!
CS32315	0	3	4	1	05-Aug-16	9:35:00 PM	11:24:00 PM	headaches	3	#NULL!	#NULL!	1	#NULL!	#NULL!	Headache	2:49:00 PM	#NULL!	#NULL!	#NULL!		#NULL!	#NULL!
CS32354	0	3	2	1	05-Aug-16	8:43:00 PM	10:24:00 PM	early pregnant, abdominal pain	3	#NULL!	#NULL!	1	#NULL!	#NULL!	Abdominal pain	12:18:00 AM	#NULL!	#NULL!	#NULL!		#NULL!	#NULL!
CS32524	0	3	9	1	05-Aug-16	2:48:00 PM	3:46:00 PM	FALL unwitnessed, unsteady (dementia)	3	#NULL!	#NULL!	1	#NULL!	#NULL!	Fall - accidental	6:44:00 PM	#NULL!	#NULL!	#NULL!		#NULL!	#NULL!
CS32671	0	3	6	0	05-Aug-16	3:10:00 PM	4:02:00 PM	?SEIZURE, hand was twitching (ca brain)	3	#NULL!	#NULL!	0	#NULL!	#NULL!	Partial seizure	7:10:00 PM	#NULL!	#NULL!	#NULL!		#NULL!	#NULL!
CS32693	0	3	8	1	05-Aug-16	10:20:00 PM	11:23:00 PM	RUQ Pain 3/7	4	#NULL!	1	1	#NULL!	#NULL!	Abdominal pain	2:19:00 AM	#NULL!	#NULL!	#NULL!		#NULL!	#NULL!
CS32713	0	3	9	0	05-Aug-16	4:09:00 PM	4:37:00 PM	UNWELL(bradycardia atropine given)	3	#NULL!	#NULL!	0	#NULL!	#NULL!	Acute confusion	8:09:00 PM	#NULL!	#NULL!	#NULL!		#NULL!	#NULL!
CS32840	0	3	7	0	05-Aug-16	11:06:00 AM	12:02:00 PM	SOB/Right sided CP (PE, pneumonia)	3	#NULL!	#NULL!	1	#NULL!	#NULL!	Pulmonary embolism	3:07:00 PM	#NULL!	#NULL!	#NULL!		#NULL!	#NULL!
CS32973	0	3	6	0	05-Aug-16	6:32:00 PM	9:01:00 PM	Paraphimosis	4	#NULL!	#NULL!	1	#NULL!	#NULL!	Paraphimosis	10:02:00 PM	#NULL!	#NULL!	#NULL!		#NULL!	#NULL!
CS33011	0	3	8	1	05-Aug-16	6:52:00 PM	8:55:00 PM	Suicidal/Intoxicated (etoh)	#NULL!	#NULL!	1	1	#NULL!	#NULL!	Alcohol intoxication (disorder)	10:51:00 PM	#NULL!	#NULL!	#NULL!		#NULL!	#NULL!
CS33793	0	3	7	1	05-Aug-16	2:25:00 PM	3:14:00 PM	COLLAPSED/AP ?seizure	3	#NULL!	#NULL!	1	#NULL!	#NULL!	Small bowel obstruction	6:25:00 PM	#NULL!	#NULL!	#NULL!		#NULL!	#NULL!
CS33926	0	3	7	1	05-Aug-16	11:44:00 AM	12:45:00 PM	painfull red eye]	2	#NULL!	#NULL!	1	#NULL!	#NULL!	Red eye	3:05:00 PM	#NULL!	#NULL!	#NULL!		#NULL!	#NULL!
CS34018	0	3	8	0	05-Aug-16	11:05:00 PM	12:34:00 AM	?UTI	3	#NULL!	#NULL!	1	#NULL!	#NULL!	Urinary tract infection	2:57:00 AM	#NULL!	#NULL!	#NULL!		#NULL!	#NULL!
CS34076	0	3	3	1	05-Aug-16	6:35:00 PM	8:54:00 PM	? chronns flare up/black stools	4	#NULL!	#NULL!	1	#NULL!	#NULL!	Crohn's disease (disorder)	10:34:00 PM	#NULL!	#NULL!	#NULL!		#NULL!	#NULL!
CS31018	1	3	9	1	05-Aug-16	6:26:00 PM	8:19:00 PM	SOB(inpatient langley green)	4	#NULL!	#NULL!	0	1	#NULL!	Pneumonia	4:57:00 AM	1	1.00	########	SPR	0	#NULL!
CS31643	1	3	5	0	05-Aug-16	8:03:00 PM	10:05:00 PM	Flank Pain(renal colic)	3	0	1	0	0	#NULL!	Left flank pain	11:13:00 PM	0	1.00	########	Consutlan	0	#NULL!
CS32456	1	3	7	0	05-Aug-16	7:27:00 PM	9:39:00 PM	sudden onset pain L testis	4	0	#NULL!	0	0	#NULL!	O/E - testicular swelling	11:23:00 PM	0	1.00	########	Consutlan	0	#NULL!
CS30038	0	3	7	0	06-Aug-16	7:45:00 PM	9:01:00 PM	2/7 hx CCP	3	#NULL!	#NULL!	1	#NULL!	#NULL!	Community acquired pneumonia	9:46:00 PM	#NULL!	#NULL!	#NULL!		#NULL!	#NULL!
CS30156	0	3	3	1	06-Aug-16	1:19:00 AM	3:08:00 AM	Headache took 24xParacetamol 500mg+4 kalms	2	#NULL!	1	1	#NULL!	#NULL!	Paracetamol overdose	7:39:00 AM	#NULL!	#NULL!	#NULL!		#NULL!	#NULL!
CS30320	0	3	5	0	06-Aug-16	1:55:00 AM	4:00:00 AM	Assault HI/Lft shoulder pain	4	#NULL!	1	1	#NULL!	#NULL!	Assault	5:55:00 AM	#NULL!	#NULL!	#NULL!		#NULL!	#NULL!
CS30351	0	3	3	1	06-Aug-16	2:02:00 AM	4:16:00 AM	Ear Infection(flucloxacillin)	4	#NULL!	#NULL!	1	#NULL!	#NULL!	Otitis media	5:25:00 AM	#NULL!	#NULL!	#NULL!		#NULL!	#NULL!
CS30392	0	3	7	0	06-Aug-16	7:08:00 PM	8:58:00 PM	R flank pain	4	#NULL!	#NULL!	1	#NULL!	#NULL!	Renal colic	10:24:00 PM	#NULL!	#NULL!	#NULL!		#NULL!	#NULL!
CS30398	0	3	3	0	06-Aug-16	7:00:00 PM	8:58:00 PM	?collapse - been drinking	3	#NULL!	#NULL!	1	#NULL!	#NULL!	Drunk	10:43:00 PM	#NULL!	#NULL!	#NULL!		#NULL!	#NULL!
CS30458	0	3	9	0	06-Aug-16	11:49:00 PM	1:47:00 AM	chest pain	3	#NULL!	#NULL!	1	#NULL!	#NULL!	Stable angina (disorder)	3:49:00 AM	#NULL!	#NULL!	#NULL!		#NULL!	#NULL!
CS30695	0	3	6	0	06-Aug-16	9:49:00 PM	12:02:00 AM	RTC 14hrs ago lower back pain,headache	4	#NULL!	#NULL!	1	#NULL!	#NULL!	Back pain	12:44:00 AM	#NULL!	#NULL!	#NULL!		#NULL!	#NULL!
CS30834	0	3	8	0	06-Aug-16	11:19:00 PM	12:55:00 AM	non epileptic seizure	3	#NULL!	#NULL!	1	#NULL!	#NULL!	Chronic confusion	10:13:00 AM	#NULL!	#NULL!	#NULL!		#NULL!	#NULL!
CS31121	0	3	3	1	06-Aug-16	9:08:00 PM	11:14:00 PM	OD/Mental health	#NULL!	#NULL!	#NULL!	1	#NULL!	#NULL!	Self-discharge	12:26:00 AM	#NULL!	#NULL!	#NULL!		#NULL!	#NULL!
CS31205	0	3	3	1	06-Aug-16	2:22:00 AM	3:53:00 AM	Passing small amounts urine	#NULL!	#NULL!	1	1	#NULL!	#NULL!	Renal colic	5:37:00 AM	#NULL!	#NULL!	#NULL!		#NULL!	#NULL!
CS31252	0	3	2	1	06-Aug-16	2:29:00 AM	5:03:00 AM	smoked cigarette.feels funny	4	#NULL!	1	1	#NULL!	#NULL!	Cigarette consumption	5:42:00 AM	#NULL!	#NULL!	#NULL!		#NULL!	#NULL!
CS31358	0	3	4	1	06-Aug-16	8:00:00 PM	9:39:00 PM	FLANK PAIN, KIDNEY STONES	4	#NULL!	#NULL!	1	#NULL!	#NULL!	Flank pain (finding)	12:00:00 AM	#NULL!	#NULL!	#NULL!		#NULL!	#NULL!
CS31534	0	3	3	1	06-Aug-16	12:45:00 AM	2:08:00 AM	Epigastric Pain	4	#NULL!	1	1	#NULL!	#NULL!	Gastritis	4:39:00 AM	#NULL!	#NULL!	#NULL!		#NULL!	#NULL!
CS31607	0	3	3	1	06-Aug-16	1:41:00 AM	3:08:00 AM	Abdo/back Pain	4	#NULL!	#NULL!	1	#NULL!	#NULL!	Palpitations - fluttering	4:37:00 AM	#NULL!	#NULL!	#NULL!		#NULL!	#NULL!
CS31624	0	3	4	0	06-Aug-16	11:32:00 PM	12:55:00 AM	?Cellulitis/Ulcers Bilateral	2	#NULL!	#NULL!	1	#NULL!	#NULL!	Cellulitis	3:32:00 AM	#NULL!	#NULL!	#NULL!		#NULL!	#NULL!

Supplementary material 2, assessment of clinical adequacy questions

Excel spread sheet sent to each reviewer to complete for all records. One column for each record – reviewer to fill in id number

	Questions *	Insert study ID number from top of record<br>*Drop down response options followed by a cell for free text if appropriate*
1	Record of the patient's medical history	Appropriate<br>Or<br>Error or omission – but unlikely to have resulted in harm or different treatment<br>Or<br>An error or omission seen that caused a breach in normal guidelines and procedures that would have altered the patient's treatment<br>Or<br>An error or omission seen that resulted in significant probability that the patient might be harmed
	Free text on rationale or comment on response to item 1	
2	Examination of the patient	Appropriate<br>Or<br>Error or omission – but unlikely to have resulted in harm or different treatment<br>Or<br>An error or omission seen that caused a breach in normal guidelines and procedures that would have altered the patient's treatment<br>Or<br>An error or omission seen that resulted in significant probability that the patient might be harmed
	Free text on rationale or comment on response to item 2	
3	Request for radiography	Appropriate<br>Or<br>Error or omission – but unlikely to have resulted in harm or different treatment<br>Or<br>An error or omission seen that caused a breach in normal guidelines and procedures that would have altered the patient's treatment<br>Or<br>An error or omission seen that resulted in significant probability that the patient might be harmed
	Free text on rationale or comment on response to item 3	
4	Treatment plan and decision	Appropriate<br>Or<br>Error or omission – but unlikely to have resulted in harm or different treatment<br>Or<br>An error or omission seen that caused a breach in normal guidelines and procedures that would have altered the patient's treatment<br>Or<br>An error or omission seen that resulted in significant probability that the patient might be harmed

For peer review only - http://bmjopen.bmj.com/site/about/guidelines.xhtml

	Questions *	Insert study ID number from top of record<br>*Drop down response options followed by a cell for free text if appropriate*
	Free text on rationale or comment on response to item 4	
5	Treatment plan and decision reviewed by senior doctor	YES<br>Or<br>NO
6	Advice given	Appropriate<br>Or<br>Error or omission – but unlikely to have resulted in harm or different treatment<br>Or<br>An error or omission seen that caused a breach in normal guidelines and procedures that would have altered the patient's treatment<br>Or<br>An error or omission seen that resulted in significant probability that the patient might be harmed
	Free text on rationale or comment on response to item 6	
7	Follow-up	Appropriate<br>Or<br>Error or omission – but unlikely to have resulted in harm or different treatment<br>Or<br>An error or omission seen that caused a breach in normal guidelines and procedures that would have altered the patient's treatment<br>Or<br>An error or omission seen that resulted in significant probability that the patient might be harmed
	Free text on rationale or comment on response to item 7	
8	In your view what type of clinician attended this patient?	Doctor<br>Or<br>Physician associate<br>Or<br>Unable to decide
	Free text on rationale or comment on response to item 8	

* Review questions taken from Sakr et al study [17] comparing patients attended by advanced nurse practitioners with doctors in the ED.

For peer review only

Supplementary file 3: Observation guide

OBSERVATION: AIDE MEMOIRE for researchers

Our observation aims to support answering the four study research questions but specifically to provide data on impact on organisation of services, other team members working practices and team relationships.

We wish to be able to consider this in terms of:

- Acceptability -how do they appear to be viewed or treated by others?
- Appropriateness – how are they observed in terms of safety e.g. how do they check, how are they checked upon, how are they supervised?
- Equity - who receives the PA service; do any patient groups appear to be over represented?
- Efficiency - how do they appear to contribute to this? How are issues, such as prescribing, worked around?
- Effectiveness – are the outcomes of PA care or contribution to the team observed?

We are observing context, relationships and activities.

For peer review only

Conduct of the observation

- Put up approved notices of our observation activity in the study setting places advised by the clinical team
- Provide the PA with the approved script to inform patients/ patients' representatives to gain permission for the researcher's presence. Each patient is to be asked for permission.
- Researcher to maintain an unobtrusive presence
- Record observations in the ethnographic tradition - take detailed unstructured notes, bearing in mind the importance of capturing context, relationships and activities
- Record as much as possible at the time and add as soon as possible afterwards
- Length of observation to be pre-planned but also to allow for flexibility according to the PA's wishes, the demands of the clinical setting and researcher's length of focus
- Allow the PA to see the notes at any point

After the observation

- Add to the notes as soon as possible where detail was not able to be captured at the time
- Maintain a reflective diary associated with the observation conduct and analytical processes.
- Discuss the observation with local research team members to promote group understanding and consistency across researchers
- Transfer data into NVivo software.

For peer review only - http://bmjopen.bmj.com/site/about/guidelines.xhtml

Supplementary data file 4: Tailored topic guides for the interview

Topic guide for senior managers and clinicians

Topic areas

- Confirm the person's job role
- Ask them to describe their involvement with physician associate employment in the hospital to date
- Ask questions on the factors supporting the adoption of the employment of physician associates
- Ask questions on the factors inhibiting the employment of physician associates
- Questions on their views of physician associates' impact on (ask for examples):
    - Organisation of services
    - Patient experience and outcomes
    - Other staff
    - Costs
- Anything else they would like to say?

Interviewer to probe on all answers to ensure the meaning is clear (e.g. that is an interesting point , can you explain a bit more about it) and check for understanding (e.g. so can I check I have understood you correctly)…….

Interviewer to check for any routine management reports or data or evaluations that the hospital team would be willing to share with the researchers.

Thank them and ask if they would like to receive updates on the study and a final summary of the findings. If so could they please give contact details which will be kept separate from the interview data.

For peer review only

Topic guide for physician associate interviews

Topic areas

- Ask them to describe how long they have been a physician associate, how many posts, type and length as a physician associate
- Ask them to describe the work they undertake, with what type of medical/surgical team
- Ask about their supervising doctor and arrangements when they are not there
- Ask questions on their views of the factors supporting the adoption of the employment of physician associate in their experience

For peer review only - http://bmjopen.bmj.com/site/about/guidelines.xhtml

- Ask questions on their views of the factors inhibiting the employment of physician associate in their experience
- Ask how they have been received in the hospital as a new type of health professional?
- Ask how they explain to patients, family and staff – who they are and what a physician associate is

- Questions on their views of their, or other physician associates, impact on (ask for examples):
    - Organisation of services
    - Patient experience and outcomes
    - Other staff
    - Costs

- Anything else they would like to say?

Interviewer to probe on all answers to ensure the meaning is clear (e.g that is an interesting point , can you explain a bit more about it) and check for understanding (e.g. so can I check I have understood you correctly)…….

Interviewer to check for any routine management reports or data or evaluations that the hospital team would be willing to share with the researchers .

Thank them and ask if they would like to receive updates on the study and a final summary of the findings. If so could they please give contact details which will be kept separate from the interview data.

Topic guide for all other types of professionals/managers interviews

- Confirm the person's job role
- Ask them to describe their involvement with physician associate employment in the hospital to date
- Ask questions on their views of any factors supporting the adoption of the employment of physician associates in their experience
- Ask questions on their views of any factors inhibiting the employment of physician associates in their experience
- Ask their views as to how the PAs have been received in that service/team, and probe for any explanations

For peer review only - http://bmjopen.bmj.com/site/about/guidelines.xhtml

- Questions on their views of physician associates' impact on (ask for examples):
    - Organisation of services
    - Boundaries between the job roles of different types of professionals e.g. with nurses
    - Patient experience and outcomes
    - Other staff
    - Costs

- Anything else they would like to say?

Interviewer to probe on all answers to ensure the meaning is clear (e.g that is an interesting point , can you explain a bit more about it) and check for understanding (e.g. so can I check I have understood you correctly)…….

Interviewer to check for any routine management reports or data or evaluations that the hospital team would be willing to share with the researchers.

Thank them and ask if they would like to receive updates on the study and a final summary of the findings. If so could they please give contact details which will be kept separate from the interview data.

Topic guide for patient interviews

<u>Topic areas</u>

- Confirm the person is/has been a patient
- Ask them to outline the type of care they have been in receipt of without giving personal medical details e.g. in patient for x days
- Confirm the patient has met the physician associate
- Explore what sort of involvement the physician associate has had with them
- Ask them how they understand the role of the physician associate in the medical/surgical team
- Ask them how they found receiving care from a physician associate
- If they were to need similar medical or surgical care, would they be content to receive similar care from a physician associate in the future as they had this time (and can

For peer review only - http://bmjopen.bmj.com/site/about/guidelines.xhtml

they explain why) or would they prefer someone different? And if yes, can they explain why?

- Anything else they would like to say?

Interviewer to probe on all answers to ensure the meaning is clear (e.g. that is an interesting point , can you explain a bit more about it) and check for understanding (e.g. so can I check I have understood you correctly).

Thank them and ask if they would like to receive updates on the study and a final summary of the findings. If so could they please give contact details which will be kept separate from the interview data.

For peer review only

O'Cathain A, Murphy E, Nicholl J. The quality of mixed methods studies in health services research. J Health Serv Res Policy. 2008;13(2):92-98.

Box 1 Good Reporting of A Mixed Methods Study (GRAMMS)

GRAMMS criterion	Page in manuscript
(1) Describe the justification for using a mixed methods approach to the research question	8
(2) Describe the design in terms of the purpose, priority and sequence of methods	8
(3) Describe each method in terms of sampling, data collection and analysis	8-10
(4) Describe where integration has occurred, how it has occurred and who has participated in it	10
(5) Describe any limitation of one method associated with the present of the other method	18-19
(6) Describe any insights gained from mixing or integrating methods	18-19

For peer review only

No.	Topic	Item
	**Title and abstract**	
S1	Title	Concise description of the nature and topic of the study Identifying the study as qualitative or indicating the approach (e.g., ethnography, grounded theory) or data collection methods (e.g., interview, focus group) is recommended
S2	Abstract	Summary of key elements of the study using the abstract format of the intended publication; typically includes background, purpose, methods, results, and conclusions
	**Introduction**	
S3	Problem formulation	Description and significance of the problem/phenomenon studied; review of relevant theory and empirical work; problem statement
S4	Purpose or research question	Purpose of the study and specific objectives or questions
	**Methods**	
S5	Qualitative approach and research paradigm	Qualitative approach (e.g., ethnography, grounded theory, case study, phenomenology, narrative research) and guiding theory if appropriate; identifying the research paradigm (e.g., postpositivist, constructivist/ interpretivist) is also recommended; rationale[b]
S6	Researcher characteristics and reflexivity	Researchers' characteristics that may influence the research, including personal attributes, qualifications/experience, relationship with participants, assumptions, and/or presuppositions; potential or actual interaction between researchers' characteristics and the research questions, approach, methods, results, and/or transferability
S7	Context	Setting/site and salient contextual factors; rationale[b]
S8	Sampling strategy	How and why research participants, documents, or events were selected; criteria for deciding when no further sampling was necessary (e.g., sampling saturation); rationale[b]
S9	Ethical issues pertaining to human subjects	Documentation of approval by an appropriate ethics review board and participant consent, or explanation for lack thereof; other confidentiality and data security issues
S10	Data collection methods	Types of data collected; details of data collection procedures including (as appropriate) start and stop dates of data collection and analysis, iterative process, triangulation of sources/methods, and modification of procedures in response to evolving study findings; rationale[b]
S11	Data collection instruments and technologies	Description of instruments (e.g., interview guides, questionnaires) and devices (e.g., audio recorders) used for data collection; if/how the instrument(s) changed over the course of the study
S12	Units of study	Number and relevant characteristics of participants, documents, or events included in the study; level of participation (could be reported in results)
S13	Data processing	Methods for processing data prior to and during analysis, including transcription, data entry, data management and security, verification of data integrity, data coding, and anonymization/deidentification of excerpts
S14	Data analysis	Process by which inferences, themes, etc., were identified and developed, including the researchers involved in data analysis; usually references a specific paradigm or approach; rationale [b]
S15	Techniques to enhance trustworthiness	Techniques to enhance trustworthiness and credibility of data analysis (e.g., member checking, audit trail, triangulation); rationale[b]
	**Results/findings**	
S16	Synthesis and interpretation	Main findings (e.g., interpretations, inferences, and themes); might include development of a theory or model, or integration with prior research or theory
S17	Links to empirical data	Evidence (e.g., quotes, field notes, text excerpts, photographs) to substantiate analytic findings
	**Discussion**	
S18	Integration with prior work, implications, transferability, and contribution(s) to the field	Short summary of main findings; explanation of how findings and conclusions connect to, support, elaborate on, or challenge conclusions of earlier scholarship; discussion of scope of application/ generalizability; identification of unique contribution(s) to scholarship in a discipline or field
S19	Limitations	Trustworthiness and limitations of findings
	**Other**	
S20	Conflicts of interest	Potential sources of influence or perceived influence on study conduct and conclusions; how these were managed
S21	Funding	Sources of funding and other support; role of funders in data collection, interpretation, and reporting

[a]The authors created the SRQR by searching the literature to identify guidelines, reporting standards, and critical appraisal criteria for qualitative research; reviewing the reference lists of retrieved sources; and contacting experts to gain feedback. The SRQR aims to improve the transparency of all aspects of qualitative research by providing clear standards for reporting qualitative research.

[b]The rationale should briefly discuss the justification for choosing that theory, approach, method, or technique rather than other options available, the assumptions and limitations implicit in those choices, and how those choices influence study conclusions and transferability. As appropriate, the rationale for several items might be discussed together.

ACADEMIC MEDICINE

S1 p3 (MMR cannot fit in more detail in title)

S2 P3

S3 P7

S4 P7

S5 P8, 9

S6 P10

S7 P8

S8 P9-10

S9 P10

S10 P9-10

S11 P10 (refers to detail elsewhere)

S12 P11

S13 P10 (refers to detail elsewhere)

S14 P10 (refers to detail elsewhere)
S14 As above

S16 P11-17, embedded alongside quant
S17 As above

S18 P17-19

S19 P18-19

S20&21 P5

only

For peer review only

The RECORD statement: Checklist of items, extended from the STROBE statement, that should be reported in observational studies using routinely collected health data.

	Item Number	STROBE Items	RECORD Items	Page number
**Title and Abstract**				
	1	(a) Indicate the study's design with a commonly used term in the title or the abstract. (b) Provide in the abstract an informative and balanced summary of what was done and what was found.	RECORD 1.1: The type of data used should be specified in the title or abstract. When possible, the name of the databases used should be included. RECORD 1.2: If applicable, the geographic region and time frame within which the study took place should be reported in the title or abstract. RECORD 1.3: If linkage between databases was conducted for the study, this should be clearly stated in the title or abstract.	3
**Introduction**				
Background rationale	2	Explain the scientific background and rationale for the investigation being reported.		7
Objectives	3	State specific objectives, including any prespecified hypotheses.		7
**Methods**				
Study Design	4	Present key elements of study design early in the paper.		8
Setting	5	Describe the setting, locations, and relevant dates, including periods of recruitment, exposure, follow-up, and data collection.		8
Participants	6	*(a) Cohort study*: Give the eligibility criteria and the sources and methods of selection of participants. Describe methods of follow-up. *Case-control study*: Give the eligibility criteria and the sources and methods of case ascertainment and control selection. Give the rationale for the choice of cases and controls. *Cross-sectional study*: Give the eligibility criteria and the sources and methods of	RECORD 6.1: The methods of study population selection (such as codes or algorithms used to identify subjects) should be listed in detail. If this is not possible, an explanation should be provided. RECORD 6.2: Any validation studies of the codes or algorithms used to select the population should be referenced. If validation was conducted for this study and not published	8

For peer review only

	Item Number	STROBE Items	RECORD Items	Page number
		selection of participants. *(b) Cohort study*: For matched studies, give matching criteria and number of exposed and unexposed. *Case-control study*: For matched studies, give matching criteria and the number of controls per case.	elsewhere, detailed methods and results should be provided. RECORD 6.3: If the study involved linkage of databases, consider use of a flow diagram or other graphical display to demonstrate the data linkage process, including the number of individuals with linked data at each stage.	
Variables	7	Clearly define all outcomes, exposures, predictors, potential confounders, and effect modifiers. Give diagnostic criteria, if applicable.	RECORD 7.1: A complete list of codes and algorithms used to classify exposures, outcomes, confounders, and effect modifiers should be provided. If these cannot be reported, an explanation should be provided.	10, 12
Data sources/measurement	8	For each variable of interest, give sources of data and details of methods of assessment (measurement). Describe comparability of assessment methods if there is more than one group.		8,10
Bias	9	Describe any efforts to address potential sources of bias.		10
Study size	10	Explain how the study size was arrived at.		9
Quantitative variables	11	Explain how quantitative variables were handled in the analyses. If applicable, describe which groupings were chosen and why.		8,10
Statistical methods	12	(a) Describe all statistical methods, including those used to control for confounding. (b) Describe any methods used to examine subgroups and interactions. (c) Explain how missing data were addressed. (d) *Cohort study*: If applicable, explain how loss to follow-up was addressed. *Case-control study*: If applicable, explain how matching of cases and controls was addressed. *Cross-sectional study*: If applicable, describe analytical methods taking account of sampling strategy. (e) Describe any sensitivity analyses.		10
Data access and cleaning methods		N/A	RECORD 12.1: Authors should describe the extent to which the investigators had access to the database population used to create the study population. RECORD 12.2: Authors	8-9

For peer review only

	Item Number	STROBE Items	RECORD Items	Page number
			should provide information on the data cleaning methods used in the study.	
Linkage		N/A	RECORD 12.3: State whether the study included person-level, institutional-level, or other data linkage across two or more databases. The methods of linkage and methods of linkage quality evaluation should be provided.	8-9
**Results**				
Participants	13	(a) Report the numbers of individuals at each stage of the study (e.g., numbers potentially eligible, examined for eligibility, confirmed eligible, included in the study, completing follow-up, and analysed). (b) Give reasons for nonparticipation at each stage. (c) Consider use of a flow diagram.	RECORD 13.1: Describe in detail the selection of the persons included in the study (i.e., study population selection), including filtering based on data quality, data availability, and linkage. The selection of included persons can be described in the text and/or by means of the study flow diagram.	11, 12, 13
Descriptive data	14	(a) Give characteristics of study participants (e.g., demographic, clinical, and social) and information on exposures and potential confounders. (b) Indicate the number of participants with missing data for each variable of interest. (c) *Cohort study*: summarise follow-up time (e.g., average and total amount).		12
Outcome data	15	*Cohort study*: Report numbers of outcome events or summary measures over time. *Case-control study*: Report numbers in each exposure category or summary measures of exposure. *Cross-sectional study*: Report numbers of outcome events or summary measures.		13
Main results	16	(a) Give unadjusted estimates and, if applicable, confounder-adjusted estimates and their precision (e.g., 95% confidence interval). Make clear which confounders were adjusted for and why they were included. (b) Report category boundaries when continuous variables were categorized. (c) If relevant, consider translating estimates of relative risk into absolute risk for a meaningful time period.		13,14

For peer review only

	Item Number	STROBE Items	RECORD Items	Page number
Other analyses	17	Report other analyses done—e.g., analyses of subgroups and interactions and sensitivity analyses		15
**Discussion**				
Key results	18	Summarise key results with reference to study objectives.		17-18
Limitations	19	Discuss limitations of the study, taking into account sources of potential bias or imprecision. Discuss both direction and magnitude of any potential bias.	RECORD 19.1: Discuss the implications of using data that were not created or collected to answer the specific research question(s). Include discussion of misclassification bias, unmeasured confounding, missing data, and changing eligibility over time, as they pertain to the study being reported.	19
Interpretation	20	Give a cautious overall interpretation of results considering objectives, limitations, multiplicity of analyses, results from similar studies, and other relevant evidence.		19
Generalisability	21	Discuss the generalisability (external validity) of the study results.		18-19
**Other Information**				
Funding	22	Give the source of funding and the role of the funders for the present study and, if applicable, for the original study on which the present article is based.		5
Accessibility of protocol, raw data, and programming code		N/A	RECORD 22.1: Authors should provide information on how to access any supplemental information such as the study protocol, raw data, or programming code.	24

N/A, not applicable

For peer review only
